# Patient-reported outcomes among people living with HIV on single- versus multi-tablet regimens: Data from a real-life setting

**DOI:** 10.1371/journal.pone.0262533

**Published:** 2022-01-13

**Authors:** Sophie Degroote, Linos Vandekerckhove, Dirk Vogelaers, Charlotte Vanden Bulcke

**Affiliations:** 1 Department of General Internal Medicine, Ghent University Hospital, Ghent, Belgium; 2 Department of Internal Medicine and Paediatrics, Ghent University, Ghent, Belgium; 3 Department of General Internal Medicine, AZ Delta, Roeselare, Belgium; 4 Department of Physical Medicine and Rehabilitation, Ghent University Hospital, Ghent, Belgium; Universitas Indonesia Fakultas Kedokteran, INDONESIA

## Abstract

**Background:**

The use of single-tablet regimens (STRs) in HIV treatment is ubiquitous. However, reintroducing the (generic) components as multi-tablet regimens (MTRs) could be an interesting cost-reducing strategy. It is essential to involve patient-reported outcome measures (PROs) to examine the effects of such an approach. Hence, this study compared PROs of people living with HIV taking an STR versus a MTR in a real world setting.

**Materials and methods:**

This longitudinal study included 188 people living with HIV. 132 remained on a MTR and 56 switched to an STR. At baseline, months 1-3-6-12-18 and 24, participants filled in questionnaires on health-related quality of life (HRQoL), depressive symptoms, HIV symptoms, neurocognitive complaints (NCC), treatment satisfaction and adherence. Generalized linear mixed models and generalized estimation equations mixed models were built.

**Results:**

Clinical parameters and PROs of the two groups were comparable at baseline. Neurocognitive complaints and treatment satisfaction did differ over time among the groups. In the STR-group, the odds of having NCC increased monthly by 4,1% as compared to the MTR-group (p = 0.035). Moreover, people taking an STR were more satisfied with their treatment after 6 months: the median change score was high: 24 (IQR 7,5–29). Further, treatment satisfaction showed a contrary evolution in the groups: the estimated state score of the STR-group increased by 3,3 while it decreased by 0,2 in the MTR-group (p = 0.003). No differences over time between the groups were observed with regard to HRQoL, HIV symptoms, depressive symptoms and adherence.

**Conclusions:**

Neurocognitive complaints were more frequently reported among people on an STR versus MTR. This finding contrasts with the higher treatment satisfaction in the STR-group over time. The long-term effects of both PROs should guide the decision-making on STRs vs. (generic) MTRs.

## Introduction

The past decades have been dominated by positive evolutions in the treatment of people living with HIV (PLHIV). Contemporary antiretroviral therapy (ART) is well tolerated and much more convenient due to newer, safer drugs and the use of co-formulations, which reduce pill burden and dosing frequency. Simplification of medication regimens has been the ground for the development of single-tablet regimens (STRs), i.e. a complete cocktail of three (or two) antiretroviral agents in one pill per day. STRs demonstrate a high efficacy, good tolerability and are associated with good adherence and treatment satisfaction. STRs are therefore increasingly used among PLHIV around the world [1].

The high efficacy of STRs is beyond question. Virologic suppression after one year is higher among STR-starters than among multi-tablet regimen (MTR) starters [2], even when only once-daily MTRs are considered [3]. Clay et al. compared 48 week outcomes between STRs and MTRs in a meta-analysis and found that viral suppression was more likely in the STR-group [4]. On the other hand, a large French cohort study, not included in the meta-analysis of Clay et al., found no difference in virological efficacy between STRs and MTRs in HIV-naive patients [5]. Another recent Italian cohort study reported similar virological control among PLHIV starting an STR and those starting a 2-pills, once daily MTR [6].

Successful virological control, however, does not only rely on the potency of the medication, but also on medication adherence. Adherence was significantly higher among patients taking STRs as compared to MTRs [4]. Even when STRs were compared to once daily MTRs, adherence in the former group remained significantly higher [4]. A meta-analysis including only observational (‘real-world’) studies confirmed better adherence in STRs as compared to MTRs [7]. In Brazil, there was a 14% increase in proportion of adherent patients during 18 months follow-up among participants who were switched from a MTR to an STR with the same compounds [8]. Discontinuation of ART, as a proxy for persistence, was higher among MTR-users, but this is difficult to interpret because reasons for discontinuation also include regimen simplification, which, logically, occurs more often among the (more complex) MTR-regimens [4, 5, 9, 10].

Beyond viral suppression, there is yet another ambition to take into account: ensuring a good health-related quality of life (HRQoL) among PLHIV [11]. Research on HRQoL and other patient-reported outcomes (PROs) among STR- versus MTR-users is rather scarce. People who switched from a MTR to an STR had an improved HRQoL after six months, however, this study had no control group [12]. Only one study in the meta-analysis of Clay et al. [4] directly compared patient-reported outcomes between STRs and MTRs [13]. Treatment satisfaction appeared to be higher among people taking an STR. No differences in HRQoL were found between both groups [13]. More recently, Costa et al. reported no HRQoL differences in between STR- and MTR-starters [14].

As health care resources are restricted, not only the effects of therapy need to be addressed, but also the costs. Different studies have assessed the costs of STRs; they appear to be cost-saving [15] and most studies consider STRs to be more cost-effective than MTRs as compared to ‘no therapy’ [16, 17] and even cost-effective as compared to generic MTRs [18].

The cost story becomes even more relevant with the advent of generic ART, which are also associated with good outcomes [19–21] and can be cost-saving [22–28]. Nonetheless, the use of generic ART in high-income countries remains meagre. The number of generic STRs is limited. In practice, prescribing generics means ‘de-simplifying’ STRs, which raises concerns about poorer outcomes by increasing the number of pills or the dosing frequency [29]. In Spain, only 20% of the hospitals used de-simplified STRs [30]. Studies found high percentages of PLHIV who were willing to switch to MTRs [31, 32], but a French study found that only 17% of the patients would accept generic ART if the number of pills per day would increase [33].

In short, STRs have become standard in ART but it is unclear if their widespread use and higher costs are endorsed by better PROs. The aim of this observational two-year follow-up study was to compare PROs over time among treatment-experienced patients who 1) switched to an STR or 2) remained on their MTR.

## Materials and methods

### Setting

The study was conducted at the HIV Reference Centre of Ghent University Hospital (Belgium), a service involved in the medical treatment and emotional and social support for PLHIV. One thousand five hundred PLHIV are currently followed by a multidisciplinary team of physicians, (social) nurses, a psychologist, a sex therapist and a dietician. The HIV epidemic in Belgium is subdued, with a stable incidence and the achievement of the UNAIDS 90-90-90 ambition in 2018: out of 18.335 PLHIV in Belgium, 91% is diagnosed, 92% of them receives ART and 94% of them has an undetectable viral load [34, 35]. The so-called ‘fourth 90’, 90% of virally suppressed PLHIV with a good HRQoL [11], seems to be challenging [36].

### Participants

Between January 2016 and June 2017, treatment-experienced patients who switched to an STR and treatment-experienced patients who remained on their MTR were included in the study. Following inclusion criteria were applied: age ≥ 18 years, mastery of Dutch or French in order to be able to fill in the self-report questionnaires and having signed the written informed consent form. The study was approved by the institutional review board of Ghent University Hospital (Belgian Registration number B670201523485).

### Patient-reported outcomes

Various patient-reported outcomes were collected: HRQol, treatment satisfaction, neurocognitive complaints (NCC), adherence, depressive symptoms and HIV symptoms (Table 1).

**Table 1:** Patient-reported outcomes collected in the study.

Outcome	Instrument	Range	Time of assessment	Reference
HRQoL	Medical Outcomes Study (MOS)-HIV: Physical health score (PHS)	0–100	All timepoints	[37]
	MOS-HIV: Mental health score (MHS)	0–100	All timepoints	
	EuroQol 6Q-3L: utility score	0–1	All timepoints	[38]
	EuroQol visual analogue scale (VAS)	0–100	All timepoints	
Treatment satisfaction	HIV Treatment Satisfaction Questionnaire: state score	0–60	T0,T4,T5,T6 in both groups	[39]
	HIV Treatment Satisfaction Questionnaire: change score	-30–30	T3 in STR-group	
Neurocognitive complaints	3 screening questions (memory, reasoning/planning/solving problems and attention)	In case of one or more affirmative answers: presence of NCC	All timepoints	[40]
Adherence	Center for Adherence Support Evaluation (CASE) Adherence Index: sum score	0–16	All timepoints	[41]
	Visual analogue scale (VAS)	0–100	All timepoints	
Depressive symptoms	Beck Depression Inventory: sum score	0–63	All timepoints	[42]
HIV Symptoms	HIV Symptom Index (0–20)	0–20	All timepoints	[43]

The PROs were measured through self-report questionnaires at seven time points: baseline (T0, in the STR-group this was the moment of switch), month 1 (T1), month 3 (T2), month 6 (T3), month 12 (T4), month 18 (T5) and finally month 24 (T6).

### Statistical analyses

STR- and MTR-participants’ socio-demographic and clinical baseline measures were compared by means of chi-square tests and Mann-Whitney U tests. Mixed models were built for the continuous outcomes listed in Table 1. For neurocognitive complaints, which was a dichotomous outcome measure, a generalized estimating equations model was built. We controlled for baseline differences between the STR and MTR group (see Table 2) by adding gender, sexual orientation and ethnicity as covariates in every model. Pairwise comparisons of the estimates between the groups were also performed. An ‘intention to treat’ approach was applied, i.e. data from participants who shifted from regimen during the study remained included in the original group. Only data from participants with two or more present values over time (≥2/7) for the given outcome were included in the models. To account for missing data, sensitivity analyses were performed. A ‘best-worst case scenario’ and a ‘worst-best case scenario’ were created and corresponding models were built [44]. Detailed information on how the models were built, the estimates of fixed effects, estimates and graphs for each model, as well as the sensitivity analyses can be found in S1–S3 Files.

**Table 2:** Participants’ socio-demographic and clinical data.

	STR (n = 56)		MTR (n = 132)		p-value
	Number	%	Number	%	
Sex					0.046
Male	40	71.4	111	84.1	
Female	16	28.6	21	15.9	
Ethnicity					0.016
Caucasian	47	83.9	125	94.7	
Non-Caucasian	9	16.1	7	5.3	
Activity					0.231
Working	41	73.2	90	68.2	
Student	2	3.6	0	0.0	
Seeking work	1	1.8	4	3.0	
Houseman/housewife	0	0	2	1.5	
Retired	5	8.9	19	14.4	
Invalid	7	12.5	17	12.9	
Sexual orientation					0.022
Homosexual	26	46.4	89	67.5	
Bisexual	3	5.4	6	4.5	
Heterosexual	27	48.2	37	28.0	
Ever AIDS					1
No	42	75.0	99	75.0	
Yes	14	25.0	33	25.0	
	Median (years)	IQR	Median (years)	IQR	
Age	46	39–55	48.5	41–56	0.348
Time since diagnosis	9	5–14.75	9	5–16	0.509
Time since start ART	7	4–11	7	4–14.75	0.364

## Results

### Participants

A total of 188 participants took part in the study: 132 in the MTR-group and 56 in the STR-group. Their baseline socio-demographic and clinical data are summarized in Table 2. ART regimens are listed in Table 3. Among the MTR-group, 106 participants took a once-daily regimen, the other 26 participants took a twice-daily regimen. The majority (n = 62) took two pills per day, 50 participants took three pills per day, 20 participants took more than three pills per day. During the study, 5 MTR-participants switched to an STR, 3 STR-participants switched to their previous MTR. Two patients died (one in each group).

**Table 3:** Participants’ ART-regimens.

MTR-group (n = 131)		
**NNRTI+NNRTI**		**37**
Abacavir/Lamivudine, Nevirapine	16	
Emtricitabine/Tenofovir, Nevirapine	11	
Abacavir/Lamivudine, Efavirenz	7	
Abacavir/Lamivudine, Rilvipirine	1	
Emtricitabine/Tenofovir, Efavirenz	1	
Abacavir, Tenofovir, Efavirenz	1	
**NRTI+INT**		**33**
Emtricitabine/Tenofovir, Dolutegravir	11	
Emtricitabine/Tenofovir, Raltegravir	7	
Emtricitabine, Dolutegravir	5	
Emtricitabine/Tenofovir Alafenamide, Dolutegravir	4	
Abacavir/Lamivudine, Raltegravir	3	
Lamivudine, Dolutegravir	1	
Lamivudine, Tenofovir, Dolutegravir	1	
Abacavir/Lamivudine, Tenofovir, Dolutegravir	1	
**NRTI+PI**		**26**
Emtricitabine/Tenofovir, Darunavir, Norvir	11	
Emtricitabine/Tenofovir, Atazanavir, Norvir	7	
Abacavir/Lamivudine, Atazanavir, Norvir	5	
Abacavir/Lamivudine, Darunavir, Norvir	2	
Abacavir/Lamivudine, Atazanavir	1	
**NNRTI+INT**		**10**
Nevirapine, Dolutegravir	4	
Rilvipirine, Dolutegravir	4	
Etravirine, Raltegravir	2	
**PI+INT**		**9**
Darunavir, Norvir, Raltegravir	4	
Darunavir, Norvir, Dolutegravir	3	
Darunavir/Cobicistat, Dolutegravir	1	
Saquinavir, Norvir, Raltegravir	1	
**NRTI+NNRTI+INT**		**4**
Emtricitabine, Nevirapine, Raltegravir	2	
Abacavir/Lamivudine, Nevirapine, Raltegravir	1	
Emtricitabine/Tenofovir, Etravirine, Raltegravir	1	
**NRTI+PI+INT**		**3**
Emtricitabine/Tenofovir Alafenamide, Darunavir/Cobicistat, Dolutegravir	2	
Emtricitabine/Tenofovir, Atazanavir, Raltegravir	1	
**PI mono**		**2**
Darunavir, Norvir	2	
**PI+NNRTI+INT**		**2**
Darunavir, Norvir, Nevirapine, Raltegravir	1	
Darunavir, Norvir, Nevirapine, Dolutegravir	1	
**INT+ENT**		**1**
Dolutegravir, Maraviroc	1	
**NNRTI+ENT**		**1**
Nevirapine, Maraviroc	1	
**NNRTI+INT+ENT**		**1**
Etravirine, Raltegravir, Maraviroc, Norvir	1	
**NRTI+PI+INT+ENT**		**1**
Tenofovir, Darunavir, Norvir, Raltegravir, Maraviroc	1	
**NRTI+PI+NNRTI+INT**		**1**
Tenofovir, Darunavir, Norvir, Etravirine, Raltegravir	1	
**STR-group (n = 56)**		
**NRTI+INT**		**45**
Emtricitabine/tenofovir alafenamide/elvitegravir/cobicistat	12	
Emtricitabine/tenofovir/elvitegravir/cobicistat	2	
Dolutegravir/abacarvir/lamivudine	31	
**PI mono**		**11**
Darunavir/cobicistat	11	

Baseline PROs were not significant different between the two groups and are shown in Table 4.

**Table 4:** Baseline patient-reported outcomes in both study arms.

	STR (n = 56)		MTR (n = 132)		p-value
	Median	IQR	Median	IQR	
HRQoL					
• EuroQol-6D utility score	0.7641	0.6607–1	0.7641 ^5^	0.7444–1	0.325
• EuroQol VAS	79	70–85	80 ^6^	70–85.75	0.348
• MOS-HIV PHS	53.61 ^1^	47.32–57.90	52.35 ^4^	47.81–56.75	0.467
• MOS-HIV MHS	52.55 ^1^	45.80–59.16	49.91 ^4^	43.65–56.84	0.191
Treatment satisfaction	55 ^3^	50–60	56 ^4^	50–59	0.676
Presence of neurocognitive complaints	26 ^2^	46.4	67	50.8	0.834
Adherence					
• CASE Adherence Index sum score	15	13–16	15 ^4^	14–16	0.247
• VAS	99.5	95–100	100	98–100	0.132
HIV symptoms	4 ^1^	1–7	4	2–6	0.670
Depressive symptoms (0–20) (n = 187)	6 ^1^	1–13	8 ^4^	3–14	0.205

1: n = 55.

2: n = 53.

3: n = 48.

4: n = 131.

5: n = 129.

6: n = 128.

### HRQoL

**Fig 1:**
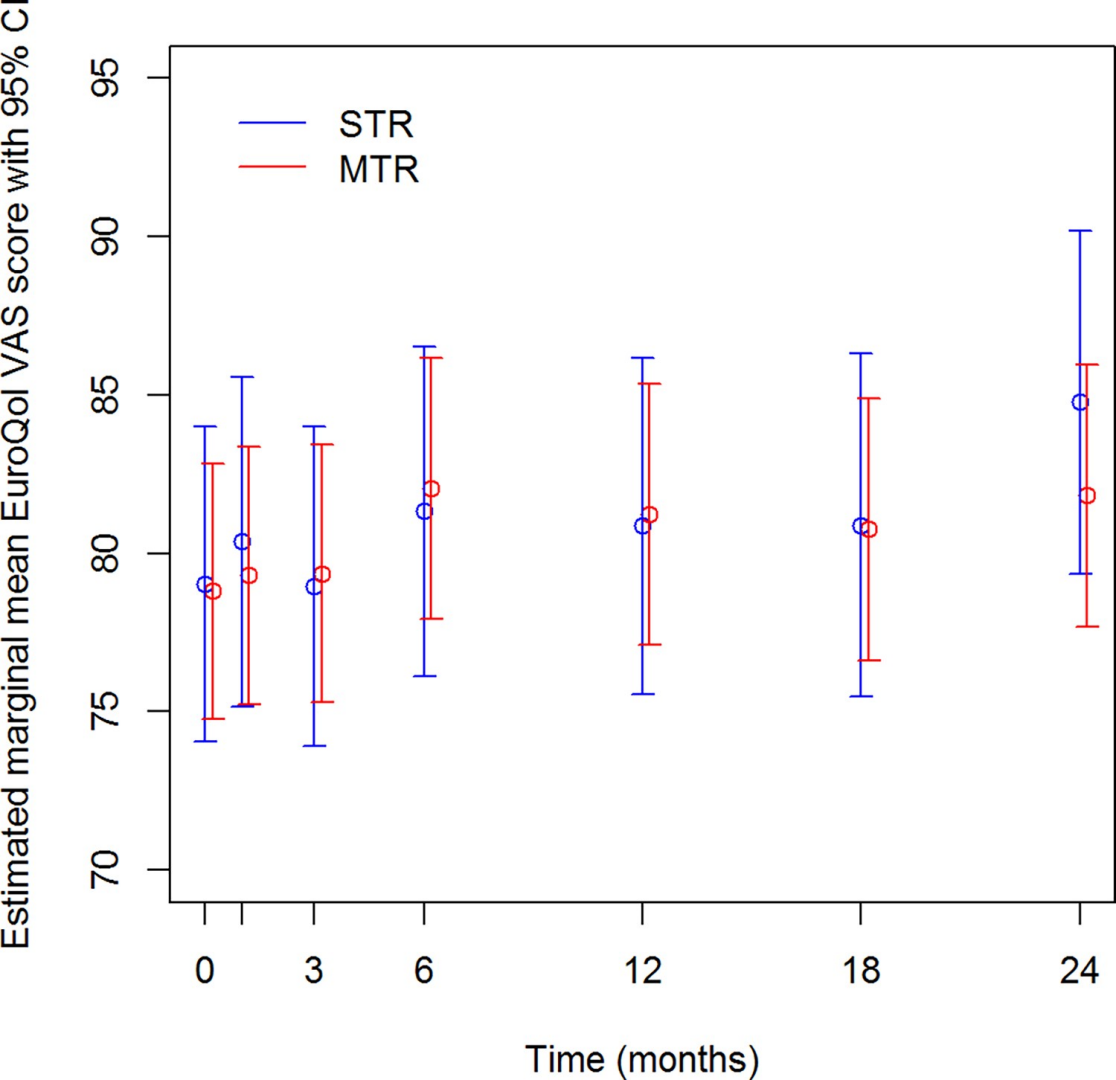
EuroQol VAS-score over time.

The estimated mean EuroQol utility score in both groups varied from 0.75 to 0.84, with no differences between the groups over time. The same was true for the VAS scale, with estimated mean scores from 78.8 to 84.8. The VAS-score increased for both groups: at T6, the score had increased by 5.73 in the STR-group (p = 0.004) and by 3.02 in the MTR-group as compared to baseline (p = 0.008) (Fig 1). Estimated physical and mental health scores of the MOS-HIV varied from 51.5 to 52.9 and from 50.3 to 51.4, respectively, and showed no differences over time or between groups.

### Treatment satisfaction

**Fig 2:**
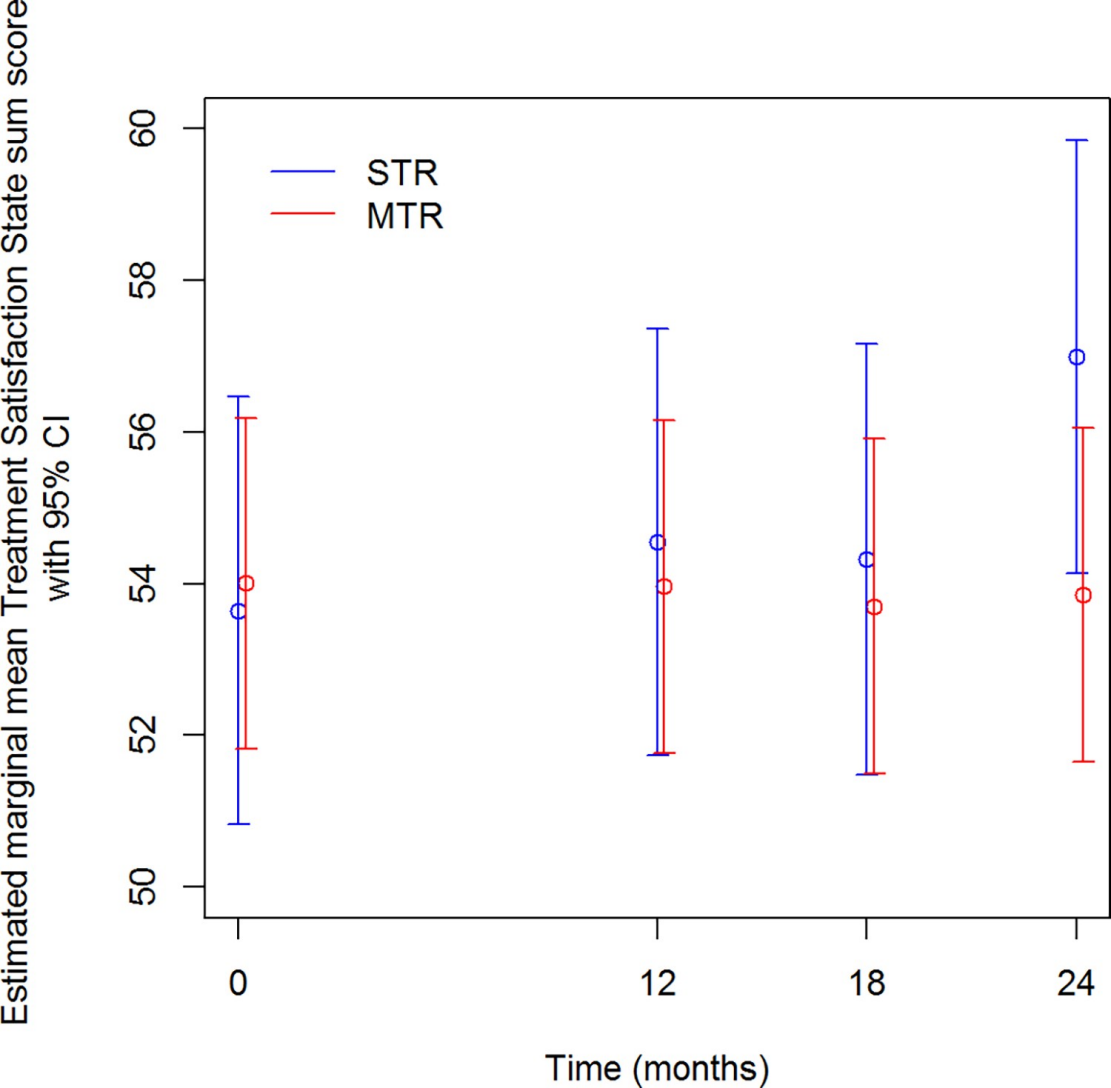
HIV treatment satisfaction state score over time.

The ‘HIV Treatment Satisfaction Questionnaire–change’ was included at T3 in the STR-group. For the following time points, the ‘state’ version was completed. Therefore, only T0-4-5-6 ‘state’ measures could be compared between groups and were included in the mixed model for treatment satisfaction. The estimated mean difference between T0 and T6 state scores between groups was significantly different: the score in the STR-group increased by 3.3, the score in the MTR-group decreased by 0.2 over time (p = 0.003) (Fig 2 and Table 5). This difference in treatment satisfaction was due to a higher score on the lifestyle/ease subscale among the STR-group: at T6 the median subscore was 29.5/30 (IQR 27–30) vs. 27/30 (IQR 24–30) in the MTR-group (p = 0.010). A high median HIVTSQ-change score was registered, already six months post-switch: 24 on a scale from -30 (less satisfied) to +30 (more satisfied).

**Table 5:** Estimated mean HIV treatment satisfaction state scores.

Estimates							
Time	Regimen	Mean	Std. Error	Df	95% Confidence Interval		
					Lower Bound	Upper Bound	Sig
T0	STR	53,645	1,431	191,288	50,823	56,467	,786
	MTR	54,004	1,103	154,743	51,825	56,183	
T4	STR	54,544	1,425	189,485	51,733	57,356	,659
	MTR	53,960	1,108	157,041	51,773	56,148	
T5	STR	54,325	1,439	195,137	51,487	57,163	,644
	MTR	53,703	1,120	163,218	51,492	55,914	
T6	STR	56,990	1,450	199,493	54,130	59,849	,021
	MTR	53,852	1,112	159,538	51,655	56,048	

### Neurocognitive complaints

**Fig 3:**
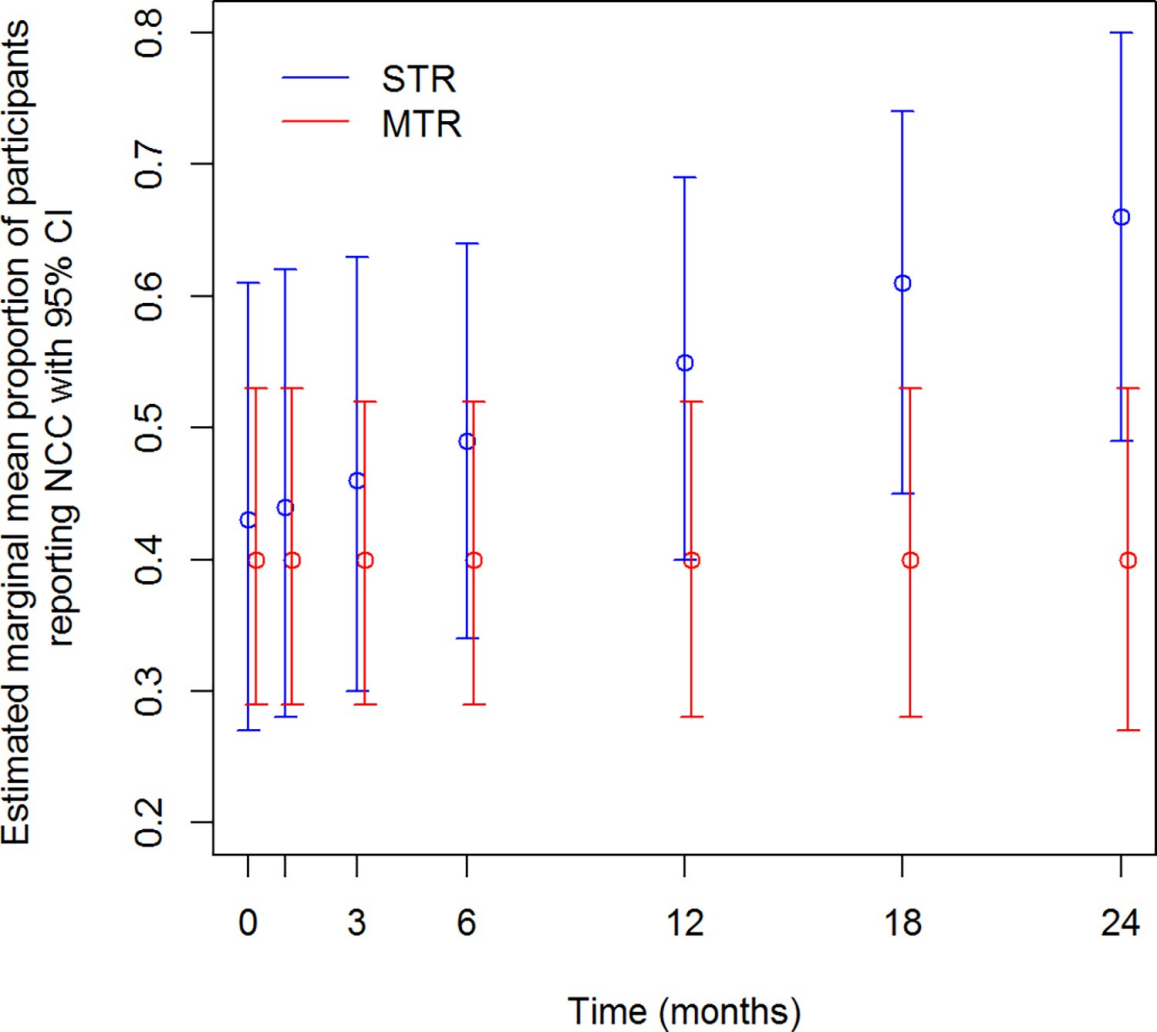
Proportion of participants reporting neurocognitive complaints over time.

NCC were more present in the STR-group: per month, the odds of having NCC increased with 4.1% among the STR-group as compared to the MTR-group (p = 0.035). At T6, participants in the STR-group had an significant higher odds on NCC, namely 3.005 as compared to the MTR-group (p = 0.002) (Fig 3 and Table 6).

**Table 6:** Estimated mean proportion of participants reporting neurocognitive complaints over time.

	Estimates					
	Regimen	Mean	Std. Error	95% Wald Confidence Interval		
Time				Lower	Upper	Sig
0	STR	,43	,092	,27	,61	,711
	MTR	,40	,063	,29	,53	
1	STR	,44	,090	,28	,62	,612
	MTR	,40	,062	,29	,53	
2	STR	,46	,086	,30	,63	,420
	MTR	,40	,062	,29	,52	0
3	STR	,49	,081	,34	,64	,188
	MTR	,40	,061	,29	,52	
4	STR	,55	,074	,40	,69	,018
	MTR	,40	,062	,28	,52	
5	STR	,61	,075	,45	,74	,003
	MTR	,40	,064	,28	,53	
6	STR	,66	,081	,49	,80	,002
	MTR	,40	,067	,27	,53	

### Other: Adherence, HIV symptoms and depressive symptoms

Analyses for the other PROs demonstrated no statistically significant differences among STR- and MTR-users. Adherence was high, and remained high with estimated VAS-scores between 92 and 95 in both groups. The estimated number of HIV symptoms was low in both groups (4/20) and reported symptoms (observed data) showed the same frequency pattern: fatigue (STR 60.7%, MTR 62.2%), sleep problems (STR 41.9%, MTR 40.1%) and trouble of remembering things (STR 40.2%, MTR 37.8%) were the top three. The estimated number of depressive symptoms remained in the ‘minimal’ range (between 6 to 9 on a maximum of 63) during the study and among the groups. There was a trend towards more depressive symptoms in the STR-group with an estimated mean increase of 0.10 symptoms per month as compared to the MTR-group (p = 0.075).

### Sensitivity analyses

Because of the relatively large amount of missing data, which were considered to be ‘missing not at random’ (MNAR), sensitivity analyses were performed [44]. The models changed substantially, with favorable outcomes in the group where outcomes were ‘improved’ by filling up the missing data by good outcomes and unfavorable outcomes for the other group. This urges towards caution with regard to the interpretation of our results. Sensitivity analyses can be found in S3 File.

## Discussion

This observational study compared patient-reported outcomes over time between therapy-experienced patients who switched to an STR and therapy-experienced patients who remained on their MTR.

Concerning HRQoL, no differences between the groups were observed on the four HRQoL scores used in this study. This corresponds to the results of Arribas et al. and Costa et al. [13, 14]. Hodder et al., in contrast, found a higher 48-week PHS-score (SF-36) in the STR-group, however the clinical relevance of the small difference was unclear [45]. HIV symptoms and depressive symptoms remained low and comparable among both groups in our study.

There was no evolution in adherence after switching from a MTR to an STR and adherence did not change in the stable MTR-group neither. Indeed, baseline adherence was already high and this persisted in both groups, comparable to clinical trials reported by Dejesus et al. and Hodder et al. on EFV/FTC/TDF versus MTRs [45, 46], and by Arribas et al. on EVG/C/FTC/TDF versus MTRs [13].

Treatment satisfaction did show a significant increase in the STR-group and at six months post-switch, 28/33 (84.8%) participants preferred their STR over their previous MTR. This confirms previous research, as more than 90% of PLHIV preferred taking their STR over their prior (MTR) regimen in the study of Hodder et al. [45] and six-months treatment satisfaction measures of STR-users were clearly higher in the trial of Arribas et al. [13].

With regards to neurocognitive complaints, there appeared to be less favorable outcomes in the STR-group. They were more likely to report NCC, and especially memory and attention problems were more present among the STR-group over time. We compared the results on the screening instrument with both the MOS-HIV Cognitive functioning subscale and the Cognitive item of the EuroQol-6D and the screening instrument correlated well with both additional measures (Mann-Whitney U p<0.001 and Pearson Chi-Square p<0.001). We hypothesized that the predominant use of DTG/ABC/3TC in the STR-group (31/56) may have influenced the analysis, as DTG exposure is known to be associated with neurocognitive impairment [47]. Therefore, we repeated the analysis with the data from the DTG/ABC/3TC users versus MTRs, but this showed no increased odds ratio on NCC as compared to the whole STR-group. However, we think that our screening instrument, consisting of three yes-no questions, is too limited to really examine this thoroughly. In general, however, many patients reported neurocognitive complaints and those problems may stay unnoticed if not explicitly asked for. Future clinical trials and studies on PROs should include cognitive functioning as an important outcome in PLHIV, preferable by performing in depth cognitive assessment.

The question on whether STRs could be replaced by (multitablet) generic regimens, remains open to discussion. Among the PROs that were collected in this study, only treatment satisfaction was higher among the STR-group and this was not translated into better adherence or better HRQoL over time. Thus, it seems that MTRs are not associated with inferior PROs and the use of generic ART could be promoted. The European AIDS Clinical Society guidelines call for such an evolution: “An increasing number of generic HIV drugs are now available, and their use can lead to large cost savings. The use of generic forms of drugs included in recommended regimens should therefore be encouraged, even if single tablet regimens are not used, as recent studies have shown similar virologic outcomes in ART-naïve PLWH receiving either a single pill or two pills qd” [48]. Per contra, a time span of two years may be too short to evaluate long-term effects of STRs versus MTRs in terms of, for example, persistence to ART. Studies indicate better persistence among STR-users [4, 5, 9, 10]. The higher treatment satisfaction among people taking an STR may indeed act as a trigger to better continued motivation to take lifelong medication [12]. This results in stable virological control, associated good health outcomes, combined with treatment-as-prevention benefits (i.e. this person can not transmit the virus to others), which signify large health and cost profits. Future research should therefore follow patients over a longer time span and address treatment persistence.

### Limitations

A number of issues concerning this observational study require further consideration. First, the STR- and MTR-group diverged with regard to socio-demographics and antiretroviral agents and the sample size was limited. The heterogeneity of the groups and the small sample size hampered possible subanalyses. Second, the reasons for switch were not asked for and participants may have had differences that were not recorded in the study. Moreover, the drop-out (especially in the STR-group) could have influenced our results. For instance, the participants who had only one measure (and thus not included in the models) had a lower adherence at baseline. Sensitivity analyses showed that the models were prone to changes when missing data were adjusted and this compels us to interpret our results cautiously. However, we believe that this comprehensive ‘real life’ study can add to the limited research on PROs between STRs and MTRs.

## Conclusion

Patient-reported outcomes among both STRs and MTRs were favorable in this study. HRQoL and adherence were high and remained high in both study arms, which supports a possible reintroduction of generic ART components as MTRs. Moreover, patients on an STR were more likely to report neurocognitive complaints. The impact of neurocognitive problems can not be underestimated, both at patient- and public health level. On the other hand, it should be noted that patients on an STR were more satisfied about their treatment. Taking into account PLHIV’s need for continued ART to control their HIV infection, treatment satisfaction could be seen as a current predictor for long-term adherence and persistence. In other words, the higher costs of STRs could be justified in light of future patient- and public health gains. Future studies should investigate long-term adherence among STR-users and neurocognitive problems associated with different ART regimens.

## Supporting information

S1 File Detailed information on methods to build mixed models and to perform sensitivity analyses. (DOCX)

S2 File Estimates of fixed effects, estimates and graphs of the models. (DOCX)

S3 File Results of sensitivity analyses. (DOCX)
