# Supplementary material for: Patient-reported outcomes among people living with HIV on single- versus multi-tablet regimens: Data from a real-life setting
Source: PLoS One. 2022 Jan 13;17(1):e0262533. doi: 10.1371/journal.pone.0262533 (PMC8758085; doi:10.1371/journal.pone.0262533)
Supplement: S1 File — (DOCX) [file pone.0262533.s001.docx]

# Additional file

# ‘Making of’ mixed models

1) Model with random intercept was built (RI) (unstructured covariance matrix)

2) Model with random intercept and random slope was built (RIRS) (unstructured covariance matrix)

3) These two models were compared by means of LRT (Restricted/residual maximum likelihood approach, REML, to compare models with different random effects part)

Critical value: 1/2χ²_0.95,1_+1/2χ²_0.95,2_=1/2 * 3.84+1/2 * 5.99=4.92

4.92 is the critical value of the LRT statistic for testing the need of a correlated random slope at the 5% significance level (approximate distribution is a mixture of two χ² distributions).
If LRT > 4.92, RIRS model fits best (reject RI model)

4) If RIRS fits better, a model with diagonal covariance structure was built. This model was compared to the ‘full’ model with unstructured covariance matrix (trough REML).

Number of parameters in random effects parts of unstructured model: 3

Number of parameters in random effects parts of diagonal model: 2

Df = 1

Critical value : 3.84

If LRT > 3.84: we reject the model with diagonal covariance matrix in favor of the full model with unstructured covariance matrix for the random effects at the 5% significance level (then the p-value is ≤ 0.05).
If LRT ≤ 3.84: no significant worse fit of the diagonal model, we continue with the diagonal model

5) If RIRS fits better, model with time continuous was built. This model was compared to the ‘full’ model with time categorical (standard likelihood approach, ML, to compare models with different fixed effect parts)

Df_model 1_ 20 & df_model 2_ 10, so df = 20 – 10 = 10

Critical value: 18.307

If LRT > 18.307, we reject the model with time continuous in favor of the full model with time categorical (then the p-value is ≤ 0.05).
If LRT ≤ 18.307: no significant worse fit of the continuous model, we continue with the model with time continuous.

# Sensitivity analyses

1. Lost-to-follow-up data were filled, i.e. missing data from one timepoint until T6. In case of a ‘intermediate’ missing value, this was left as a missing value.
2. Means and standard deviations of the variables from the original database (no selection) were determined.
3. Two databases were created:

A ‘best-worst’ database: STR-group missings were ‘good’, MTR-group missings were ‘bad’
A ‘worst-best’ database: STR-group missings were ‘bad’, MTR-group missings were ‘good’

1. Mean ± 1 standard deviation values were used to fill in the lost-to-follow-up data, according to the desired result.
   For example: EuroQol utility missings from STR-group in the best-worst case database were filled with mean+1SD (thus a ‘good’ score, a high utility). HIV-symptoms missings from the STR-group in the best-worst case database were filled with mean-1SD (thus a ‘good’ score, low number of symptoms).
