# Supplementary material for: Patient-reported outcomes among people living with HIV on single- versus multi-tablet regimens: Data from a real-life setting
Source: PLoS One. 2022 Jan 13;17(1):e0262533. doi: 10.1371/journal.pone.0262533 (PMC8758085; doi:10.1371/journal.pone.0262533)

# **Mixed models for manuscript**

7 timepoints, only data from participants with 2 or more present values were included in the models.

## Symptoms (HIV Symptoms Index, range 0-20, more symptoms = worse outcome)

Model with random intercept and time categorical.
-2Restricted Log Likelihood: 4582.485
Akaike’s Information Criterion (AIC): 4586.485
71.7% of data were available (943/1316).
164/188 (87.2%) people had 2 or more present values, if they would have all 7 values, there would be 1148 values.
However, only 82.1 % of the 1148 values were present (943/1148) and could be included in the model.

| **Symptoms: Estimates of fixed effects** | | | | | | | |
| --- | --- | --- | --- | --- | --- | --- | --- |
| Parameter | Estimate | Std. Error | Df | t | Sig. | 95% Confidence Interval | |
|  |  |  |  |  |  | Lower Bound | Upper Bound |
| Intercept | 4,892028 | 0,458980 | 342,082 | 10,658 | 0,000 | 3,989250 | 5,794806 |
| Baseline | -0,258608 | 0,385805 | 736,656 | -0,670 | 0,503 | -1,016017 | 0,498801 |
| Month 1 | -0,544482 | 0,395755 | 734,834 | -1,376 | 0,169 | -1,321427 | 0,232463 |
| Month 3 | 0,205000 | 0,391695 | 734,181 | 0,523 | 0,601 | -0,563975 | 0,973975 |
| Month 6 | -0,516146 | 0,406066 | 731,277 | -1,271 | 0,204 | -1,313339 | 0,281048 |
| Month 12 | -0,314077 | 0,403237 | 729,191 | -0,779 | 0,436 | -1,105721 | 0,477567 |
| Month 18 | -0,225721 | 0,411042 | 729,936 | -0,549 | 0,583 | -1,032686 | 0,581245 |
| Month 24 | 0 | 0 |  |  |  |  |  |
| STR group | -0,453285 | 0,858105 | 434,804 | -0,528 | 0,598 | -2,139834 | 1,233264 |
| MTR group | 0 | 0 |  |  |  |  |  |
| Baseline * STR group | 0,681133 | 0,781806 | 738,017 | 0,871 | 0,384 | -0,853696 | 2,215961 |
| Baseline * MTR group | 0 | 0 |  |  |  |  |  |
| Month 1 * STR group | 0,959154 | 0,828696 | 741,734 | 1,157 | 0,247 | -0,667714 | 2,586023 |
| Month 1 * MTR group | 0 | 0 |  |  |  |  |  |
| Month 3 * STR group | 0,217793 | 0,796744 | 736,558 | 0,273 | 0,785 | -1,346367 | 1,781953 |
| Month 3 * MTR group | 0 | 0 |  |  |  |  |  |
| Month 6 * STR group | 0,167399 | 0,810710 | 731,194 | 0,206 | 0,836 | -1,424199 | 1,758997 |
| Month 6 * MTR group | 0 | 0 |  |  |  |  |  |
| Month 12 * STR group | 0,638773 | 0,829918 | 731,594 | 0,770 | 0,442 | -0,990532 | 2,268078 |
| Month 12 * MTR group | 0 | 0 |  |  |  |  |  |
| Month 18 * STR group | 1,284991 | 0,847792 | 730,507 | 1,516 | 0,130 | -0,379409 | 2,949392 |
| Month 18 * MTR group | 0 | 0 |  |  |  |  |  |
| Month 24 * STR group | 0 | 0 |  |  |  |  |  |
| Month 24 * MTR group | 0 | 0 |  |  |  |  |  |
| Women | 0,566470 | 0,964959 | 152,019 | 0,587 | 0,558 | -1,339992 | 2,472933 |
| Men | 0 | 0 |  |  |  |  |  |
| Non-Caucasian | -1,451984 | 1,209369 | 159,222 | -1,201 | 0,232 | -3,840459 | 0,936490 |
| Caucasian | 0 | 0 |  |  |  |  |  |
| Heterosexual | -0,406146 | 0,765569 | 152,422 | -0,531 | 0,597 | -1,918642 | 1,106350 |
| Homosexual | 0 | 0 |  |  |  |  |  |

Intercept: At 2y, MTR, Caucasian, homosexual men have 4.89 symptoms (p<0.001).

Time: Within the MTR-group, there are no differences over time as compared to 2y. *A second model (in which 2y+STR are the reference groups) does not show differences within the STR-group over time as compared to 2y. A third and fourth model (in which baseline+MTR or baseline+STR are the reference groups) do not show differences in both groups as compared to baseline.*

Stable: At 2y, the STR-group has 0.45 symptoms less than the MTR-group. This difference is not significant (p=0.598). *A second model shows that at baseline, the STR-group has 0.23 symptoms more than the MTR-group, this difference is also non-significant (p=0.767)*

Interaction: The estimated mean difference in symptoms between baseline and 2y between the groups is 0.68. This difference is not significant (p=0.384)
- Symptoms in the STR-group decreased with 0.42, symptoms in the MTR-group increased with 0.26 over time
- The STR-group had 0.23 symptoms more at baseline and 0.453 less at 2y.

Differences according to gender, ethnicity and sexual orientation are not significant.

| **Symptoms: Estimates** | | | | | | | |  |
| --- | --- | --- | --- | --- | --- | --- | --- | --- |
| Time | Group | | Mean | Std. Error | df | 95% Confidence Interval | |  |
|  |  |  |  |  |  | Lower Bound | Upper Bound | Sig |
| Baseline | | STR | 4,215 | 0,784 | 252,454 | 2,671 | 5,759 | ,767 |
|  |  | MTR | 3,988 | 0,615 | 208,177 | 2,776 | 5,199 | ,767 |
| Month 1 | | STR | 4,208 | 0,824 | 299,387 | 2,587 | 5,828 | ,536 |
|  |  | MTR | 3,702 | 0,624 | 219,717 | 2,472 | 4,931 | ,536 |
| Month 3 | | STR | 4,216 | 0,799 | 269,555 | 2,642 | 5,790 | ,765 |
|  |  | MTR | 4,451 | 0,621 | 216,098 | 3,227 | 5,676 | ,765 |
| Month 6 | | STR | 3,444 | 0,821 | 295,401 | 1,828 | 5,061 | ,726 |
|  |  | MTR | 3,730 | 0,635 | 233,072 | 2,480 | 4,980 | ,726 |
| Month 12 | | STR | 4,118 | 0,842 | 320,650 | 2,461 | 5,774 | ,824 |
|  |  | MTR | 3,932 | 0,634 | 232,377 | 2,683 | 5,181 | ,824 |
| Month 18 | | STR | 4,852 | 0,862 | 345,922 | 3,157 | 6,547 | ,334 |
|  |  | MTR | 4,020 | 0,639 | 239,075 | 2,762 | 5,279 | ,334 |
| Month 24 | | STR | 3,793 | 0,862 | 345,871 | 2,098 | 5,488 | ,598 |
|  |  | MTR | 4,246 | 0,638 | 237,778 | 2,989 | 5,503 | ,598 |

**Symptoms: Graph**


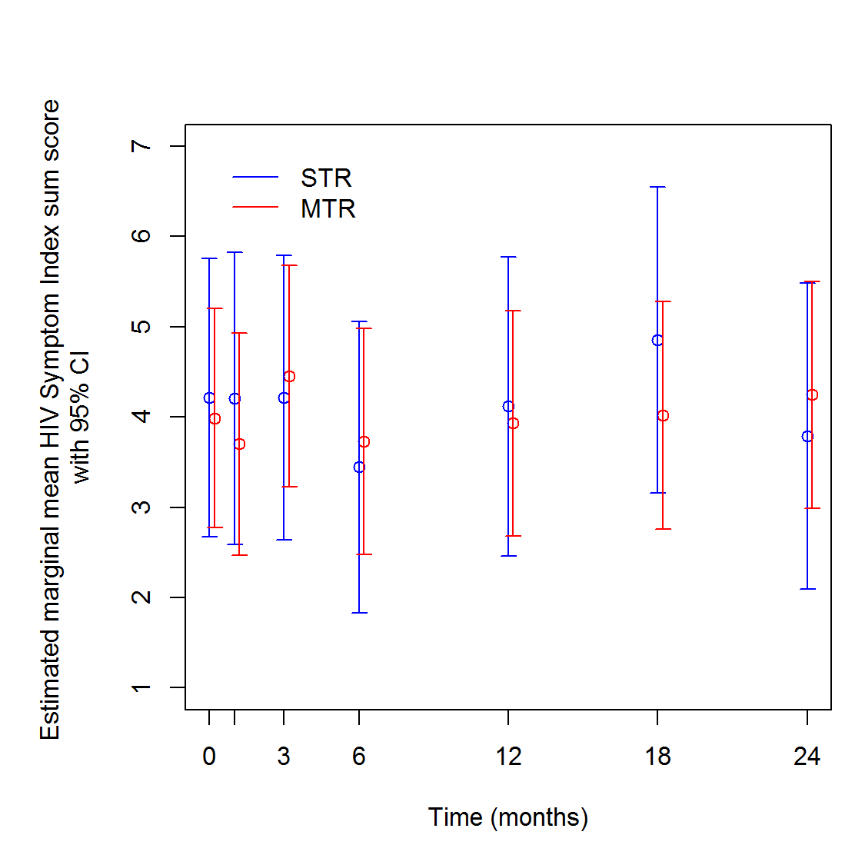


## Depressive symptoms (Beck Depression Inventory II, range 0-63, higher score = worse outcome)

Model with random intercept, random slope, diagonal covariance structure and time continuous.
-2Restricted Log Likelihood: 5623.020
AIC: 5629.020
71.6% of data were available (942/1316).
165/188 (87.8%) people had 2 or more present values, if they would have all 7 values, there would be 1155 values.
However, only 81.6% of these values were present (942/1155) and could be included in the model.

| **BDI: Estimates of Fixed Effects** | | | | | | | |
| --- | --- | --- | --- | --- | --- | --- | --- |
| Parameter | Estimate | Std. Error | df | T | Sig. | 95% Confidence Interval | |
|  |  |  |  |  |  | Lower Bound | Upper Bound |
| Intercept | 8,133132 | ,974865 | 175,907 | 8,343 | ,000 | 6,209195 | 10,057069 |
| Time continuous | -,035907 | ,025675 | 162,200 | -1,398 | ,164 | -,086607 | ,014794 |
| STR group | 2,861205 | 1,739473 | 196,700 | 1,645 | ,102 | -,569206 | 6,291616 |
| MTR group | 0 | 0 | . | . | . | . | . |
| STR * Time continuous | ,094743 | ,052893 | 177,448 | 1,791 | ,075 | -,009637 | ,199123 |
| MTR * Time continuous | 0 | 0 | . | . | . | . | . |
| Women | 1,092267 | 2,447878 | 146,313 | ,446 | ,656 | -3,745499 | 5,930034 |
| Men | 0 | 0 | . | . | . | . | . |
| Non-Caucasian | -4,438201 | 3,071631 | 150,989 | -1,445 | ,151 | -10,507129 | 1,630728 |
| Caucasian | 0 | 0 | . | . | . | . | . |
| Heterosexual | -,476645 | 1,941857 | 146,336 | -,245 | ,806 | -4,314352 | 3,361063 |
| Homosexual | 0 | 0 | . | . | . | . | . |

Intercept: At 2y MTR-, Caucasian, homosexual men have 8.13 depressive symptoms (p<0.001).

Time: Per month, the number of depressive symptoms decreases by 0.036 in the stable, Causasian, homosexual group. This is not significant (p=0.164)

Stable: At 2y, the STR-group (men, Caucasian, homosexual) has 2.86 depressive symptoms more than the MTR-group. This difference is not significant (p=0.102)

Interaction: The estimated mean increase in BDI score per month is 0.095 higher under STR than under MTR (the 95% CI goes from 0.01 lower to 0.20 higher. This difference is not significant (p=0.075).

Differences according to gender, ethnicity and sexual orientation are not significant.

|  | **BDI: Estimates** | | | | | |  |
| --- | --- | --- | --- | --- | --- | --- | --- |
|  | Group | Mean | Std. Error | Df | 95% Confidence Interval | |  |
| Time |  |  |  |  | Lower Bound | Upper Bound | Sig |
| Baseline | STR | 7,671 | 1,842 | 157,342 | 4,033 | 11,309 | ,736 |
|  | MTR | 7,084 | 1,494 | 156,205 | 4,133 | 10,034 | ,736 |
| Month 1 | STR | 7,730 | 1,830 | 155,881 | 4,116 | 11,344 | ,692 |
|  | MTR | 7,048 | 1,489 | 155,232 | 4,107 | 9,988 | ,692 |
| Month 3 | STR | 7,847 | 1,808 | 153,301 | 4,276 | 11,419 | ,606 |
|  | MTR | 6,976 | 1,479 | 153,541 | 4,053 | 9,898 | ,606 |
| Month 6 | STR | 8,024 | 1,784 | 150,603 | 4,499 | 11,549 | ,485 |
|  | MTR | 6,868 | 1,469 | 151,733 | 3,966 | 9,770 | ,485 |
| Month 12 | STR | 8,377 | 1,768 | 151,215 | 4,884 | 11,870 | ,289 |
|  | MTR | 6,653 | 1,460 | 151,223 | 3,768 | 9,537 | ,289 |
| Month 18 | STR | 8,730 | 1,796 | 162,423 | 5,184 | 12,276 | ,167 |
|  | MTR | 6,437 | 1,467 | 155,546 | 3,539 | 9,335 | ,167 |
| Month 24 | STR | 9,083 | 1,864 | 186,400 | 5,405 | 12,761 | ,102 |
|  | MTR | 6,222 | 1,490 | 165,289 | 3,280 | 9,164 | ,102 |

**BDI: Graph**


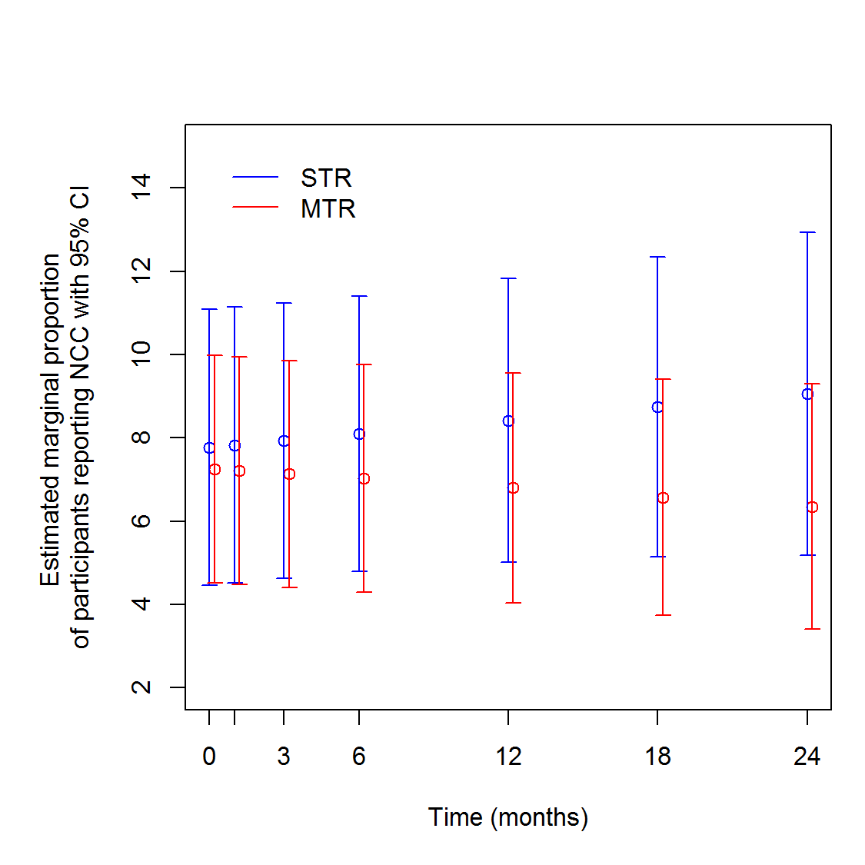


## Quality of life: EuroQol Utility (EuroQol 5D-3L, range 0-1, higher score = better outcome)

Model with random intercept, random slope, diagonal covariance structure and time categorical.
-2Restricted Log Likelihood: -812.069
AIC: -806.069
71.4% of data were available (940/1316).
164/188 (87.2%) people had 2 or more present values, if they would have all 7 values, there would be 1148 values.
However, only 81.9% of the values were present (940/1155) and could be included in the model.

| **EuroQol utility: Estimates of Fixed Effects** | | | | | | | |
| --- | --- | --- | --- | --- | --- | --- | --- |
| Parameter | Estimate | Std. Error | Df | T | Sig. | 95% Confidence Interval | |
|  |  |  |  |  |  | Lower Bound | Upper Bound |
| Intercept | ,854735 | ,023276 | 241,790 | 36,721 | ,000 | ,808885 | ,900586 |
| Baseline | -,016917 | ,019541 | 245,677 | -,866 | ,388 | -,055406 | ,021573 |
| Month 1 | -,018310 | ,019694 | 281,963 | -,930 | ,353 | -,057075 | ,020455 |
| Month 3 | -,034630 | ,019225 | 335,919 | -1,801 | ,073 | -,072447 | ,003188 |
| Month 6 | -,024898 | ,019251 | 489,880 | -1,293 | ,196 | -,062723 | ,012926 |
| Month 12 | -,055059 | ,018362 | 702,312 | -2,999 | ,003 | -,091110 | -,019008 |
| Month 18 | -,002184 | ,018199 | 691,346 | -,120 | ,905 | -,037916 | ,033547 |
| Month 24 | 0^b^ | 0 | . | . | . | . | . |
| STR group | -,029879 | ,043874 | 243,568 | -,681 | ,496 | -,116299 | ,056541 |
| MTR group | 0^b^ | 0 | . | . | . | . | . |
| Baseline * STR group | -,018030 | ,039432 | 240,781 | -,457 | ,648 | -,095705 | ,059646 |
| Baseline * MTR group | 0^b^ | 0 | . | . | . | . | . |
| Month 1 * STR group | ,005488 | ,041091 | 296,619 | ,134 | ,894 | -,075379 | ,086354 |
| Month 1 * MTR group | 0^b^ | 0 | . | . | . | . | . |
| Month 3 * STR group | ,032978 | ,039068 | 324,871 | ,844 | ,399 | -,043881 | ,109836 |
| Month 3 * MTR group | 0^b^ | 0 | . | . | . | . | . |
| Month 6 * STR group | -,015176 | ,038930 | 458,658 | -,390 | ,697 | -,091680 | ,061328 |
| Month 6 * MTR group | 0^b^ | 0 | . | . | . | . | . |
| Month 12 * STR group | -,009451 | ,037926 | 693,246 | -,249 | ,803 | -,083914 | ,065011 |
| Month 12 * MTR group | 0^b^ | 0 | . | . | . | . | . |
| Month 18 * STR group | -,030486 | ,037603 | 688,453 | -,811 | ,418 | -,104316 | ,043344 |
| Month 18 * MTR group | 0^b^ | 0 | . | . | . | . | . |
| Month 24 * STR group | 0^b^ | 0 | . | . | . | . | . |
| Month 24 * MTR group | 0^b^ | 0 | . | . | . | . | . |
| Women | -,076419 | ,046655 | 146,388 | -1,638 | ,104 | -,168623 | ,015785 |
| Men | 0^b^ | 0 | . | . | . | . | . |
| Non-Caucasian | ,034510 | ,056850 | 152,745 | ,607 | ,545 | -,077803 | ,146823 |
| Caucasian | 0^b^ | 0 | . | . | . | . | . |
| Heterosexual | ,016468 | ,036656 | 147,763 | ,449 | ,654 | -,055970 | ,088906 |
| Homosexual | 0^b^ | 0 | . | . | . | . | . |

Intercept:
At 2y, MTR, Caucasian, homosexual men have a utility of 0.8547 (p<0.001)

Time:
Within the MTR-group, there is no difference as compared to 2y at baseline, month 1, month 3, month 6 and month 18. At 1y, the utililty is 0.055 less than at 2y (p = 0.003).

*A second model (in which 2y+STR are the reference groups) shows a borderline significant different score within the STR-group at 1y: the utility is 0.064 less than at 2y (p=0.052).*

*A third model (in which baseline+MTR are the reference groups) shows a significantly different utility score within the MTR-group at 1y: the utility is 0.038 less as compared to baseline.*

*A fourth model (in which baseline+STR are the reference groups) does not show differences within the STR-group as compared to baseline.*

Stable:
At 2y, the STR-group’s utility (-0.030) is not significantly different as compared to the utility in the MTR-group (p=0.496). *A second model shows that at baseline, the STR-group’s utility (-0.048) is not significantly different as compared to the utility in the MTR-group (p=0.176)*

Interaction:
The estimated mean utility difference between baseline and 2y between the groups is 0.018. This difference is not significant (p=0.648)
- Utility in the STR-group increased with 0.035, utility in the MTR-group increased with 0.017 over time
- The STR-group had a 0.048 lower utility at baseline and 0.030 less at 2y.

Differences according to gender, ethnicity and sexual orientation are not significant.

| **EuroQol utility: Estimates** | | | | | | |  |
| --- | --- | --- | --- | --- | --- | --- | --- |
| Tijd | Group | Mean | Std. Error | Df | 95% Confidence Interval | | Sig |
|  |  |  |  |  | Lower Bound | Upper Bound |  |
| Baseline | STR | ,777 | ,035 | 225,407 | ,708 | ,847 | ,176 |
|  | MTR | ,825 | ,028 | 194,235 | ,769 | ,881 | ,176 |
| Month 1 | STR | ,799 | ,037 | 272,563 | ,726 | ,873 | ,515 |
|  | MTR | ,824 | ,029 | 202,090 | ,767 | ,880 | ,515 |
| Month 3 | STR | ,810 | ,036 | 240,399 | ,740 | ,881 | ,932 |
|  | MTR | ,807 | ,029 | 199,460 | ,751 | ,864 | ,932 |
| Month 6 | STR | ,772 | ,038 | 281,817 | ,698 | ,846 | ,233 |
|  | MTR | ,817 | ,029 | 213,922 | ,759 | ,875 | ,233 |
| Month 12 | STR | ,748 | ,039 | 311,357 | ,671 | ,824 | ,317 |
|  | MTR | ,787 | ,030 | 221,616 | ,729 | ,845 | ,317 |
| Month 18 | STR | ,779 | ,041 | 326,483 | ,699 | ,860 | ,149 |
|  | MTR | ,840 | ,030 | 233,020 | ,780 | ,899 | ,149 |
| Month 24 | STR | ,812 | ,043 | 265,384 | ,728 | ,896 | ,496 |
|  | MTR | ,842 | ,031 | 229,536 | ,781 | ,903 | ,496 |
|  | | | | | | |  |

**EuroQol Utility: Graph**


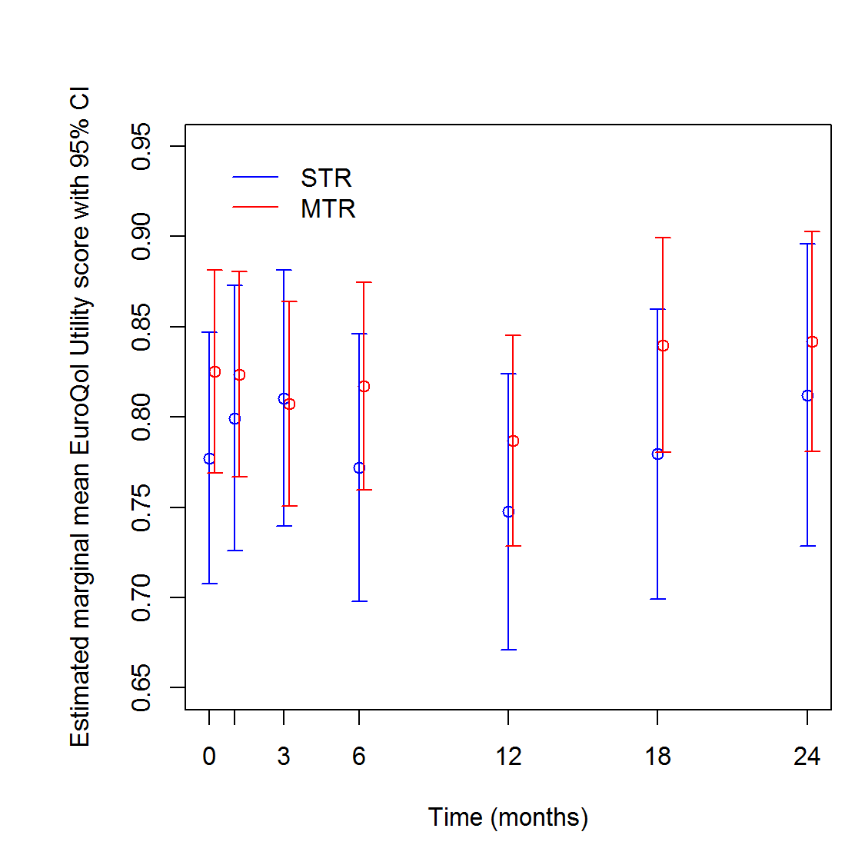


## Quality of Life: Visual Analogue Scale (EuroQol VAS, range 0-100, higher score = better outcome)

Model with random intercept and time categorical.
-2Restricted Log Likelihood: 6428.045
AIC: 6432.045
70.9% of data were available (933/1316).
163/188 (86.7%) people had 2 or more present values, if they would have all 7 values, there would be 1141 values.
However, only 81.8% of the values were present (933/1141) and could be included in the model.

| **EuroQol VAS: Estimates of Fixed Effects** | | | | | | | |
| --- | --- | --- | --- | --- | --- | --- | --- |
| Parameter | Estimate | Std. Error | df | t | Sig. | 95% Confidence Interval | |
|  |  |  |  |  |  | Lower Bound | Upper Bound |
| Intercept | 81,522569 | 1,507801 | 280,149 | 54,067 | ,000 | 78,554511 | 84,490628 |
| Baseline | -3,016135 | 1,137836 | 722,755 | -2,651 | ,008 | -5,249994 | -,782277 |
| Month 1 | -2,520493 | 1,161269 | 720,627 | -2,170 | ,030 | -4,800368 | -,240618 |
| Month 3 | -2,461669 | 1,150568 | 720,734 | -2,140 | ,033 | -4,720533 | -,202805 |
| Month 6 | ,224932 | 1,189722 | 718,335 | ,189 | ,850 | -2,110815 | 2,560679 |
| Month 12 | -,604115 | 1,187529 | 716,619 | -,509 | ,611 | -2,935568 | 1,727338 |
| Month 18 | -1,069076 | 1,195987 | 717,097 | -,894 | ,372 | -3,417130 | 1,278978 |
| Month 24 | 0^b^ | 0 | . | . | . | . | . |
| STR group | 2,941450 | 2,759992 | 349,220 | 1,066 | ,287 | -2,486847 | 8,369747 |
| MTR group | 0^b^ | 0 | . | . | . | . | . |
| Baseline * STR group | -2,717609 | 2,280513 | 724,018 | -1,192 | ,234 | -7,194817 | 1,759598 |
| Baseline * MTR group | 0^b^ | 0 | . | . | . | . | . |
| Month 1 * STR group | -1,888334 | 2,419704 | 726,033 | -,780 | ,435 | -6,638786 | 2,862118 |
| Month 1 * MTR group | 0^b^ | 0 | . | . | . | . | . |
| Month 3 * STR group | -3,348431 | 2,320626 | 722,982 | -1,443 | ,149 | -7,904401 | 1,207540 |
| Month 3 * MTR group | 0^b^ | 0 | . | . | . | . | . |
| Month 6 * STR group | -3,674689 | 2,364378 | 718,105 | -1,554 | ,121 | -8,316609 | ,967232 |
| Month 6 * MTR group | 0^b^ | 0 | . | . | . | . | . |
| Month 12 * STR group | -3,303950 | 2,423324 | 718,383 | -1,363 | ,173 | -8,061594 | 1,453694 |
| Month 12 * MTR group | 0^b^ | 0 | . | . | . | . | . |
| Month 18 * STR group | -2,814892 | 2,468143 | 717,543 | -1,140 | ,254 | -7,660537 | 2,030752 |
| Month 18 * MTR group | 0^b^ | 0 | . | . | . | . | . |
| Month 24 * STR group | 0^b^ | 0 | . | . | . | . | . |
| Month 24 * MTR group | 0^b^ | 0 | . | . | . | . | . |
| Women | -1,020857 | 3,361926 | 147,961 | -,304 | ,762 | -7,664448 | 5,622734 |
| Men | 0^b^ | 0 | . | . | . | . | . |
| Non-Caucasian | 7,074555 | 4,095637 | 154,036 | 1,727 | ,086 | -1,016312 | 15,165422 |
| Caucasian | 0^b^ | 0 | . | . | . | . | . |
| Heterosexual | -5,444471 | 2,641682 | 148,953 | -2,061 | ,041 | -10,664483 | -,224459 |
| Homosexual | 0^b^ | 0 | . | . | . | . | . |

Intercept:
At 2y, MTR, Caucasian, homosexual men have a score of 81.52 (p<0.001)

Time:
Within the MTR-group, there are differences as compared to 2y at baseline (-3.02, p = 0.008), month 1 (-2.52, p=0.030), month 3 (-2.46, p=0.033). At 1y, the utililty is 0.055 less than at 2y (p=0.003).

*A second model (in which 2y+STR-group are the reference groups) shows significant different scores within the STR-group at baseline (-5.73, p=0.004), month 1 (-4.41, p=0.038) and month 3 (-5.81, p=0.004) as compared to 2y.*

*A third model (in which baseline+MTR are the reference groups) shows significantly different scores within the MTR-group at month 3 (+3.24, p=0.004), 1y (+2.41, p=0.032) and 2y (+3.02, p=0.008) as compared to baseline.*

*A fourth model (in which baseline+STR are the reference groups) shows only a significant difference within the STR-group as compared to baseline at 2y (+5.73, p=0.004).*

Stable: At 2y, the score in the STR-group is 2.94 points higher but this difference is not significant (p=0.287). *A second model shows that at baseline, the STR-group’s score (+0.22) is not significantly different as compared to the score in the MTR-group (p=0.929)*

Interaction:
The estimated mean difference between baseline and 2y between the groups is 2.72. This difference is not significant (p=0.234)
- VAS-score in the STR-group increased by 5.73, the score in the MTR-group increased by 3.02 over time
- The STR-group had a 0.22 higher score at baseline and 2.94 higher at 2y.

Differences according to gender and ethnicity are not significant. We do see that at 2y, heterosexual (male, Caucasian, MTR) participants’ score is 5.44 lower than homosexual participants (p=0.041).

| **EuroQol VAS: Estimates** | | | | | | |  |
| --- | --- | --- | --- | --- | --- | --- | --- |
| Time | Group | Mean | Std. Error | Df | 95% Confidence Interval | |  |
|  |  |  |  |  | Lower Bound | Upper Bound | Sig |
| Baseline | STR | 79,035 | 2,527 | 220,940 | 74,055 | 84,015 | ,929 |
|  | MTR | 78,811 | 2,043 | 191,623 | 74,781 | 82,841 | ,929 |
| Month 1 | STR | 80,360 | 2,649 | 261,552 | 75,145 | 85,575 | ,691 |
|  | MTR | 79,307 | 2,064 | 198,776 | 75,237 | 83,376 | ,691 |
| Month 3 | STR | 78,959 | 2,565 | 232,994 | 73,906 | 84,011 | ,874 |
|  | MTR | 79,366 | 2,057 | 196,259 | 75,309 | 83,422 | ,874 |
| Month 6 | STR | 81,319 | 2,641 | 258,503 | 76,117 | 86,520 | ,782 |
|  | MTR | 82,052 | 2,088 | 207,298 | 77,936 | 86,168 | ,782 |
| Month 12 | STR | 80,861 | 2,696 | 277,834 | 75,553 | 86,169 | ,893 |
|  | MTR | 81,223 | 2,093 | 209,394 | 77,096 | 85,350 | ,893 |
| Month 18 | STR | 80,885 | 2,750 | 297,306 | 75,473 | 86,296 | ,963 |
|  | MTR | 80,758 | 2,098 | 211,060 | 76,623 | 84,893 | ,963 |
| Month 24 | STR | 84,769 | 2,750 | 297,273 | 79,357 | 90,180 | ,287 |
|  | MTR | 81,827 | 2,100 | 211,867 | 77,688 | 85,966 | ,287 |
|  | | | | | | |  |

**EuroQol VAS: Graph**


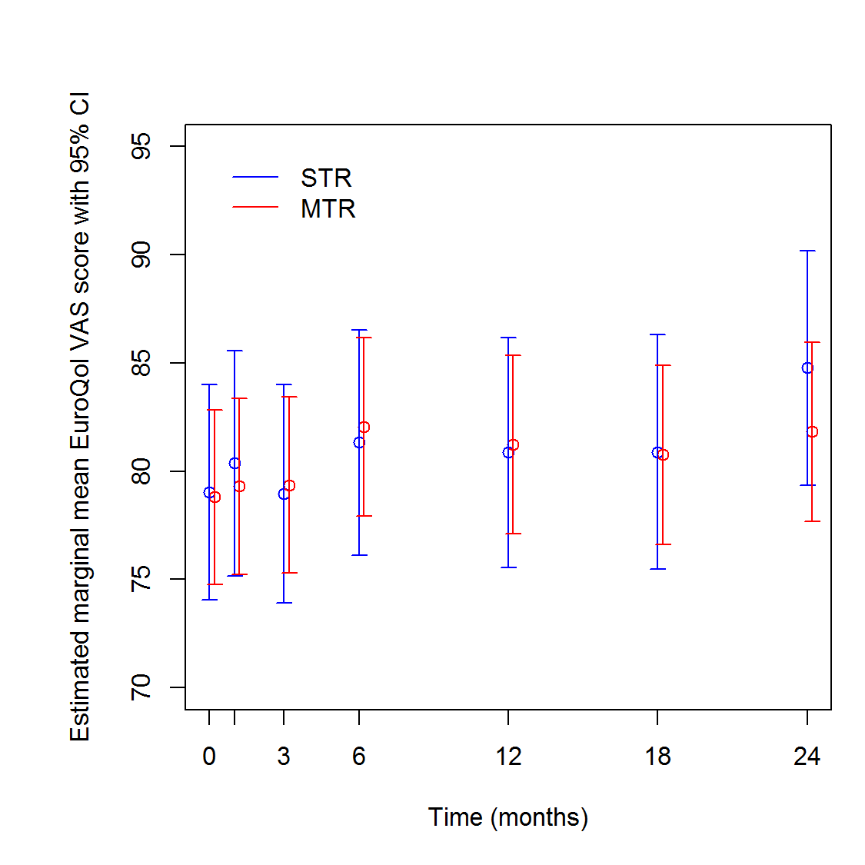


## Quality of Life: Physical health (Physical health score of MOS-HIV, standardized scale (T-score) with a mean of 50 and a standard deviation of 10 in the sample in which the summary scores were developed, higher score = better outcomes)

Model with random intercept, random slope, diagonal covariance structure and time continuous.
-2Restricted Log Likelihood: 5635.025
AIC: -5641.025
71,1% of data were available (936/1316).
165/188 (87.8%) people had 2 or more present values, if they would have all 7 values, there would be 1155 values.
However, only 81.0% of the values were present (936/1155) and could be included in the model.

| **MOS-HIV PHS: Estimates of Fixed Effects** | | | | | | | |
| --- | --- | --- | --- | --- | --- | --- | --- |
| Parameter | Estimate | Std. Error | df | t | Sig. | 95% Confidence Interval | |
|  |  |  |  |  |  | Lower Bound | Upper Bound |
| Intercept | 51,264324 | ,964574 | 178,275 | 53,147 | ,000 | 49,360873 | 53,167775 |
| Time continuous | ,010724 | ,025242 | 158,227 | ,425 | ,672 | -,039130 | ,060579 |
| STR group | -1,028584 | 1,731667 | 204,327 | -,594 | ,553 | -4,442812 | 2,385644 |
| MTR group | 0 | 0 | . | . | . | . | . |
| STR * Time continuous | -,070178 | ,052772 | 178,929 | -1,330 | ,185 | -,174314 | ,033958 |
| MTR * Time continuous | 0 | 0 | . | . | . | . | . |
| Women | -1,491490 | 2,469113 | 143,141 | -,604 | ,547 | -6,372125 | 3,389145 |
| Men | 0 | 0 | . | . | . | . | . |
| Non-Caucasian | 6,567601 | 3,083640 | 147,588 | 2,130 | ,035 | ,473812 | 12,661391 |
| Caucasian | 0 | 0 | . | . | . | . | . |
| Heterosexual | -2,605617 | 1,921084 | 144,418 | -1,356 | ,177 | -6,402691 | 1,191458 |
| Homosexual | 0 | 0 | . | . | . | . | . |

Intercept: At 2y, MTR-, Caucasian, homosexual men have a PHS-score of 51.26 (p<0.001).

Time: Per month, PHS increases by 0.01 in the MTR-group. This increase is not significant (p=0.672)

Stable: At 2y, the STR-group has a PHS-score of 1.03 less than the MTR group. This difference is not significant (p=0.553). *A second model (in which baseline is the reference category), the PHS-score in the STR-group at baseline is 0.71 lower than the MTR-group, this difference is not significant (0.651).*

Interaction: The estimated mean difference in PHS score per month is 0.07 lower in the STR-group than in the MTR-group (the 95% CI goes from 0.17 lower to 0.03 higher. This difference is not significant (p=0.185).

Differences according to gender and sexual orientation are not significant.
Non-Caucasian homosexual males have a 6.57 points higher PHS-score than Caucasian homosexual males at 2y (p=0.035).

|  | **MOS-HIV PHS: Estimates** | | | | | |  |
| --- | --- | --- | --- | --- | --- | --- | --- |
|  | Group | Mean | Std. Error | df | 95% Confidence Interval | |  |
| Time |  |  |  |  | Lower Bound | Upper Bound | Sig |
| Baseline | STR | 52,898 | 1,788 | 155,501 | 49,366 | 56,430 | ,699 |
|  | MTR | 52,242 | 1,454 | 154,138 | 49,370 | 55,114 | ,699 |
| Month 1 | STR | 52,838 | 1,776 | 153,968 | 49,330 | 56,347 | ,727 |
|  | MTR | 52,253 | 1,449 | 153,164 | 49,390 | 55,116 | ,727 |
| Month 3 | STR | 52,720 | 1,756 | 151,317 | 49,250 | 56,189 | ,787 |
|  | MTR | 52,274 | 1,441 | 151,496 | 49,428 | 55,121 | ,787 |
| Month 6 | STR | 52,541 | 1,735 | 148,717 | 49,113 | 55,969 | ,884 |
|  | MTR | 52,307 | 1,431 | 149,780 | 49,478 | 55,135 | ,884 |
| Month 12 | STR | 52,184 | 1,725 | 150,393 | 48,775 | 55,594 | ,907 |
|  | MTR | 52,371 | 1,425 | 149,681 | 49,555 | 55,186 | ,907 |
| Month 18 | STR | 51,828 | 1,761 | 164,029 | 48,351 | 55,304 | ,710 |
|  | MTR | 52,435 | 1,434 | 154,741 | 49,602 | 55,269 | ,710 |
| Month 24 | STR | 51,471 | 1,838 | 192,157 | 47,847 | 55,095 | ,553 |
|  | MTR | 52,500 | 1,460 | 165,598 | 49,618 | 55,381 | ,553 |

**MOS-HIV PHS: Graph**


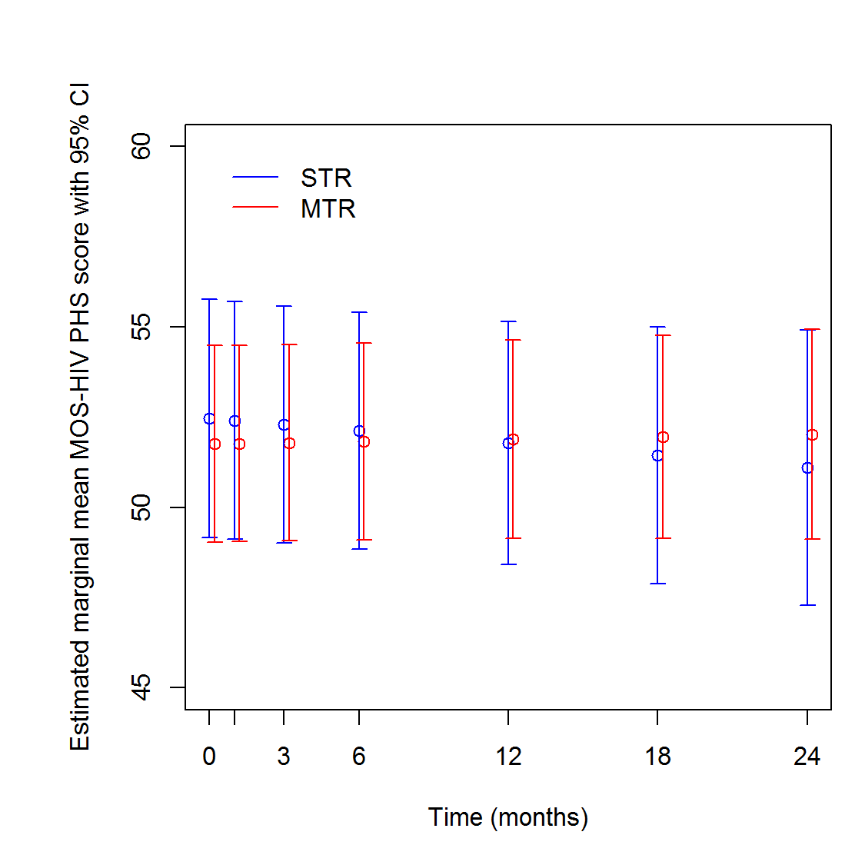


## Quality of Life: Mental health (Mental health score of MOS-HIV, standardized scale (T-score) with a mean of 50 and a standard deviation of 10 in the sample in which the summary scores were developed, higher score = better outcomes)

Model with random intercept, random slope, diagonal covariance structure and time categorical.
-2Restricted Log Likelihood: 5848.812
AIC: 5854.812
71,1% of data were available (936/1316).
165/188 (87.8%) people had 2 or more present values, if they would have all 7 values, there would be 1155 values.
However, only 81.0% of the values were present (936/1155) and could be included in the model.

| **MOS-HIV: Estimates of Fixed Effects** | | | | | | | |
| --- | --- | --- | --- | --- | --- | --- | --- |
| Parameter | Estimate | Std. Error | df | t | Sig. | 95% Confidence Interval | |
|  |  |  |  |  |  | Lower Bound | Upper Bound |
| Intercept | 51,064184 | 1,029414 | 186,264 | 49,605 | ,000 | 49,033376 | 53,094993 |
| Time continuous | ,016306 | ,027562 | 167,860 | ,592 | ,555 | -,038106 | ,070719 |
| STR group | -1,134118 | 1,857507 | 217,652 | -,611 | ,542 | -4,795123 | 2,526887 |
| MTR group | 0 | 0 | . | . | . | . | . |
| STR * Time continuous | -,013511 | ,057784 | 189,898 | -,234 | ,815 | -,127492 | ,100470 |
| MTR * Time continuous | 0 | 0 | . | . | . | . | . |
| Women | ,286379 | 2,600609 | 140,793 | ,110 | ,912 | -4,854912 | 5,427671 |
| Men | 0 | 0 | . | . | . | . | . |
| Non-Caucasian | 2,316769 | 3,246526 | 144,637 | ,714 | ,477 | -4,099994 | 8,733532 |
| Caucasian | 0 | 0 | . | . | . | . | . |
| Heterosexual | -1,807734 | 2,023077 | 141,968 | -,894 | ,373 | -5,806982 | 2,191515 |
| Homosexual | 0 | 0 | . | . | . | . | . |

Intercept: At 2y, MTR-, Caucasian, homosexual men have a MHS-score of 51.06 (p<0.001).

Time: Per month, PHS increases by 0.02 in the MTR-group. This increase is not significant (p=0.555)

Stable: At 2y, the STR-group has a MHS-score of 1.13 less than the MTR-group. This difference is not significant (p=0.542). *A second model (in which baseline is the reference category), the MHS-score in the STR-group at baseline is 0.73 lower than the MTR-group, this difference is not significant (0.659).*

Interaction: The estimated mean difference in MHS score per month is 0.01 lower in the STR-group than in the MTR-group (the 95% CI goes from 0.13 lower to 0.10 higher. This difference is not significant (p=0.815).

Differences according to gender, ethnicity and sexual orientation are not significant.

|  | **MOS-HIV MHS: Estimates** | | | | | |  |
| --- | --- | --- | --- | --- | --- | --- | --- |
|  | Group | Mean | Std. Error | Df | 95% Confidence Interval | |  |
| Time |  |  |  |  | Lower Bound | Upper Bound | Sig |
| Baseline | STR | 50,261 | 1,875 | 148,958 | 46,556 | 53,965 | ,648 |
|  | MTR | 51,071 | 1,527 | 148,965 | 48,053 | 54,088 | ,648 |
| Month 1 | STR | 50,263 | 1,863 | 147,729 | 46,583 | 53,944 | ,639 |
|  | MTR | 51,087 | 1,522 | 148,181 | 48,079 | 54,095 | ,639 |
| Month 3 | STR | 50,269 | 1,842 | 145,741 | 46,628 | 53,910 | ,622 |
|  | MTR | 51,119 | 1,514 | 146,906 | 48,128 | 54,111 | ,622 |
| Month 6 | STR | 50,277 | 1,822 | 144,269 | 46,677 | 53,878 | ,599 |
|  | MTR | 51,168 | 1,505 | 145,823 | 48,194 | 54,142 | ,599 |
| Month 12 | STR | 50,294 | 1,819 | 148,774 | 46,701 | 53,888 | ,563 |
|  | MTR | 51,266 | 1,500 | 147,161 | 48,301 | 54,232 | ,563 |
| Month 18 | STR | 50,311 | 1,866 | 166,294 | 46,627 | 53,995 | ,545 |
|  | MTR | 51,364 | 1,514 | 153,959 | 48,372 | 54,356 | ,545 |
| Month 24 | STR | 50,328 | 1,960 | 199,783 | 46,463 | 54,193 | ,542 |
|  | MTR | 51,462 | 1,546 | 166,950 | 48,410 | 54,514 | ,542 |

**MOS-HIV MHS: Graph**


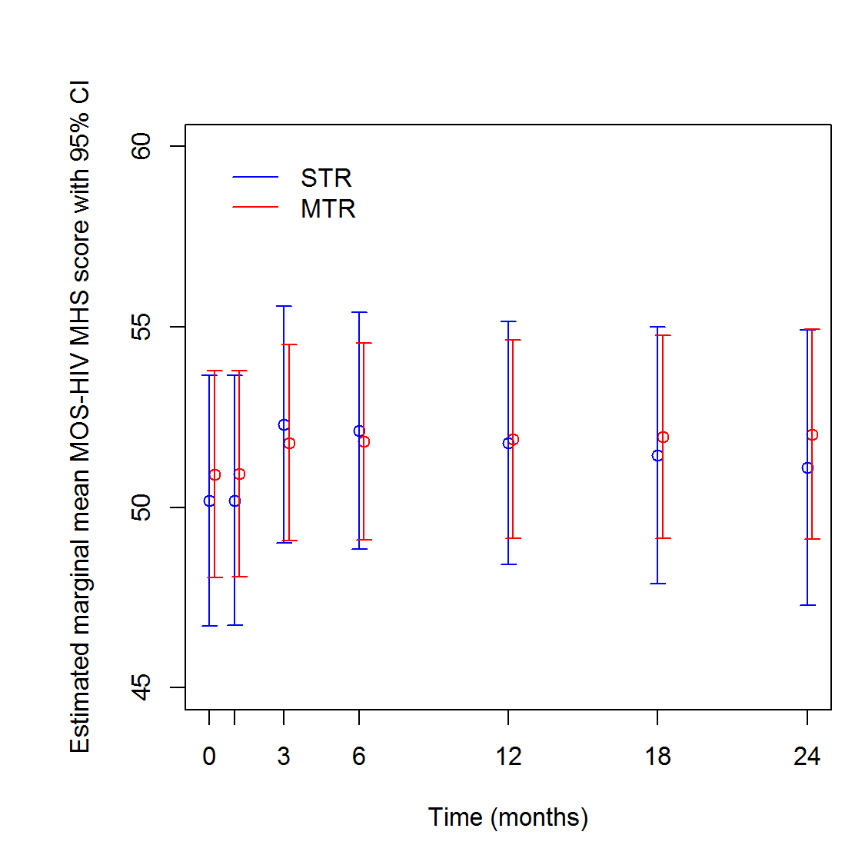


## Adherence: score (Case Adherence Index, range 0-16, higher score = better outcome)

Model with random intercept, random slope, diagonal covariance structure and time continuous.
-2Restricted Log Likelihood: 3247.883
AIC: 3253.883
71.2% of data were available (937/1316).
164 people had 2 or more present values, if they would have all 7 values, there would be 1148 values.
However, only 81.6% of the values were present (937/1148) and could be included in the model.

| **CASE Adherence Index: Estimates of Fixed Effects** | | | | | | | |
| --- | --- | --- | --- | --- | --- | --- | --- |
| Parameter | Estimate | Std. Error | df | t | Sig. | 95% Confidence Interval | |
|  |  |  |  |  |  | Lower Bound | Upper Bound |
| Intercept | 14,594108 | ,211082 | 194,190 | 69,140 | ,000 | 14,177801 | 15,010415 |
| Time continuous | -,001447 | ,006765 | 192,737 | -,214 | ,831 | -,014790 | ,011895 |
| STR group | -,246581 | ,381957 | 226,827 | -,646 | ,519 | -,999219 | ,506058 |
| MTR group | 0 | 0 | . | . | . | . | . |
| STR * Time continuous | ,000585 | ,013897 | 213,715 | ,042 | ,966 | -,026808 | ,027979 |
| MTR * Time continuous | 0 | 0 | . | . | . | . | . |
| Women | ,228065 | ,525067 | 138,886 | ,434 | ,665 | -,810092 | 1,266223 |
| Men | 0 | 0 | . | . | . | . | . |
| Non-Caucasian | -,705680 | ,661218 | 145,316 | -1,067 | ,288 | -2,012527 | ,601167 |
| Caucasian | 0 | 0 | . | . | . | . | . |
| Heterosexual | -,179592 | ,409394 | 140,427 | -,439 | ,662 | -,988965 | ,629780 |
| Homosexual | 0 | 0 | . | . | . | . | . |

Intercept: At 2y, MTR-, Caucasian, homosexual men have a CASE sumscore of 14.59 (p<0.001).

Time: Per month, the score decreases by 0.001 in the MTR-group. This increase is not significant (p=0.831)

Stable: At 2y, the STR-group has a CASE sumscore which is 0.25 lower than the MTR-group. This difference is not significant (p=0.519). *A second model (in which baseline is the reference category), the CASE sumscore in the STR-group at baseline is 0.26 lower than the MTR-group, this difference is not significant (0.433).*

Interaction: The estimated mean difference in MHS score per month is 0.0006 lower in the STR-group than in the MTR-group (the 95% CI goes from 0.027 lower to 0.028 higher. This difference is not significant (p=966).

Differences according to gender, ethnicity and sexual orientation are not significant.

|  | **CASE Adherence Index: Estimates** | | | | | |  |
| --- | --- | --- | --- | --- | --- | --- | --- |
|  | Group | Mean | Std. Error | Df | 95% Confidence Interval | |  |
| Time |  |  |  |  | Lower Bound | Upper Bound | Sig |
| Baseline | STR | 14,040 | ,393 | 150,833 | 13,263 | 14,816 | ,488 |
|  | MTR | 14,300 | ,316 | 151,243 | 13,675 | 14,926 | ,488 |
| Month 1 | STR | 14,039 | ,389 | 149,516 | 13,270 | 14,808 | ,483 |
|  | MTR | 14,299 | ,315 | 150,223 | 13,677 | 14,921 | ,483 |
| Month 3 | STR | 14,037 | ,382 | 147,227 | 13,281 | 14,793 | ,473 |
|  | MTR | 14,296 | ,312 | 148,494 | 13,679 | 14,913 | ,473 |
| Month 6 | STR | 14,034 | ,375 | 145,137 | 13,293 | 14,776 | ,461 |
|  | MTR | 14,292 | ,309 | 146,836 | 13,681 | 14,902 | ,461 |
| Month 12 | STR | 14,029 | ,371 | 148,944 | 13,296 | 14,762 | ,457 |
|  | MTR | 14,283 | ,306 | 147,832 | 13,677 | 14,889 | ,457 |
| Month 18 | STR | 14,024 | ,381 | 168,489 | 13,272 | 14,776 | ,478 |
|  | MTR | 14,274 | ,309 | 156,012 | 13,663 | 14,885 | ,478 |
| Month 24 | STR | 14,019 | ,404 | 208,537 | 13,222 | 14,816 | ,519 |
|  | MTR | 14,266 | ,318 | 172,694 | 13,639 | 14,892 | ,519 |

**CASE Adherence Index: Graph**


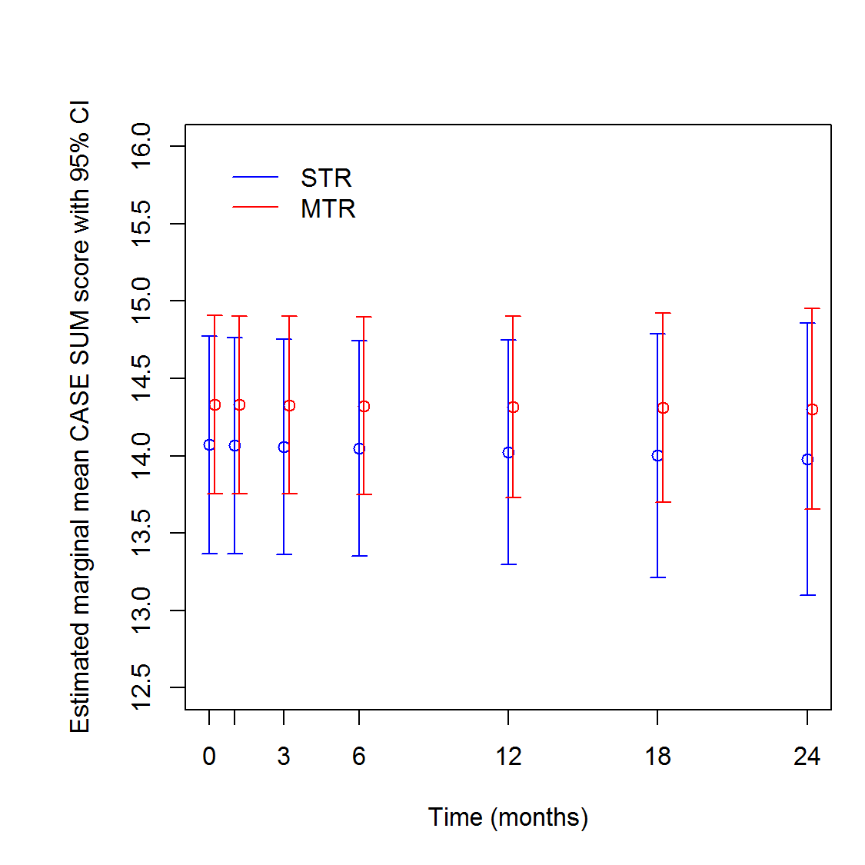


## Adherence: VAS (Visual Analogue Scale, range 0-100, higher score = better outcome)

Model with random intercept and time categorical.
-2Restricted Log Likelihood: -812.069
AIC: -806.069
70.5% of data were available (928/1316).
163/188 (86.7%) people had 2 or more present values, if they would have all 7 values, there would be 1141 values.
However, only 81.3% of the values were present (928/1141) and could be included in the model.

| **Adherence VAS: Estimates of Fixed Effects** | | | | | | | |
| --- | --- | --- | --- | --- | --- | --- | --- |
| Parameter | Estimate | Std. Error | df | t | Sig. | 95% Confidence Interval | |
|  |  |  |  |  |  | Lower Bound | Upper Bound |
| Intercept | 97,049934 | ,736541 | 412,904 | 131,765 | ,000 | 95,602097 | 98,497771 |
| Baseline | ,667390 | ,691099 | 717,110 | ,966 | ,335 | -,689430 | 2,024209 |
| Month 1 | ,151240 | ,709754 | 714,410 | ,213 | ,831 | -1,242213 | 1,544693 |
| Month 3 | ,565931 | ,704358 | 713,818 | ,803 | ,422 | -,816929 | 1,948791 |
| Month 6 | ,887951 | ,727685 | 710,539 | 1,220 | ,223 | -,540718 | 2,316621 |
| Month 12 | ,271333 | ,725191 | 707,890 | ,374 | ,708 | -1,152448 | 1,695115 |
| Month 18 | -,023136 | ,735895 | 708,147 | -,031 | ,975 | -1,467933 | 1,421660 |
| Month 24 | 0 | 0 | . | . | . | . | . |
| STR group | ,610098 | 1,395868 | 519,837 | ,437 | ,662 | -2,132138 | 3,352334 |
| MTR group | 0 | 0 | . | . | . | . | . |
| Baseline * STR group | -,797498 | 1,396093 | 719,315 | -,571 | ,568 | -3,538402 | 1,943406 |
| Baseline * MTR group | 0 | 0 | . | . | . | . | . |
| Month 1 * STR group | -1,602160 | 1,480913 | 723,029 | -1,082 | ,280 | -4,509563 | 1,305242 |
| Month 1 * MTR group | 0 | 0 | . | . | . | . | . |
| Month 3 * STR group | -,830453 | 1,435081 | 719,693 | -,579 | ,563 | -3,647899 | 1,986992 |
| Month 3 * MTR group | 0 | 0 | . | . | . | . | . |
| Month 6 * STR group | -,706149 | 1,454361 | 710,981 | -,486 | ,627 | -3,561506 | 2,149208 |
| Month 6 * MTR group | 0 | 0 | . | . | . | . | . |
| Month 12 * STR group | 1,045246 | 1,500561 | 711,120 | ,697 | ,486 | -1,900815 | 3,991306 |
| Month 12 * MTR group | 0 | 0 | . | . | . | . | . |
| Month 18 * STR group | -,387335 | 1,535404 | 709,335 | -,252 | ,801 | -3,401816 | 2,627146 |
| Month 18 * MTR group | 0 | 0 | . | . | . | . | . |
| Month 24 * STR group | 0 | 0 | . | . | . | . | . |
| Month 24 * MTR group | 0 | 0 | . | . | . | . | . |
| Women | -,849481 | 1,462691 | 137,577 | -,581 | ,562 | -3,741744 | 2,042782 |
| Men | 0 | 0 | . | . | . | . | . |
| Non-Caucasian | -6,044694 | 1,890744 | 148,044 | -3,197 | ,002 | -9,781026 | -2,308362 |
| Caucasian | 0 | 0 | . | . | . | . | . |
| Heterosexual | -,862408 | 1,140714 | 140,546 | -,756 | ,451 | -3,117584 | 1,392769 |
| Homosexual | 0 | 0 | . | . | . | . | . |

Intercept: AT 2y, MTR, Caucasian, homosexual men have a score of 97.05 (p<0.001).

Time:
Within the MTR-group, there are no significant differences over time as compared to 2y. A second model (STR-group as reference) shows no significant differences over time as compared to 2y within the STR-group. The third and fourth model (baseline+MTR and baseline+STR as reference groups) show no significant differences over time in both groups as compared to baseline.

Stable: At 2y, STR-patients have a 0.61 higher score as compared to MTR-patients. This difference is not significant (p=0.662). *A second model shows that at baseline, the STR-group’s score (-0.19) is not significantly different as compared to the score in the MTR-group (p=0.878)*

Interaction: The estimated mean difference between baseline and 2y between the groups is 0.80. This difference is not significant (p=0.568):
- VAS-score in the STR-group increased by 5.73, VAS-score in the MTR-group decreased by 0.67 over time.
- The STR-group had a 0.19 lower score at baseline and 0.61 higher at 2y.

Differences in ethnicity are significant: at 2y, non-Caucasian, homosexual, MTR-males have 6.04 points less than homosexual, Caucasian MTR-males (p=0.002).

| **Adherence VAS: Estimates** | | | | | | |  |
| --- | --- | --- | --- | --- | --- | --- | --- |
| Tijd | Group | Mean | Std. Error | df | 95% Confidence Interval | |  |
|  |  |  |  |  | Lower Bound | Upper Bound | Sig |
| Baseline | STR | 93,652 | 1,256 | 277,506 | 91,179 | 96,124 | ,878 |
|  | MTR | 93,839 | ,957 | 217,600 | 91,952 | 95,726 | ,878 |
| Month 1 | STR | 92,331 | 1,343 | 343,240 | 89,690 | 94,972 | ,454 |
|  | MTR | 93,323 | ,978 | 233,937 | 91,397 | 95,249 | ,454 |
| Month 3 | STR | 93,517 | 1,293 | 305,926 | 90,974 | 96,061 | ,862 |
|  | MTR | 93,738 | ,975 | 231,536 | 91,817 | 95,658 | ,862 |
| Month 6 | STR | 93,964 | 1,331 | 332,696 | 91,345 | 96,582 | ,942 |
|  | MTR | 94,060 | ,997 | 250,179 | 92,095 | 96,024 | ,942 |
| Month 12 | STR | 95,098 | 1,391 | 381,640 | 92,363 | 97,834 | ,229 |
|  | MTR | 93,443 | ,998 | 250,257 | 91,478 | 95,408 | ,229 |
| Month 18 | STR | 93,371 | 1,436 | 418,288 | 90,549 | 96,193 | ,876 |
|  | MTR | 93,149 | 1,006 | 257,960 | 91,167 | 95,130 | ,876 |
| Month 24 | STR | 93,782 | 1,401 | 388,572 | 91,028 | 96,535 | ,662 |
|  | MTR | 93,172 | 1,008 | 259,675 | 91,187 | 95,157 | ,662 |
|  | | | | | | |  |

**Adherence VAS: Graph**


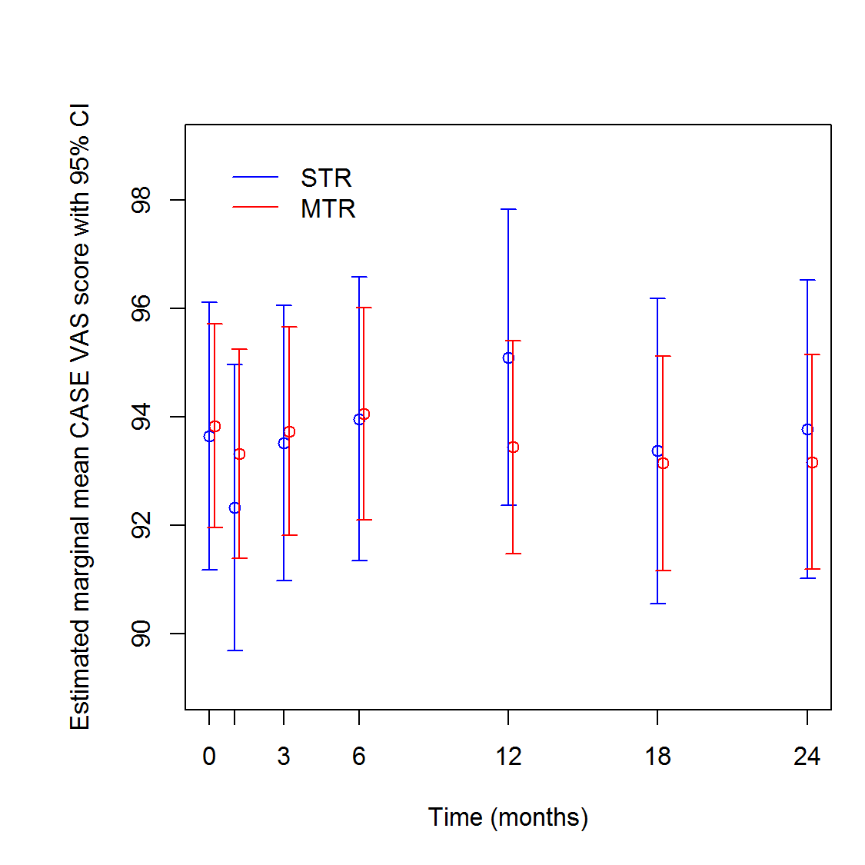


## Treatment satisfaction (HIV Treatment Satisfaction Questionnaire, 0-60, higher score = better outcome)

Model with random intercept and time categorical.
-2Restricted Log Likelihood: 2711.609
AIC: 2715.609
64.2% of data were available (483/752) (188*4 = 752)
161/188 (85.6%) people had 2 or more present values, if they would have all 4 values, there would be 644 values.
However, only 75% of the values were present (483/644) and could be included in the model.

| **Treatment Satisfaction: Estimates of Fixed Effects** | | | | | | | |
| --- | --- | --- | --- | --- | --- | --- | --- |
| Parameter | Estimate | Std. Error | df | T | Sig. | 95% Confidence Interval | |
|  |  |  |  |  |  | Lower Bound | Upper Bound |
| Intercept | 54,293673 | ,713986 | 208,171 | 76,043 | ,000 | 52,886103 | 55,701242 |
| Baseline | ,151972 | ,553642 | 326,739 | ,274 | ,784 | -,937182 | 1,241125 |
| Month 12 | ,108488 | ,564384 | 327,739 | ,192 | ,848 | -1,001784 | 1,218760 |
| Month 18 | -,148668 | ,581062 | 329,203 | -,256 | ,798 | -1,291732 | ,994396 |
| Month 24 | 0 | 0 | . | . | . | . | . |
| STR-group | 3,137894 | 1,350935 | 241,464 | 2,323 | ,021 | ,476772 | 5,799017 |
| MTR-group | 0 | 0 | . | . | . | . | . |
| Baseline * STR group | -3,496733 | 1,169928 | 327,671 | -2,989 | ,003 | -5,798250 | -1,195216 |
| Baseline * MTR group | 0 | 0 | . | . | . | . | . |
| Month 12 * STR group | -2,553884 | 1,193695 | 332,121 | -2,139 | ,033 | -4,902039 | -,205729 |
| Month 12 * MTR group | 0 | 0 | . | . | . | . | . |
| Month 18 * STR group | -2,516232 | 1,194444 | 328,912 | -2,107 | ,036 | -4,865946 | -,166518 |
| Month 18 * MTR group | 0 | 0 | . | . | . | . | . |
| Month 24 * STR group | 0 | 0 | . | . | . | . | . |
| Month 24 * MTR group | 0 | 0 | . | . | . | . | . |
| Women | ,128171 | 1,618611 | 123,057 | ,079 | ,937 | -3,075755 | 3,332097 |
| Men | 0 | 0 | . | . | . | . | . |
| Non-Caucasian | ,024943 | 2,209635 | 129,316 | ,011 | ,991 | -4,346773 | 4,396659 |
| Caucasian | 0 | 0 | . | . | . | . | . |
| Heterosexual | -1,036714 | 1,300589 | 122,110 | -,797 | ,427 | -3,611337 | 1,537909 |
| Homosexual | 0 | 0 | . | . | . | . | . |

Intercept: AT 2y, MTR, Caucasian, homosexual men have a score of 54.29 (p<0.001).

Time:
Within the MTR-group, there are no significant differences over time as compared to 2y. A second model (2y+STR-group as reference groups) shows significant differences over time as compared to 2y within the STR-group: at baseline a 3.34 lower score (p=0.001), at 1y a 2.45 lower score (p=0.021) and at month 18 a 2.66 lower score (p=0.011). The third model (baseline+MTR) shows no significant differences over time in the MTR-group as compared to baseline. The fourth model (baseline+STR as reference groups) shows that at 2y, STR-participants have a 3.34 higher score than at baseline (p=0.001).

Stable: At 2y, STR-patients have a 3.14 higher score as compared to MTR-patients, this difference is significant (p=0.021). *A second model shows that at baseline, the STR-group’s score (-0.36) is not significantly different as compared to the score in the MTR-group (p=0.786)*

Interaction: The estimated mean difference between baseline and 2y between the groups is 3.50. This difference is significant (p=0.003):
- The score in the STR-group increased by 3.3, the score in the MTR-group decreased by 0.2 over time.
- The STR-group had a 0.36 lower score at baseline and 3.14 higher at 2y.

Differences according to gender, ethnicity and sexual orientation are not significant.

| **Treatment Satisfaction: Estimates** | | | | | | |  |
| --- | --- | --- | --- | --- | --- | --- | --- |
| Time | Group | Mean | Std. Error | df | 95% Confidence Interval | |  |
|  |  |  |  |  | Lower Bound | Upper Bound | Sig |
| Baseline | STR | 53,645 | 1,431 | 191,288 | 50,823 | 56,467 | ,786 |
|  | MTR | 54,004 | 1,103 | 154,743 | 51,825 | 56,183 | ,786 |
| Month 12 | STR | 54,544 | 1,425 | 189,485 | 51,733 | 57,356 | ,659 |
|  | MTR | 53,960 | 1,108 | 157,041 | 51,773 | 56,148 | ,659 |
| Month 18 | STR | 54,325 | 1,439 | 195,137 | 51,487 | 57,163 | ,644 |
|  | MTR | 53,703 | 1,120 | 163,218 | 51,492 | 55,914 | ,644 |
| Month 24 | STR | 56,990 | 1,450 | 199,493 | 54,130 | 59,849 | ,021 |
|  | MTR | 53,852 | 1,112 | 159,538 | 51,655 | 56,048 | ,021 |
|  | | | | | | |  |

**Treatment Satisfaction: Graph**


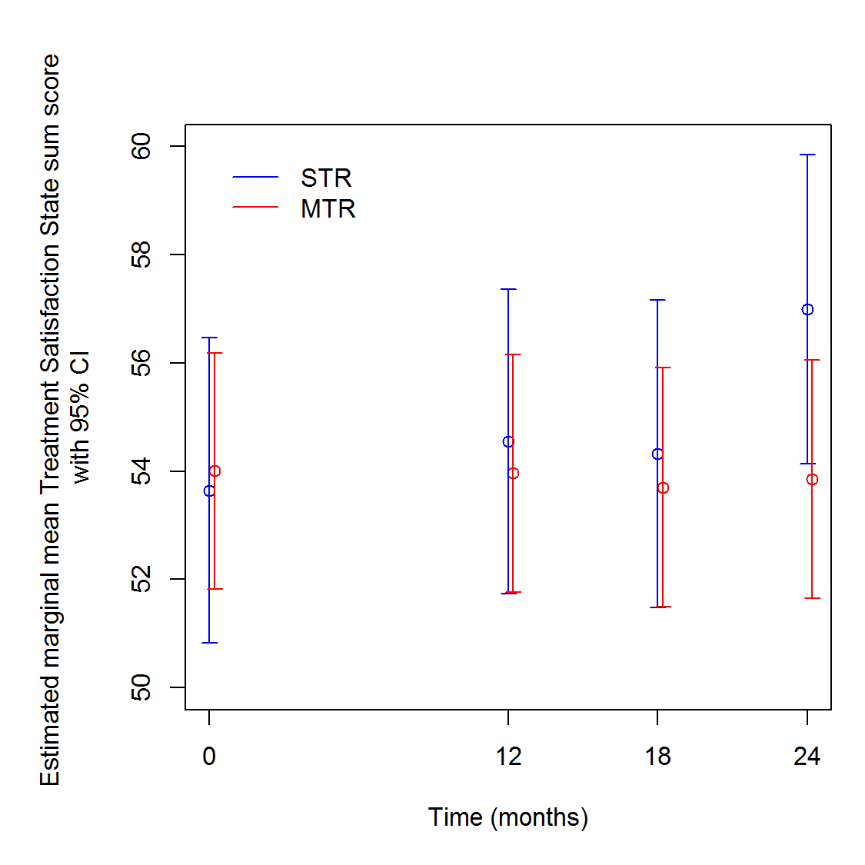


## Presence of neurocognitive complaints (Neurocognitive screening questions, yes/no, yes = worse outcome)

Generalized estimating equations model with exchangeable correlation matrix and time continuous.
QIC: 1243.474
QICC: 1220.693
71.4% of data were available (939/1316).
163/188 (86.7%) people had 2 or more present values, if they would have all 7 values, there would be 1141 values.
However, only 82.3% of the values were present (939/1141) and could be included in the model.

| **Neurocognitive complaints: Parameter Estimates** | | | | | | | | | | |
| --- | --- | --- | --- | --- | --- | --- | --- | --- | --- | --- |
| Parameter | B | Std. Error | 95% Wald Confidence Interval | | Hypothesis Test | | | Exp(B) | 95% Wald Confidence Interval for Exp(B) | |
|  |  |  | Lower | Upper | Wald Chi-Square | df | Sig. |  | Lower | Upper |
| Intercept | -,155 | ,2055 | -,558 | ,248 | ,570 | 1 | ,450 | ,856 | ,572 | 1,281 |
| Time continuous | -,001 | ,0069 | -,014 | ,013 | ,018 | 1 | ,892 | ,999 | ,986 | 1,013 |
| STR group | 1,100 | ,3487 | ,417 | 1,784 | 9,952 | 1 | ,002 | 3,005 | 1,517 | 5,952 |
| MTR group | 0 | . | . | . | . | . | . | 1 | . | . |
| STR * Time continuous | ,041 | ,0192 | ,003 | ,078 | 4,453 | 1 | ,035 | 1,041 | 1,003 | 1,081 |
| MTR * Time continuous | 0 | . | . | . | . | . | . | 1 | . | . |
| Women | ,301 | ,4689 | -,618 | 1,221 | ,413 | 1 | ,520 | 1,352 | ,539 | 3,389 |
| Men | 0 | . | . | . | . | . | . | 1 | . | . |
| Non-Caucasian | -,635 | ,5756 | -1,763 | ,493 | 1,217 | 1 | ,270 | ,530 | ,172 | 1,638 |
| Caucasian | 0 | . | . | . | . | . | . | 1 | . | . |
| Heterosexual | -,208 | ,3606 | -,915 | ,499 | ,333 | 1 | ,564 | ,812 | ,401 | 1,646 |
| Homosexual | 0 | . | . | . | . | . | . | 1 | . | . |
| (Scale) | 1 |  |  |  |  |  |  |  |  |  |
|  | | | | | | | | | | |
|  | | | | | | | | | | |

Intercept: The odds on NCC in MTR-group at 2y is 0.86 (p=0.450)
Time: ODDS of having NCC does not change in stable group (p=0.892)

Stable: At 2y: switchers have ODDS of 3.005 of having NCC as compared to stable group (0.002)

Interaction: As time increases 1 month, ODDS of having NCC increases with 4.1% among switch patients as compared to stable group (p=0.035). Other formulation: as time increases 1 month, ODDS of having NCC decreases with 4.1% (1/0.960 = 1.041) as compared to switch group

|  | **Neurocognitive complaints: Estimates** | | | | |  |
| --- | --- | --- | --- | --- | --- | --- |
|  | StableART | Mean | Std. Error | 95% Wald Confidence Interval | |  |
| Time |  |  |  | Lower | Upper | Sig |
| Baseline | STR | ,43 | ,092 | ,27 | ,61 | ,711 |
|  | MTR | ,40 | ,063 | ,29 | ,53 | ,711 |
| Month 1 | STR | ,44 | ,090 | ,28 | ,62 | ,612 |
|  | MTR | ,40 | ,062 | ,29 | ,53 | ,612 |
| Month 3 | STR | ,46 | ,086 | ,30 | ,63 | ,420 |
|  | MTR | ,40 | ,062 | ,29 | ,52 | ,420 |
| Month 6 | STR | ,49 | ,081 | ,34 | ,64 | ,188 |
|  | MTR | ,40 | ,061 | ,29 | ,52 | ,188 |
| Month 12 | STR | ,55 | ,074 | ,40 | ,69 | ,018 |
|  | MTR | ,40 | ,062 | ,28 | ,52 | ,018 |
| Month 18 | STR | ,61 | ,075 | ,45 | ,74 | ,003 |
|  | MTR | ,40 | ,064 | ,28 | ,53 | ,003 |
| Month 24 | STR | ,66 | ,081 | ,49 | ,80 | ,002 |
|  | MTR | ,40 | ,067 | ,27 | ,53 | ,002 |
|  |  | | | | |  |
|  |  | | | | |  |

**Neurocognitive complaints: Graph**


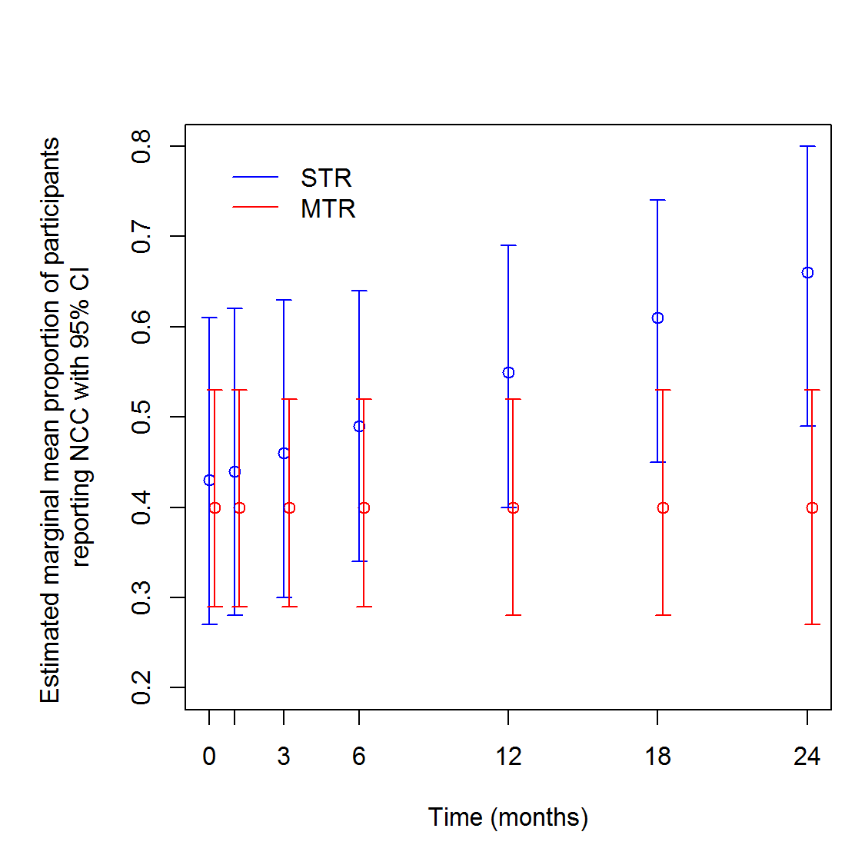

Supplement: S2 File — (DOCX) [file pone.0262533.s002.docx]
