# Supplementary material for: Patient-reported outcomes among people living with HIV on single- versus multi-tablet regimens: Data from a real-life setting
Source: PLoS One. 2022 Jan 13;17(1):e0262533. doi: 10.1371/journal.pone.0262533 (PMC8758085; doi:10.1371/journal.pone.0262533)

# **Mixed models for additional file: Bestworst scenario**

Missing lost-to-follow-up data were filled with good outcomes for STR-group and bad outcomes for MTR-group.

## Symptoms (HIV Symptoms Index, range 0-20, more symptoms = worse outcome)

Model with random intercept, random slope, unstructured covariance structure and time continuous.
-2Restricted Log Likelihood: 6705.724
Akaike’s Information Criterion (AIC): 6727.724
94.8% of data were available (1247/1316)

| **Symptoms: Estimates of Fixed Effects** | | | | | | | |
| --- | --- | --- | --- | --- | --- | --- | --- |
| Parameter | Estimate | Std. Error | df | t | Sig. | 95% Confidence Interval | |
|  |  |  |  |  |  | Lower Bound | Upper Bound |
| Intercept | 6,257757 | ,291107 | 1180 | 21,496 | ,000 | 5,686612 | 6,828903 |
| Time continuous | ,056731 | ,016453 | 1180,000 | 3,448 | ,001 | ,024451 | ,089011 |
| STR group | -3,956960 | ,520038 | 1180,000 | -7,609 | ,000 | -4,977263 | -2,936657 |
| MTR group | 0 | 0 | . | . | . | . | . |
| STR * Time continuous | -,138966 | ,030384 | 1180 | -4,574 | ,000 | -,198578 | -,079354 |
| MTR * Time continuous | 0 | 0 | . | . | . | . | . |
| Women | ,776380 | ,415631 | 1180,000 | 1,868 | ,062 | -,039079 | 1,591839 |
| Men | 0 | 0 | . | . | . | . | . |
| Non-Caucasian | -1,321505 | ,498121 | 1180,000 | -2,653 | ,008 | -2,298807 | -,344204 |
| Caucasian | 0 | 0 | . | . | . | . | . |
| Heterosexual | -,490018 | ,335191 | 1180,000 | -1,462 | ,144 | -1,147656 | ,167619 |
| Homosexual | 0 | 0 | . | . | . | . | . |
|  | | | | | | | |
|  | | | | | | | |

Intercept: At 2y MTR-, Caucasian, homosexual men have 6.26 symptoms (p<0.001).

Time: Per month, the number of symptoms increases by 0.06 in the MTR-group (p=0.001).
In STR-group, number of symptoms decreases by 0.08 (p=0.001).

Stable: At 2y, the STR-group has 3.96 symptoms less than the MTR-group (p<0.001)

Interaction: The estimated mean increase in symptoms per month is 0.14 lower among STR-group as compared to MTR-group (the 95% CI goes from 0.20 to 0.08 lower), p<0.001.

Non-Caucasian group has 1.32 symptoms less than Caucasian group at 2y (p=0.008)

| **Symptoms: Estimates** | | | | | | |  |
| --- | --- | --- | --- | --- | --- | --- | --- |
| Time Group | | Mean | Std. Error | Df | 95% Confidence Interval | |  |
|  |  |  |  |  | Lower Bound | Upper Bound | Sig |
| Baseline | STR | 3,757 | ,362 | 1180,000 | 3,047 | 4,467 | ,110 |
|  | MTR | 4,379 | ,285 | 1180,000 | 3,819 | 4,939 | ,110 |
| Month 1 | STR | 3,675 | ,346 | 1180,000 | 2,997 | 4,353 | ,039 |
|  | MTR | 4,435 | ,277 | 1180,000 | 3,892 | 4,979 | ,039 |
| Month 3 | STR | 3,510 | ,316 | 1180,000 | 2,889 | 4,131 | ,002 |
|  | MTR | 4,549 | ,263 | 1180,000 | 4,034 | 5,064 | ,002 |
| Month 6 | STR | 3,263 | ,285 | 1180,000 | 2,705 | 3,822 | <0,001 |
|  | MTR | 4,719 | ,248 | 1180,000 | 4,233 | 5,205 | <0,001 |
| Month 12 | STR | 2,770 | ,280 | 1180,000 | 2,221 | 3,319 | <0,001 |
|  | MTR | 5,059 | ,246 | 1180,000 | 4,576 | 5,543 | <0,001 |
| Month 18 | STR | 2,277 | ,350 | 1180,000 | 1,591 | 2,963 | <0,001 |
|  | MTR | 5,400 | ,282 | 1180,000 | 4,847 | 5,953 | <0,001 |
| Month 24 | STR | 1,783 | ,462 | 1180,000 | ,877 | 2,690 | <0,001 |
|  | MTR | 5,740 | ,343 | 1180,000 | 5,067 | 6,414 | <0,001 |

**Symptoms: Graph**


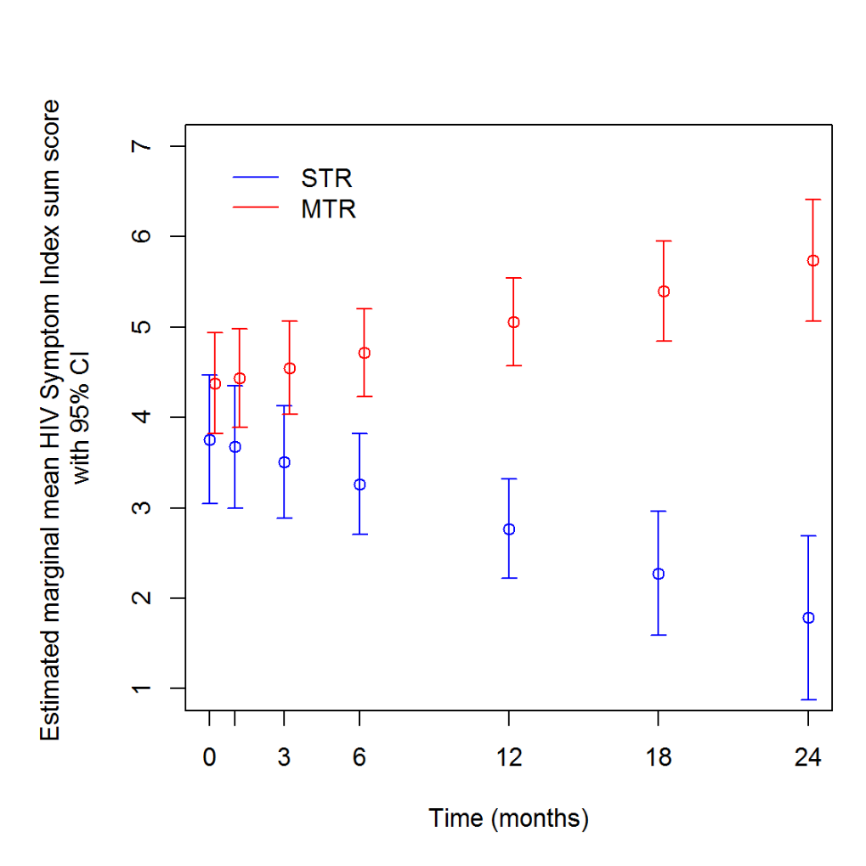


## Depressive symptoms (Beck Depression Inventory II, range 0-63, higher score = worse outcome)

Model with random intercept, random slope, unstructured covariance structure and time continuous.
-2Restricted Log Likelihood: 7753.445
AIC: 7775.445
94.7% of data were available (1246/1316)

| **BDI: Estimates of Fixed Effects** | | | | | | | |
| --- | --- | --- | --- | --- | --- | --- | --- |
| Parameter | Estimate | Std. Error | df | t | Sig. | 95% Confidence Interval | |
|  |  |  |  |  |  | Lower Bound | Upper Bound |
| Intercept | 11,660447 | ,936934 | 191,118 | 12,445 | ,000 | 9,812388 | 13,508505 |
| Time continuous | ,074549 | ,030319 | 170,017 | 2,459 | ,015 | ,014699 | ,134399 |
| STR group | -6,101210 | 1,606320 | 179,685 | -3,798 | ,000 | -9,270888 | -2,931533 |
| MTR group | 0^b^ | 0 | . | . | . | . | . |
| STR * Time continuous | -,206175 | ,055866 | 171,627 | -3,691 | ,000 | -,316448 | -,095902 |
| MTR * Time continuous | 0^b^ | 0 | . | . | . | . | . |
| Women | 1,633693 | 2,098934 | 174,734 | ,778 | ,437 | -2,508833 | 5,776219 |
| Men | 0^b^ | 0 | . | . | . | . | . |
| Non-Caucasian | -4,476394 | 2,495176 | 175,282 | -1,794 | ,075 | -9,400849 | ,448061 |
| Caucasian | 0^b^ | 0 | . | . | . | . | . |
| Heterosexual | -,402349 | 1,686871 | 174,976 | -,239 | ,812 | -3,731581 | 2,926883 |
| Homosexual | 0^b^ | 0 | . | . | . | . | . |
|  | | | | | | | |
|  | | | | | | | |

Intercept: At 2y MTR-, Caucasian, homosexual men have 11.7 depressive symptoms (p<0.001).

Time: Per month, the number of depressive symptoms increases by 0.07 in MTR-group (p=0.015). Per month, the number of depressive symptoms decreases by 0.131 in the STR-group (p=0.006).

Stable: At 2y, the STR-group has 6.10 depressive symptoms less than the MTR-group (p<0.001)

Interaction: The estimated mean increase in BDI score per month is 0.21 higher under MTR than under STR (the 95% CI goes from 0.10 higher to 0.32 higher (p<0.001).

Differences according to gender, ethnicity and sexual orientation are not significant.

|  | **BDI: Estimates** | | | | | |  |
| --- | --- | --- | --- | --- | --- | --- | --- |
|  | Group | Mean | Std. Error | df | 95% Confidence Interval | |  |
| Time |  |  |  |  | Lower Bound | Upper Bound | Sig |
| Baseline | STR | 7,096 | 1,415 | 185,091 | 4,304 | 9,887 | ,422 |
|  | MTR | 8,249 | 1,231 | 185,543 | 5,820 | 10,677 | ,422 |
| Month 1 | STR | 6,964 | 1,402 | 183,214 | 4,197 | 9,731 | ,338 |
|  | MTR | 8,323 | 1,225 | 183,408 | 5,906 | 10,740 | ,338 |
| Month 3 | STR | 6,701 | 1,382 | 179,899 | 3,975 | 9,427 | ,203 |
|  | MTR | 8,472 | 1,215 | 179,859 | 6,074 | 10,871 | ,203 |
| Month 6 | STR | 6,306 | 1,362 | 176,476 | 3,618 | 8,994 | ,080 |
|  | MTR | 8,696 | 1,206 | 176,500 | 6,315 | 11,077 | ,080 |
| Month 12 | STR | 5,516 | 1,367 | 176,925 | 2,819 | 8,214 | ,009 |
|  | MTR | 9,143 | 1,209 | 177,390 | 6,757 | 11,529 | ,009 |
| Month 18 | STR | 4,726 | 1,428 | 186,285 | 1,909 | 7,544 | ,001 |
|  | MTR | 9,591 | 1,239 | 188,119 | 7,147 | 12,034 | ,001 |
| Month 24 | STR | 3,937 | 1,539 | 198,896 | ,902 | 6,972 | <0,001 |
|  | MTR | 10,038 | 1,294 | 206,305 | 7,488 | 12,588 | <0,001 |

**BDI: Graph**


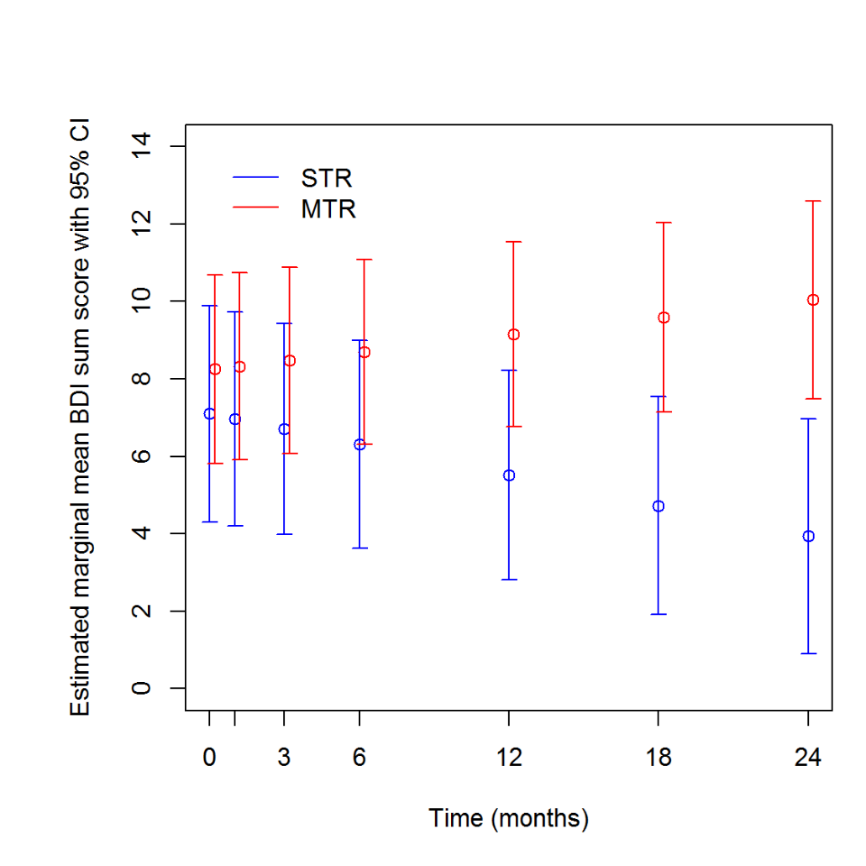


## Quality of life: EuroQol Utility (EuroQol 5D-3L, range 0-1, higher score = better outcome)

Model with random intercept and time categorical.
-2Restricted Log Likelihood: -1060.350
AIC: -1056.350
94.4% of data were available (1243/1316).

| **EuroQol Utility: Estimates of Fixed Effects** | | | | | | | |
| --- | --- | --- | --- | --- | --- | --- | --- |
| Parameter | Estimate | Std. Error | df | t | Sig. | 95% Confidence Interval | |
|  |  |  |  |  |  | Lower Bound | Upper Bound |
| Intercept | ,790408 | ,018130 | 368,108 | 43,596 | ,000 | ,754757 | ,826060 |
| Baseline | ,040812 | ,016012 | 992,837 | 2,549 | ,011 | ,009390 | ,072235 |
| Month 1 | ,016966 | ,016376 | 994,400 | 1,036 | ,300 | -,015170 | ,049102 |
| Month 3 | ,002347 | ,016090 | 993,224 | ,146 | ,884 | -,029226 | ,033921 |
| Month 6 | -,009905 | ,016334 | 994,267 | -,606 | ,544 | -,041957 | ,022147 |
| Month 12 | -,042640 | ,016170 | 993,660 | -2,637 | ,008 | -,074371 | -,010909 |
| Month 18 | ,002667 | ,016292 | 994,069 | ,164 | ,870 | -,029303 | ,034637 |
| Month 24 | 0 | 0 | . | . | . | . | . |
| STR group | ,124365 | ,031307 | 412,720 | 3,972 | ,000 | ,062825 | ,185905 |
| MTR group | 0 | 0 | . | . | . | . | . |
| Baseline * STR group | -,167146 | ,029281 | 992,405 | -5,708 | ,000 | -,224607 | -,109686 |
| Baseline * MTR group | 0 | 0 | . | . | . | . | . |
| Month 1 * STR group | -,076582 | ,030983 | 996,378 | -2,472 | ,014 | -,137381 | -,015783 |
| Month 1 * MTR group | 0 | 0 | . | . | . | . | . |
| Month 3 * STR group | -,063733 | ,029556 | 993,129 | -2,156 | ,031 | -,121732 | -,005734 |
| Month 3 * MTR group | 0 | 0 | . | . | . | . | . |
| Month 6 * STR group | -,043847 | ,029815 | 993,848 | -1,471 | ,142 | -,102354 | ,014661 |
| Month 6 * MTR group | 0 | 0 | . | . | . | . | . |
| Month 12 * STR group | -,016297 | ,029720 | 993,519 | -,548 | ,584 | -,074619 | ,042025 |
| Month 12 * MTR group | 0 | 0 | . | . | . | . | . |
| Month 18 * STR group | -,034589 | ,029789 | 993,755 | -1,161 | ,246 | -,093046 | ,023868 |
| Month 18 * MTR group | 0 | 0 | . | . | . | . | . |
| Month 24 * STR group | 0 | 0 | . | . | . | . | . |
| Month 24 * MTR group | 0 | 0 | . | . | . | . | . |
| Women | -,048586 | ,038619 | 174,567 | -1,258 | ,210 | -,124807 | ,027634 |
| Men | 0 | 0 | . | . | . | . | . |
| Non-Caucasian | ,082676 | ,045914 | 175,189 | 1,801 | ,073 | -,007939 | ,173292 |
| Caucasian | 0 | 0 | . | . | . | . | . |
| Heterosexual | ,005511 | ,031034 | 174,723 | ,178 | ,859 | -,055739 | ,066761 |
| Homosexual | 0 | 0 | . | . | . | . | . |

Intercept:
At 2y, MTR, Caucasian, homosexual men have a utility of 0.7904 (p<0.001)

Time:

Within the MTR-group:
at baseline, 0.041 less than at 2y (0.011)
at 1y, 0.043 less than at 2y (p = 0.008)

*A second model (in which 2y+STR-group are the reference groups) shows significant different scores within the STR-group as compared to 2y:
at baseline 0.126 less than at 2y (<0.001)
at T1 0.060 less than at 2y (0.024)
at T2 0.061 less than at 2y (0.013)
at T3 0.054 less than at 2y (0.031)
at T4 0.059 less than at 2y (0.018)*

*A third model (in which baseline+MTR are the reference groups) shows significantly different scores within the MTR-group as compared to baseline:
at T2 0.038 less than at baseline (0.018)
at T3 0.051 less than at baseline (0.002)
at T4 0.083 less than at baseline (<0.001)
at T5 0.038 less than at baseline (0.020)
at T6 0.041 less than at baseline (<0.001)*

*A fourth model (in which baseline+STR are the reference groups) shows significant differences within the STR-group as compared to baseline:
at T1 0.067 more than at baseline (0.011)
at T2 0.065 more than at baseline (0.009)
at T3 0.073 more than at baseline (0.004)
at T4 0.067 more than at baseline (0.007)
at T5 0.094 more than at baseline (<0.001)
at T6 0.126 more than at baseline (<0.001)*

Stable:
At 2y, the STR-group’s utility is significantly higher (+0.12) as compared to the utility in the MTR-group (p<0.001).

Interaction:
The estimated mean utility difference between baseline and 2y between the groups is 0.167 (p<0.001)
- Utility in the STR-group increased with 0.127, utility in the MTR-group decreased with 0.041 over time
- The STR-group had a 0.043 lower utility at baseline and 0.125 higher at 2y.

Differences according to gender, ethnicity and sexual orientation are not significant.

| **EuroQol Utility: Estimates** | | | | | | |  |
| --- | --- | --- | --- | --- | --- | --- | --- |
| Time | Group | Mean | Std. Error | df | 95% Confidence Interval | |  |
|  |  |  |  |  | Lower Bound | Upper Bound | Sig |
| Baseline | STR | ,808 | ,030 | 336,892 | ,750 | ,866 | ,173 |
|  | MTR | ,851 | ,025 | 261,844 | ,803 | ,899 | ,173 |
| Month 1 | STR | ,875 | ,031 | 398,546 | ,814 | ,936 | ,148 |
|  | MTR | ,827 | ,025 | 271,709 | ,778 | ,876 | ,148 |
| Month 3 | STR | ,873 | ,030 | 344,902 | ,815 | ,932 | ,056 |
|  | MTR | ,813 | ,025 | 262,510 | ,764 | ,861 | ,056 |
| Month 6 | STR | ,881 | ,030 | 353,664 | ,822 | ,940 | ,012 |
|  | MTR | ,800 | ,025 | 268,349 | ,752 | ,849 | ,012 |
| Month 12 | STR | ,876 | ,030 | 351,580 | ,817 | ,935 | ,001 |
|  | MTR | ,768 | ,025 | 265,765 | ,719 | ,816 | ,001 |
| Month 18 | STR | ,903 | ,030 | 352,509 | ,844 | ,962 | ,005 |
|  | MTR | ,813 | ,025 | 268,951 | ,764 | ,862 | ,005 |
| Month 24 | STR | ,935 | ,030 | 336,892 | ,876 | ,993 | <0,001 |
|  | MTR | ,810 | ,024 | 258,018 | ,762 | ,858 | <0,001 |
|  | | | | | | |  |

**EuroQol Utility: Graph**


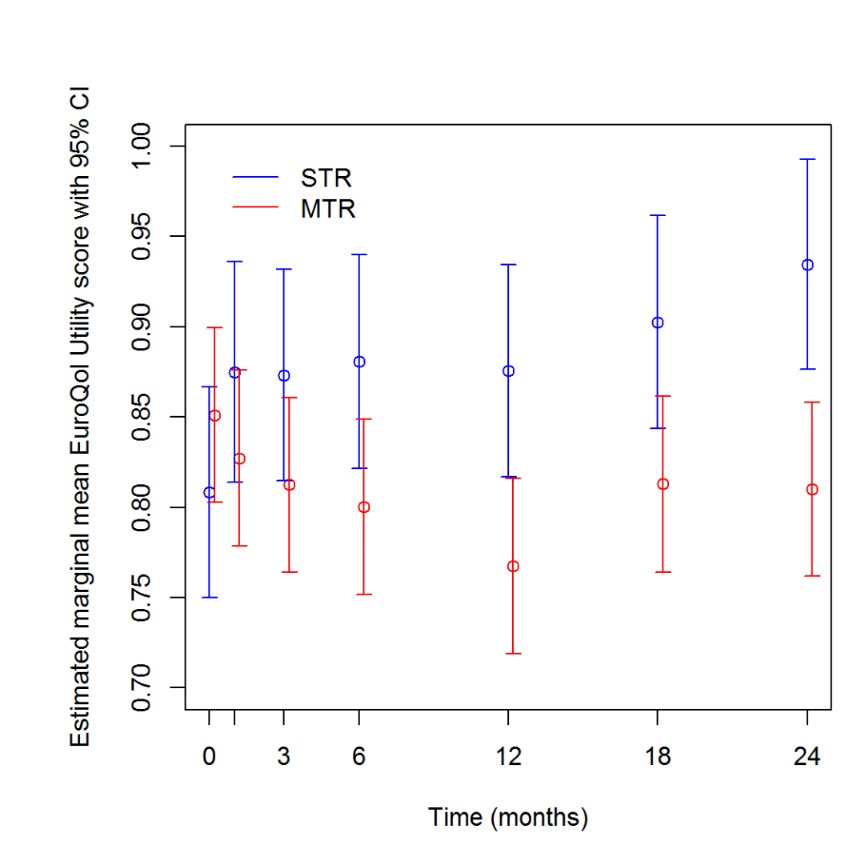


## Quality of Life: Visual Analogue Scale (EuroQol VAS, range 0-100, higher score = better outcome)

Model with random intercept, random slope, diagonal covariance structure and time categorical.
-2Restricted Log Likelihood: 8788.208
AIC: 8828.208
94.4% of data were available (1242/1316).

| **EuroQol VAS: Estimates of Fixed Effects** | | | | | | | |
| --- | --- | --- | --- | --- | --- | --- | --- |
| Parameter | Estimate | Std. Error | df | t | Sig. | 95% Confidence Interval | |
|  |  |  |  |  |  | Lower Bound | Upper Bound |
| Intercept | 76,418306 | 1,336709 | 281,193 | 57,169 | ,000 | 73,787079 | 79,049533 |
| Baseline | 2,184561 | 1,117954 | 457,918 | 1,954 | ,051 | -,012396 | 4,381518 |
| Month 1 | ,116522 | 1,132463 | 533,999 | ,103 | ,918 | -2,108107 | 2,341151 |
| Month 3 | ,233975 | 1,102956 | 635,112 | ,212 | ,832 | -1,931908 | 2,399857 |
| Month 6 | 1,789960 | 1,103366 | 839,437 | 1,622 | ,105 | -,375720 | 3,955641 |
| Month 12 | ,807137 | 1,074835 | 990,483 | ,751 | ,453 | -1,302077 | 2,916352 |
| Month 18 | -,297362 | 1,061610 | 891,531 | -,280 | ,779 | -2,380908 | 1,786184 |
| Month 24 | 0 | 0 | . | . | . | . | . |
| STR group | 12,279294 | 2,309540 | 288,826 | 5,317 | ,000 | 7,733630 | 16,824957 |
| MTR group | 0 | 0 | . | . | . | . | . |
| Baseline * STR group | -14,106259 | 2,042105 | 451,192 | -6,908 | ,000 | -18,119478 | -10,093041 |
| Baseline * MTR group | 0 | 0 | . | . | . | . | . |
| Month 1 * STR group | -6,521874 | 2,136796 | 567,000 | -3,052 | ,002 | -10,718875 | -2,324872 |
| Month 1 * MTR group | 0 | 0 | . | . | . | . | . |
| Month 3 * STR group | -7,644622 | 2,026205 | 634,772 | -3,773 | ,000 | -11,623498 | -3,665747 |
| Month 3 * MTR group | 0 | 0 | . | . | . | . | . |
| Month 6 * STR group | -4,826396 | 2,007150 | 828,020 | -2,405 | ,016 | -8,766096 | -,886695 |
| Month 6 * MTR group | 0 | 0 | . | . | . | . | . |
| Month 12 * STR group | -3,694820 | 1,968814 | 990,476 | -1,877 | ,061 | -7,558346 | ,168706 |
| Month 12 * MTR group | 0 | 0 | . | . | . | . | . |
| Month 18 * STR group | -3,234818 | 1,944022 | 890,061 | -1,664 | ,096 | -7,050221 | ,580584 |
| Month 18 * MTR group | 0 | 0 | . | . | . | . | . |
| Month 24 * STR group | 0 | 0 | . | . | . | . | . |
| Month 24 * MTR group | 0 | 0 | . | . | . | . | . |
| Women | -1,639854 | 2,833835 | 173,678 | -,579 | ,564 | -7,233042 | 3,953335 |
| Men | 0 | 0 | . | . | . | . | . |
| Non-Caucasian | 8,741250 | 3,366538 | 173,788 | 2,597 | ,010 | 2,096686 | 15,385815 |
| Caucasian | 0 | 0 | . | . | . | . | . |
| Heterosexual | -3,613199 | 2,278698 | 174,083 | -1,586 | ,115 | -8,110632 | ,884233 |
| Homosexual | 0 | 0 | . | . | . | . | . |

Intercept:
At 2y, MTR, Caucasian, homosexual men have a score of 76.42 (p<0.001)

Time:

Within the MTR-group, no differences as compared to 2y.

*A second model (in which 2y+STR-group are the reference groups) shows significant different scores within the STR-group at baseline (-11.92, p<0.001), month 1 (-6.41, p<0.001) month 3 (-7.41, p<0.001) and at month 18 (-3.53, p=0.030) as compared to 2y.*

*A third model (in which baseline+MTR are the reference groups) shows a significantly different score within the MTR-group at month 18 (+2.48, p=0.025) as compared to baseline.*

*A fourth model (in which baseline+STR are the reference groups) shows only a significant difference within the STR-group at month 1 (+5.52, p=0.001), at month 3 (+4.51, p=0.005), at month 6 (+8.89, p<0.001), at 1y (+9.03, p<0.001), at month 18 (+8.39, p<0.001) and at 2y (+11.92, p<0.001) as compared to baseline.*

Stable: At 2y, the score in the STR-group is 12.28 points higher (p<0.001).

Interaction:
The estimated mean difference between baseline and 2y between the groups is 14.11. This difference is significant (p<0.001)
- VAS-score in the STR-group increased by 11.92, the score in the MTR-group decreased by 2.19 over time
- The STR-group had a 1.83 higher score at baseline and 12.18 higher at 2y.

Differences according to gender and sexual orientation are not significant. We do see that at 2y, non-Caucasian participants’ score is 8.74 higher than Caucasian participants (p=0.010).

| **EuroQoL VAS: Estimates** | | | | | | |  |
| --- | --- | --- | --- | --- | --- | --- | --- |
| Time | Group | Mean | Std. Error | df | 95% Confidence Interval | |  |
|  |  |  |  |  | Lower Bound | Upper Bound | Sig |
| Baseline | STR | 78,520 | 2,092 | 292,856 | 74,402 | 82,638 | ,406 |
|  | MTR | 80,347 | 1,760 | 239,532 | 76,880 | 83,813 | ,406 |
| Month 1 | STR | 84,036 | 2,185 | 341,075 | 79,739 | 88,333 | ,012 |
|  | MTR | 78,279 | 1,774 | 246,766 | 74,785 | 81,772 | ,012 |
| Month 3 | STR | 83,031 | 2,106 | 299,773 | 78,888 | 87,175 | ,037 |
|  | MTR | 78,396 | 1,760 | 239,603 | 74,930 | 81,863 | ,037 |
| Month 6 | STR | 87,405 | 2,113 | 304,130 | 83,247 | 91,564 | ,001 |
|  | MTR | 79,952 | 1,771 | 245,030 | 76,465 | 83,440 | ,001 |
| Month 12 | STR | 87,554 | 2,137 | 314,005 | 83,350 | 91,758 | <0,001 |
|  | MTR | 78,970 | 1,778 | 247,699 | 75,467 | 82,472 | <0,001 |
| Month 18 | STR | 86,910 | 2,166 | 311,172 | 82,648 | 91,171 | <0,001 |
|  | MTR | 77,865 | 1,793 | 249,510 | 74,334 | 81,397 | <0,001 |
| Month 24 | STR | 90,442 | 2,182 | 272,877 | 86,146 | 94,738 | <0,001 |
|  | MTR | 78,162 | 1,798 | 237,670 | 74,620 | 81,704 | <0,001 |

**EuroQoL VAS: Graph**


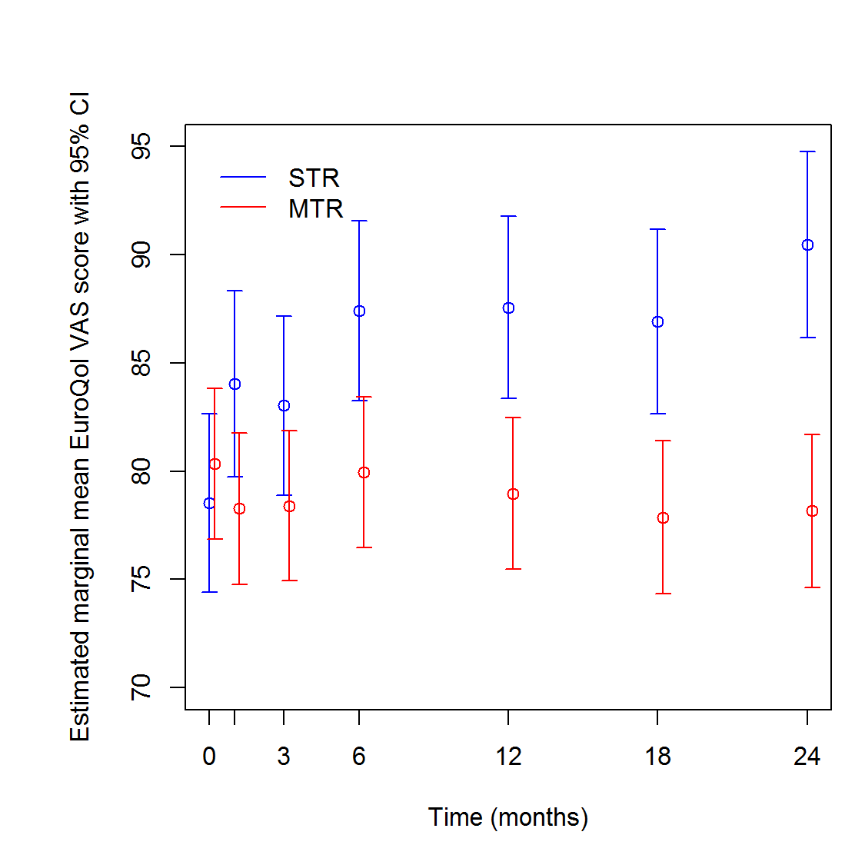


## Quality of Life: Physical health (Physical health score of MOS-HIV, standardized scale (T-score) with a mean of 50 and a standard deviation of 10 in the sample in which the summary scores were developed, higher score = better outcomes)

Model with random intercept, random slope, unstructured covariance structure and time categorical.
-2Restricted Log Likelihood: 7659.099
AIC: 7701.099
94.4% of data were available (1243/1316).

| **MOS-HIV PHS: Estimates of Fixed Effects** | | | | | | | |
| --- | --- | --- | --- | --- | --- | --- | --- |
| Parameter | Estimate | Std. Error | df | t | Sig. | 95% Confidence Interval | |
|  |  |  |  |  |  | Lower Bound | Upper Bound |
| Intercept | 47,908458 | ,942854 | 224,874 | 50,812 | ,000 | 46,050499 | 49,766417 |
| Baseline | 3,153828 | ,807692 | 277,724 | 3,905 | ,000 | 1,563851 | 4,743804 |
| Month 1 | 2,237448 | ,805612 | 319,758 | 2,777 | ,006 | ,652478 | 3,822418 |
| Month 3 | 1,087592 | ,765481 | 369,784 | 1,421 | ,156 | -,417649 | 2,592834 |
| Month 6 | ,829009 | ,732441 | 523,382 | 1,132 | ,258 | -,609877 | 2,267895 |
| Month 12 | ,054965 | ,658391 | 907,768 | ,083 | ,933 | -1,237181 | 1,347111 |
| Month 18 | ,036018 | ,619422 | 935,947 | ,058 | ,954 | -1,179598 | 1,251634 |
| Month 24 | 0 | 0 | . | . | . | . | . |
| STR group | 7,535734 | 1,632896 | 213,866 | 4,615 | ,000 | 4,317104 | 10,754364 |
| MTR group | 0 | 0 | . | . | . | . | . |
| Baseline * STR group | -8,003860 | 1,486365 | 278,982 | -5,385 | ,000 | -10,929775 | -5,077945 |
| Baseline * MTR group | 0 | 0 | . | . | . | . | . |
| Month 1 * STR group | -3,665439 | 1,508144 | 339,280 | -2,430 | ,016 | -6,631928 | -,698949 |
| Month 1 * MTR group | 0 | 0 | . | . | . | . | . |
| Month 3 * STR group | -3,347215 | 1,411984 | 374,021 | -2,371 | ,018 | -6,123637 | -,570793 |
| Month 3 * MTR group | 0 | 0 | . | . | . | . | . |
| Month 6 * STR group | -2,029367 | 1,339952 | 516,414 | -1,515 | ,131 | -4,661795 | ,603061 |
| Month 6 * MTR group | 0 | 0 | . | . | . | . | . |
| Month 12 * STR group | -1,993236 | 1,210069 | 907,928 | -1,647 | ,100 | -4,368092 | ,381621 |
| Month 12 * MTR group | 0 | 0 | . | . | . | . | . |
| Month 18 * STR group | -,974813 | 1,131975 | 935,751 | -,861 | ,389 | -3,196318 | 1,246691 |
| Month 18 * MTR group | 0 | 0 | . | . | . | . | . |
| Month 24 * STR group | 0 | 0 | . | . | . | . | . |
| Month 24 * MTR group | 0 | 0 | . | . | . | . | . |
| Women | -1,778288 | 1,962723 | 173,242 | -,906 | ,366 | -5,652216 | 2,095641 |
| Men | 0 | 0 | . | . | . | . | . |
| Non-Caucasian | 6,279357 | 2,332776 | 173,669 | 2,692 | ,008 | 1,675116 | 10,883599 |
| Caucasian | 0 | 0 | . | . | . | . | . |
| Heterosexual | -1,688282 | 1,577907 | 173,702 | -1,070 | ,286 | -4,802621 | 1,426057 |
| Homosexual | 0 | 0 | . | . | . | . | . |

Intercept: At 2y, MTR-, Caucasian, homosexual men have a PHS-score of 47.91 (p<0.001).

Time:

Within the MTR-group, at baseline there is a 3.15 higher score as compared to 2y (p<0.001) and at month 1, a 2.24 higher score (p=0.006)

*A second model (in which 2y+STR-group are the reference groups) shows a significant different score within the STR-group at baseline (-4.85, p<0.001) as compared to 2y.*

*A third model (in which baseline+MTR are the reference groups) shows significantly different scores within the MTR-group at month 1 (+3.42, p=0.001), month 3 (+2.59, p=0.006), month 6 (+3.65, p<0.001), at 1y (+2.91, p=0.004), at month 18 (+3.91, p=0.001) and at 2y (+4.85, p<0.001) as compared to baseline.*

*A fourth model (in which baseline+STR are the reference groups) shows only a significant difference within the STR-group as compared to baseline at month 3 (-2.07, p=0.001), at month 6 (-2.32, p<0.001), at 1y (-3.10, p<0.001), at month 18 (-3.12, p<0.001) and at 2y (-3.15, p<0.001).*

Stable: At 2y, the STR-group has a PHS-score of 7.54 more than the MTR group (p<0.001).

Interaction: The estimated mean difference in PHS score between the groups over time is 8.00 (p<0.001) (the 95% CI goes from -10.93 to -5.08)

- PHS-score in the STR-group increased by 4.85, the score in the MTR-group decreased by 3.15 over time
- The STR-group had a 0.47 lower score at baseline and 7.54 higher at 2y.

Differences according to gender and sexual orientation are not significant.
Non-Caucasian participants have a 6.28 points higher PHS-score than Caucasian participants at 2y (p=0.008).

| **MOS-HIV PHS: Estimates** | | | | | | |  |
| --- | --- | --- | --- | --- | --- | --- | --- |
| Time | Group | Mean | Std. Error | df | 95% Confidence Interval | |  |
|  |  |  |  |  | Lower Bound | Upper Bound | Sig |
| Baseline | STR | 52,001 | 1,417 | 244,314 | 49,209 | 54,792 | ,750 |
|  | MTR | 52,469 | 1,196 | 215,057 | 50,111 | 54,827 | ,750 |
| Month 1 | STR | 55,423 | 1,455 | 274,649 | 52,558 | 58,287 | ,011 |
|  | MTR | 51,552 | 1,204 | 222,425 | 49,179 | 53,926 | ,011 |
| Month 3 | STR | 54,591 | 1,409 | 252,939 | 51,816 | 57,366 | ,004 |
|  | MTR | 50,402 | 1,192 | 216,204 | 48,052 | 52,752 | ,004 |
| Month 6 | STR | 55,650 | 1,407 | 259,450 | 52,879 | 58,421 | <0,001 |
|  | MTR | 50,144 | 1,196 | 220,957 | 47,786 | 52,501 | <0,001 |
| Month 12 | STR | 54,912 | 1,423 | 262,467 | 52,110 | 57,715 | <0,001 |
|  | MTR | 49,370 | 1,201 | 222,693 | 47,002 | 51,737 | <0,001 |
| Month 18 | STR | 55,912 | 1,472 | 256,895 | 53,013 | 58,811 | <0,001 |
|  | MTR | 49,351 | 1,228 | 231,421 | 46,931 | 51,771 | <0,001 |
| Month 24 | STR | 56,851 | 1,536 | 231,116 | 53,824 | 59,878 | <0,001 |
|  | MTR | 49,315 | 1,258 | 230,605 | 46,836 | 51,794 | <0,001 |

**MOS-HIV PHS: Graph**


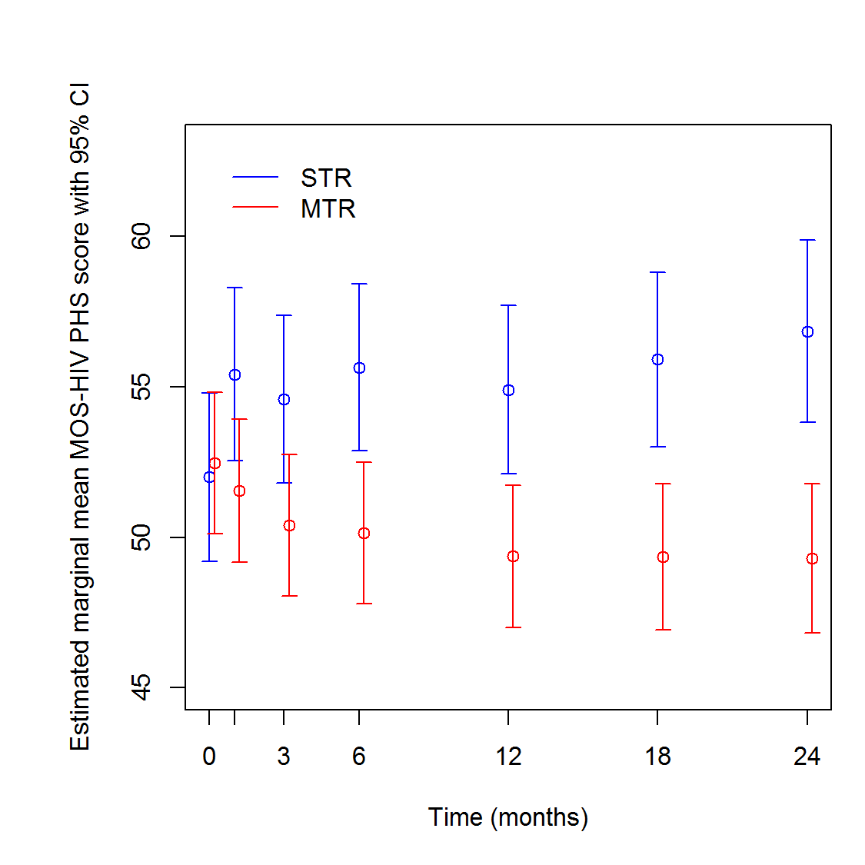


## Quality of Life: Mental health (Mental health score of MOS-HIV, standardized scale (T-score) with a mean of 50 and a standard deviation of 10 in the sample in which the summary scores were developed, higher score = better outcomes)

Model with random intercept, random slope, unstructured covariance structure and time continuous.
-2Restricted Log Likelihood: 7943.692
AIC: 7965.692
94.4% of data were available (1243/1316).

| **MOS-HIV MHS: Estimates of Fixed Effects** | | | | | | | |
| --- | --- | --- | --- | --- | --- | --- | --- |
| Parameter | Estimate | Std. Error | df | t | Sig. | 95% Confidence Interval | |
|  |  |  |  |  |  | Lower Bound | Upper Bound |
| Intercept | 47,277100 | ,926150 | 191,143 | 51,047 | ,000 | 45,450313 | 49,103887 |
| Time continuous | -,097177 | ,031418 | 173,640 | -3,093 | ,002 | -,159187 | -,035168 |
| STR group | 8,470520 | 1,588613 | 179,591 | 5,332 | ,000 | 5,335771 | 11,605269 |
| MTR group | 0 | 0 | . | . | . | . | . |
| STR * Time continuous | ,282121 | ,057931 | 175,773 | 4,870 | ,000 | ,167791 | ,396451 |
| MTR * Time continuous | 0 | 0 | . | . | . | . | . |
| Women | -,667319 | 2,068143 | 174,523 | -,323 | ,747 | -4,749109 | 3,414471 |
| Men | 0 | 0 | . | . | . | . | . |
| Non-Caucasian | 4,542892 | 2,457948 | 174,911 | 1,848 | ,066 | -,308161 | 9,393946 |
| Caucasian | 0 | 0 | . | . | . | . | . |
| Heterosexual | -1,151532 | 1,662130 | 174,760 | -,693 | ,489 | -4,431965 | 2,128900 |
| Homosexual | 0 | 0 | . | . | . | . | . |

Intercept: At 2y, MTR-, Caucasian, homosexual men have a MHS-score of 47.28 (p<0.001).

Time: Per month, MHS decreases by 0.10 in the MTR-group (p=0.002). *In the STR-group, MHS increases by 0.18 per month, p=0.001*

Stable: At 2y, the STR-group has a MHS-score of 8.47 more than the MTR-group (p<0.001).

Interaction: Per month, the estimated mean MHS score increases by 0.28 more in the STR-group than in the MTR-group (the 95% CI goes from 0.16 higher to 0.40 higher) (p<0.001).

Differences according to gender, ethnicity and sexual orientation are not significant.

|  | **MOS-HIV MHS: Estimates** | | | | | |  |
| --- | --- | --- | --- | --- | --- | --- | --- |
|  | StableART | Mean | Std. Error | df | 95% Confidence Interval | |  |
| Time |  |  |  |  | Lower Bound | Upper Bound | Sig |
| Baseline | STR | 52,671 | 1,408 | 186,453 | 49,894 | 55,448 | ,237 |
|  | MTR | 50,971 | 1,219 | 187,469 | 48,566 | 53,377 | ,237 |
| Month 1 | STR | 52,856 | 1,393 | 184,362 | 50,107 | 55,605 | ,162 |
|  | MTR | 50,874 | 1,212 | 184,999 | 48,482 | 53,266 | ,162 |
| Month 3 | STR | 53,226 | 1,369 | 180,588 | 50,524 | 55,927 | ,066 |
|  | MTR | 50,680 | 1,201 | 180,822 | 48,310 | 53,050 | ,066 |
| Month 6 | STR | 53,781 | 1,345 | 176,497 | 51,126 | 56,435 | ,012 |
|  | MTR | 50,388 | 1,190 | 176,673 | 48,040 | 52,737 | ,012 |
| Month 12 | STR | 54,890 | 1,344 | 176,202 | 52,238 | 57,543 | <0,001 |
|  | MTR | 49,805 | 1,190 | 176,723 | 47,457 | 52,154 | <0,001 |
| Month 18 | STR | 56,000 | 1,405 | 185,892 | 53,228 | 58,772 | <0,001 |
|  | MTR | 49,222 | 1,220 | 187,709 | 46,816 | 51,628 | <0,001 |
| Month 24 | STR | 57,110 | 1,521 | 199,265 | 54,110 | 60,109 | <0,001 |
|  | MTR | 48,639 | 1,277 | 206,990 | 46,122 | 51,156 | <0,001 |

**MOS-HIV MHS: Graph**


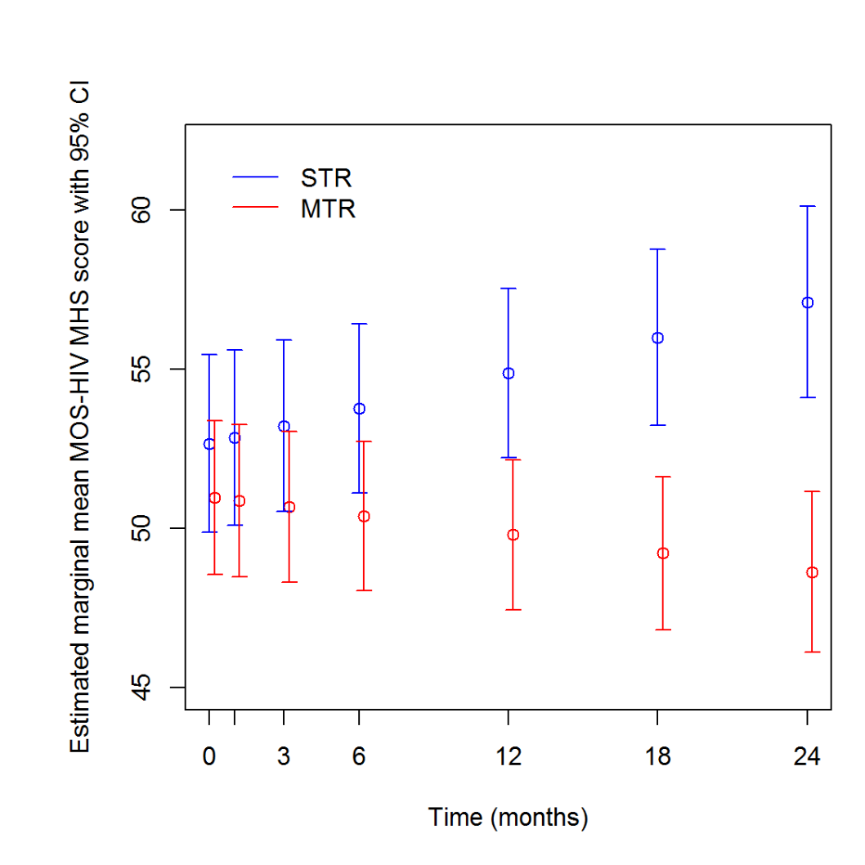


## Adherence: score (Case Adherence Index, range 0-16, higher score = better outcome)

Model with random intercept, random slope, unstructured covariance structure and time categorical.
-2Restricted Log Likelihood: 4354.774
AIC: 4396.774
94.1% of data were available (1239/1316).

| **CASE Adherence Index: Estimates of Fixed Effects** | | | | | | | |
| --- | --- | --- | --- | --- | --- | --- | --- |
| Parameter | Estimate | Std. Error | df | t | Sig. | 95% Confidence Interval | |
|  |  |  |  |  |  | Lower Bound | Upper Bound |
| Intercept | 13,854425 | ,204653 | 240,809 | 67,697 | ,000 | 13,451287 | 14,257564 |
| Baseline | ,658345 | ,204670 | 288,557 | 3,217 | ,001 | ,255509 | 1,061181 |
| Month 1 | ,409745 | ,204780 | 334,773 | 2,001 | ,046 | ,006928 | ,812562 |
| Month 3 | ,493536 | ,195130 | 389,624 | 2,529 | ,012 | ,109895 | ,877176 |
| Month 6 | ,278899 | ,186700 | 544,935 | 1,494 | ,136 | -,087840 | ,645638 |
| Month 12 | ,385047 | ,168781 | 917,745 | 2,281 | ,023 | ,053804 | ,716289 |
| Month 18 | ,203065 | ,159976 | 932,727 | 1,269 | ,205 | -,110890 | ,517020 |
| Month 24 | 0 | 0 | . | . | . | . | . |
| STR group | 1,576049 | ,355624 | 230,302 | 4,432 | ,000 | ,875357 | 2,276741 |
| MTR group | 0 | 0 | . | . | . | . | . |
| Baseline * STR group | -2,054572 | ,375757 | 287,615 | -5,468 | ,000 | -2,794154 | -1,314989 |
| Baseline * MTR group | 0 | 0 | . | . | . | . | . |
| Month 1 * STR group | -1,123534 | ,383179 | 354,063 | -2,932 | ,004 | -1,877127 | -,369940 |
| Month 1 * MTR group | 0 | 0 | . | . | . | . | . |
| Month 3 * STR group | -1,023414 | ,361261 | 398,379 | -2,833 | ,005 | -1,733629 | -,313198 |
| Month 3 * MTR group | 0 | 0 | . | . | . | . | . |
| Month 6 * STR group | -,835398 | ,340545 | 533,646 | -2,453 | ,014 | -1,504370 | -,166425 |
| Month 6 * MTR group | 0 | 0 | . | . | . | . | . |
| Month 12 * STR group | -,640665 | ,311377 | 920,653 | -2,058 | ,040 | -1,251755 | -,029574 |
| Month 12 * MTR group | 0 | 0 | . | . | . | . | . |
| Month 18 * STR group | -,670798 | ,291873 | 932,284 | -2,298 | ,022 | -1,243602 | -,097994 |
| Month 18 * MTR group | 0 | 0 | . | . | . | . | . |
| Month 24 * STR group | 0 | 0 | . | . | . | . | . |
| Month 24 * MTR group | 0 | 0 | . | . | . | . | . |
| Women | ,051721 | ,414479 | 173,916 | ,125 | ,901 | -,766335 | ,869777 |
| Men | 0 | 0 | . | . | . | . | . |
| Non-Caucasian | -,204021 | ,493036 | 174,859 | -,414 | ,680 | -1,177088 | ,769046 |
| Caucasian | 0 | 0 | . | . | . | . | . |
| Heterosexual | -,066907 | ,333050 | 174,017 | -,201 | ,841 | -,724245 | ,590430 |
| Homosexual | 0 | 0 | . | . | . | . | . |

Intercept: At 2y, MTR-, Caucasian, homosexual men have a CASE sumscore of 13.85 (p<0.001).

Time:
Within the MTR-group, at baseline there is a 0.66 higher score as compared to 2y (p=0.001) at month 1, a 0.41 higher score (p=0.046), at month 3 a 0.49 higher score (p=0.012) and at 1y a 0.39 higher score (p=0.023)

*A second model (in which 2y+STR-group are the reference groups) shows a significant different score within the STR-group at baseline (1.40 lower, p<0.001) and at month 1 (0.71 lower score, p=0.028), as compared to 2y.*

*A third model (in which baseline+MTR are the reference groups) shows significantly different scores within the MTR-group at month 6 (0.38 lower, p=0.018), at month 18 (0.46 lower, p=0.015) and at 2y (0.66 lower, p=0.001) as compared to baseline.*

*A fourth model (in which baseline+STR are the reference groups) shows only a significant difference within the STR-group as compared to baseline at month 1 (0.68 higher, p=0.007) month 3 (0.87 higher, p<0.001), at month 6 (0.84 higher, p=0.001), at 1y (1.14 higher, p<0.001), at month 18 (0.93 higher, p=0.001) and at 2y (1.40 higher, p<0.001).*

Stable: At 2y, the STR-group has a CASE sumscore which is 1.58 higher than the MTR-group (p<0.01).

Interactie: The mean estimated difference in CASE SUM score between baseline and 2y between the groups is 2.05 (p<0.001)
- STR-group increased 1.40 gestegen, MTR-group decreased 0.66 over time
- STR-group hadden 0.48 lower score at baseline and have a 1.58 higher score at 2y

Differences according to gender, ethnicity and sexual orientation are not significant.

| **CASE Adherence Index: Estimates** | | | | | | |  |
| --- | --- | --- | --- | --- | --- | --- | --- |
| Time | Group | Mean | Std. Error | df | 95% Confidence Interval | |  |
|  |  |  |  |  | Lower Bound | Upper Bound | Sig |
| Baseline | STR | 13,925 | ,320 | 280,594 | 13,295 | 14,554 | ,159 |
|  | MTR | 14,403 | ,264 | 246,358 | 13,884 | 14,923 | ,159 |
| Month 1 | STR | 14,607 | ,332 | 331,669 | 13,954 | 15,260 | ,199 |
|  | MTR | 14,155 | ,266 | 257,701 | 13,631 | 14,678 | ,199 |
| Month 3 | STR | 14,791 | ,320 | 312,841 | 14,161 | 15,421 | ,101 |
|  | MTR | 14,238 | ,262 | 247,197 | 13,723 | 14,754 | ,101 |
| Month 6 | STR | 14,764 | ,312 | 305,237 | 14,150 | 15,379 | ,025 |
|  | MTR | 14,024 | ,261 | 249,659 | 13,510 | 14,538 | ,025 |
| Month 12 | STR | 15,065 | ,313 | 311,150 | 14,449 | 15,681 | ,005 |
|  | MTR | 14,130 | ,260 | 244,939 | 13,619 | 14,641 | ,005 |
| Month 18 | STR | 14,853 | ,320 | 292,271 | 14,223 | 15,483 | ,008 |
|  | MTR | 13,948 | ,265 | 251,723 | 13,427 | 14,469 | ,008 |
| Month 24 | STR | 15,321 | ,333 | 246,514 | 14,666 | 15,976 | <0,001 |
|  | MTR | 13,745 | ,270 | 242,526 | 13,213 | 14,277 | <0,001 |
|  | | | | | | |  |

**CASE Adherence Index: Graph**


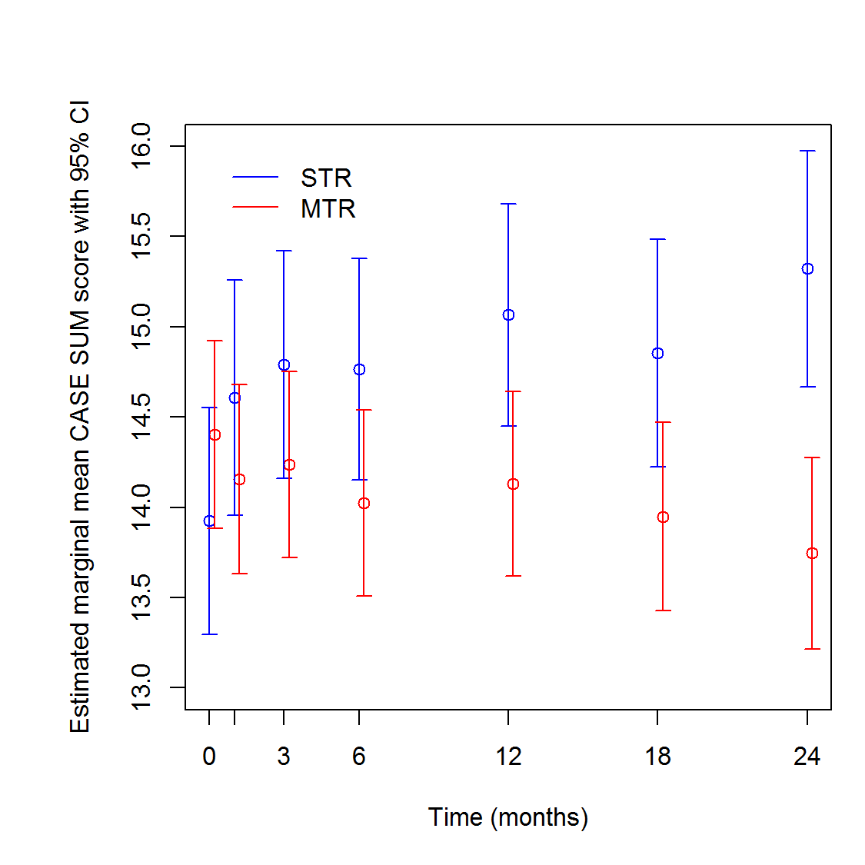


## Adherence: VAS (Visual Analogue Scale, range 0-100, higher score = better outcome)

Model with random intercept, random slope, unstructured covariance structure and time categorical.
-2Restricted Log Likelihood: 7428.896
AIC: 7470.896
94.3% of data were available (1241/1316).

| **Adherence VAS: Estimates of Fixed Effects** | | | | | | | |
| --- | --- | --- | --- | --- | --- | --- | --- |
| Parameter | Estimate | Std. Error | df | t | Sig. | 95% Confidence Interval | |
|  |  |  |  |  |  | Lower Bound | Upper Bound |
| Intercept | 95,173380 | ,581482 | 298,469 | 163,674 | ,000 | 94,029056 | 96,317705 |
| Baseline | 1,972063 | ,697246 | 354,816 | 2,828 | ,005 | ,600810 | 3,343317 |
| Month 1 | ,781916 | ,709259 | 426,136 | 1,102 | ,271 | -,612166 | 2,175998 |
| Month 3 | 1,587355 | ,685734 | 512,141 | 2,315 | ,021 | ,240157 | 2,934552 |
| Month 6 | 1,182444 | ,675500 | 716,866 | 1,750 | ,080 | -,143750 | 2,508639 |
| Month 12 | ,729158 | ,643374 | 985,112 | 1,133 | ,257 | -,533382 | 1,991699 |
| Month 18 | ,238147 | ,629864 | 902,732 | ,378 | ,705 | -,998022 | 1,474315 |
| Month 24 | 0 | 0 | . | . | . | . | . |
| STR group | 4,142066 | 1,014690 | 295,796 | 4,082 | ,000 | 2,145140 | 6,138992 |
| MTR group | 0 | 0 | . | . | . | . | . |
| Baseline * STR group | -6,273950 | 1,281369 | 354,816 | -4,896 | ,000 | -8,793984 | -3,753916 |
| Baseline * MTR group | 0 | 0 | . | . | . | . | . |
| Month 1 * STR group | -2,684552 | 1,327798 | 447,254 | -2,022 | ,044 | -5,294049 | -,075054 |
| Month 1 * MTR group | 0 | 0 | . | . | . | . | . |
| Month 3 * STR group | -2,691203 | 1,266910 | 519,045 | -2,124 | ,034 | -5,180105 | -,202301 |
| Month 3 * MTR group | 0 | 0 | . | . | . | . | . |
| Month 6 * STR group | -1,474965 | 1,229852 | 703,775 | -1,199 | ,231 | -3,889583 | ,939653 |
| Month 6 * MTR group | 0 | 0 | . | . | . | . | . |
| Month 12 * STR group | -,005849 | 1,185369 | 985,900 | -,005 | ,996 | -2,331985 | 2,320287 |
| Month 12 * MTR group | 0 | 0 | . | . | . | . | . |
| Month 18 * STR group | -,387860 | 1,156567 | 902,335 | -,335 | ,737 | -2,657734 | 1,882014 |
| Month 18 * MTR group | 0 | 0 | . | . | . | . | . |
| Month 24 * STR group | 0 | 0 | . | . | . | . | . |
| Month 24 * MTR group | 0 | 0 | . | . | . | . | . |
| Women | -,561539 | 1,136627 | 171,426 | -,494 | ,622 | -2,805127 | 1,682048 |
| Men | 0 | 0 | . | . | . | . | . |
| Non-Caucasian | -2,796892 | 1,350410 | 171,521 | -2,071 | ,040 | -5,462454 | -,131329 |
| Caucasian | 0 | 0 | . | . | . | . | . |
| Heterosexual | -,539334 | ,911709 | 170,345 | -,592 | ,555 | -2,339037 | 1,260369 |
| Homosexual | 0 | 0 | . | . | . | . | . |

Intercept: AT 2y, MTR, Caucasian, homosexual men have a score of 95.17 (p<0.001).

Time:

Within the MTR-group, there are no significant different scores at baseline (1.97 higher, p=0.005) and at month 3 (1.59 higher, p=0.021) as compared to 2y.

*A second model (in which 2y+STR-group are the reference groups) shows a significant different score within the STR-group at baseline (4.30 lower, p<0.001) as compared to 2y*

*A third model (in which baseline+MTR are the reference groups) shows significantly different scores within the MTR-group at month 18 (1.73 lower, p=0.010) and at 2y (1.97 lower, p=0.005) as compared to baseline.*

*A fourth model (in which baseline+STR are the reference groups) shows only a significant difference within the STR-group as compared to baseline at
month 1 (2.40 higher, p=0.017)
month 3 (3.20 higher, p=0.001)
month 6 (4.00 higher, p<0.001)
1y (5.03 higher, p<0.001)
month 18 (4.15 higher, p<0.001)
2y ( 4.30 higher, p<0.001)*

Stable: At 2y, STR-patients have a 4.14 higher score as compared to MTR-patients (p<0.001)

Interaction: The estimated mean difference between baseline and 2y between the groups is 6.27 (p<0.001):
- VAS-score in the STR-group increased by 4.30, VAS-score in the MTR-group decreased by 1.97 over time.
- The STR-group had a 2.13 lower score at baseline and 4.14 higher at 2y.

Differences in ethnicity are significant: at 2y, non-Caucasian participants have 2.80 points less than Caucasian participants (p=0.040).

| **Adherence VAS: Estimates** | | | | | | |  |
| --- | --- | --- | --- | --- | --- | --- | --- |
| Time | Group | Mean | Std. Error | df | 95% Confidence Interval | |  |
|  |  |  |  |  | Lower Bound | Upper Bound | Sig |
| Baseline | STR | 93,065 | 1,030 | 371,572 | 91,040 | 95,089 | ,060 |
|  | MTR | 95,197 | ,802 | 323,981 | 93,618 | 96,775 | ,060 |
| Month 1 | STR | 95,464 | 1,079 | 452,548 | 93,343 | 97,585 | ,219 |
|  | MTR | 94,006 | ,815 | 352,526 | 92,404 | 95,609 | ,219 |
| Month 3 | STR | 96,263 | 1,026 | 439,495 | 94,247 | 98,279 | ,196 |
|  | MTR | 94,812 | ,796 | 339,026 | 93,246 | 96,378 | ,196 |
| Month 6 | STR | 97,074 | ,992 | 450,376 | 95,125 | 99,023 | ,015 |
|  | MTR | 94,407 | ,791 | 345,248 | 92,852 | 95,962 | ,015 |
| Month 12 | STR | 98,090 | ,973 | 472,705 | 96,178 | 100,002 | <0,001 |
|  | MTR | 93,954 | ,771 | 325,460 | 92,437 | 95,470 | <0,001 |
| Month 18 | STR | 97,217 | ,961 | 420,245 | 95,327 | 99,106 | <0,001 |
|  | MTR | 93,463 | ,767 | 310,855 | 91,953 | 94,972 | <0,001 |
| Month 24 | STR | 97,367 | ,942 | 296,875 | 95,514 | 99,220 | <0,001 |
|  | MTR | 93,224 | ,756 | 266,680 | 91,737 | 94,712 | <0,001 |
| a. Dependent Variable: CASEVAS.1: CASE VAS. | | | | | | |  |

**Adherence VAS: Graph**


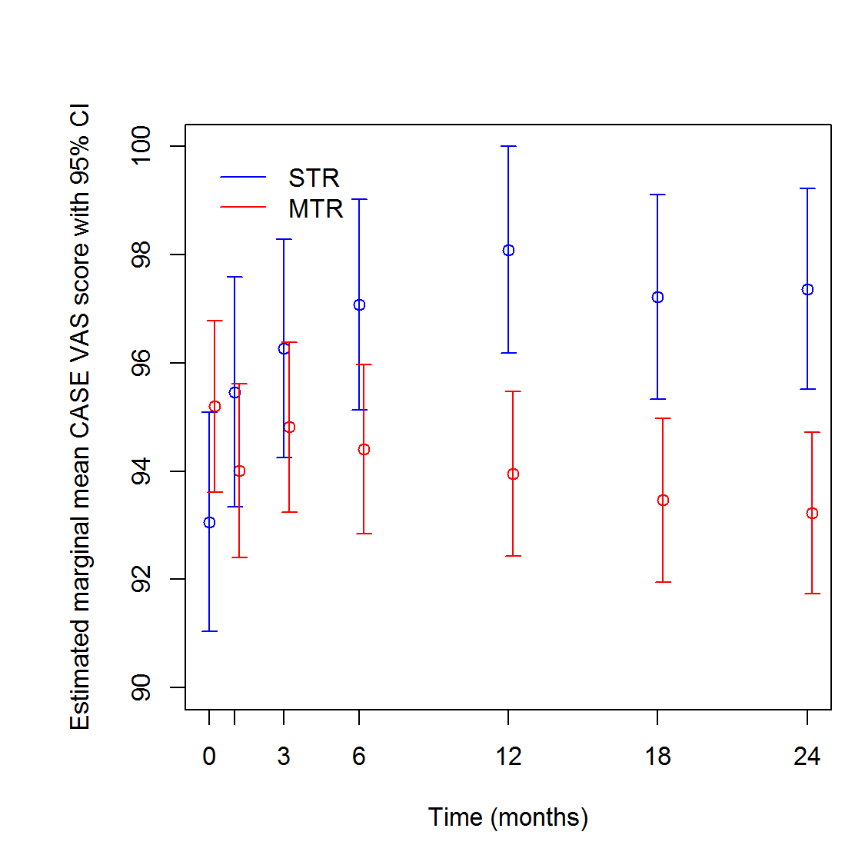


## Treatment satisfaction (HIV Treatment Satisfaction Questionnaire, 0-60, higher score = better outcome)

Model with random intercept, random slope, unstructured covariance structure and time continuous.
-2Restricted Log Likelihood: 4078.748
AIC: 4100.748

94.9% of data were available 714/752) (188*4 = 752)

| **Treatment Satisfaction: Estimates of Fixed Effects** | | | | | | | |
| --- | --- | --- | --- | --- | --- | --- | --- |
| Parameter | Estimate | Std. Error | df | t | Sig. | 95% Confidence Interval | |
|  |  |  |  |  |  | Lower Bound | Upper Bound |
| Intercept | 54,293673 | ,713986 | 208,171 | 76,043 | ,000 | 52,886103 | 55,701242 |
| Baseline | ,151972 | ,553642 | 326,739 | ,274 | ,784 | -,937182 | 1,241125 |
| Month 12 | ,108488 | ,564384 | 327,739 | ,192 | ,848 | -1,001784 | 1,218760 |
| Month 18 | -,148668 | ,581062 | 329,203 | -,256 | ,798 | -1,291732 | ,994396 |
| Month 24 | 0 | 0 | . | . | . | . | . |
| STR group | 3,137894 | 1,350935 | 241,464 | 2,323 | ,021 | ,476772 | 5,799017 |
| MTR group | 0 | 0 | . | . | . | . | . |
| Baseline * STR group | -3,496733 | 1,169928 | 327,671 | -2,989 | ,003 | -5,798250 | -1,195216 |
| Baseline * MTR group | 0 | 0 | . | . | . | . | . |
| Month 12 * STR group | -2,553884 | 1,193695 | 332,121 | -2,139 | ,033 | -4,902039 | -,205729 |
| Month 12 * MTR group | 0 | 0 | . | . | . | . | . |
| Month 18 * STR group | -2,516232 | 1,194444 | 328,912 | -2,107 | ,036 | -4,865946 | -,166518 |
| Month 18 * MTR group | 0 | 0 | . | . | . | . | . |
| Month 24 * STR group | 0 | 0 | . | . | . | . | . |
| Month 24 * MTR group | 0 | 0 | . | . | . | . | . |
| Women | ,128171 | 1,618611 | 123,057 | ,079 | ,937 | -3,075755 | 3,332097 |
| Men | 0 | 0 | . | . | . | . | . |
| Non-Caucasian | ,024943 | 2,209635 | 129,316 | ,011 | ,991 | -4,346773 | 4,396659 |
| Caucasian | 0 | 0 | . | . | . | . | . |
| Heterosexual | -1,036714 | 1,300589 | 122,110 | -,797 | ,427 | -3,611337 | 1,537909 |
| Homosexual | 0 | 0 | . | . | . | . | . |

Intercept: AT 2y, MTR, Caucasian, homosexual men have a score of 51.63 (p<0.001).

Time: Per month, the score decreases by 0.08 in MTR-group (p<0.001). Per month, the score increases by 0.18 in the STR-group (p<0.001).

Stable: At 2y, STR-patients have a 6.80 higher score as compared to MTR-patients (p<0.001).

Interaction: The estimated mean increase in satisfaction score per month is 0.27 higher under STR than under STR (the 95% CI goes from 0.19 higher to 0.36 higher (p<0.001).

Differences according to gender, ethnicity and sexual orientation are not significant.

| **Treatment Satisfaction: Estimates** | | | | | | |  |
| --- | --- | --- | --- | --- | --- | --- | --- |
| Time | Group | Mean | Std. Error | df | 95% Confidence Interval | |  |
|  |  |  |  |  | Lower Bound | Upper Bound | Sig |
| Baseline | STR | 54,488 | ,959 | 207,675 | 52,597 | 56,378 | ,786 |
|  | MTR | 54,265 | ,791 | 214,659 | 52,706 | 55,824 | ,786 |
| Month 12 | STR | 56,683 | ,826 | 178,389 | 55,053 | 58,313 | ,659 |
|  | MTR | 53,174 | ,733 | 179,476 | 51,726 | 54,621 | ,659 |
| Month 18 | STR | 57,781 | ,845 | 181,839 | 56,113 | 59,449 | ,644 |
|  | MTR | 52,628 | ,744 | 185,007 | 51,160 | 54,096 | ,644 |
| Month 24 | STR | 58,878 | ,921 | 197,323 | 57,063 | 60,694 | ,021 |
|  | MTR | 52,082 | ,780 | 204,925 | 50,544 | 53,621 | ,021 |

**Treatment Satisfaction: Graph**


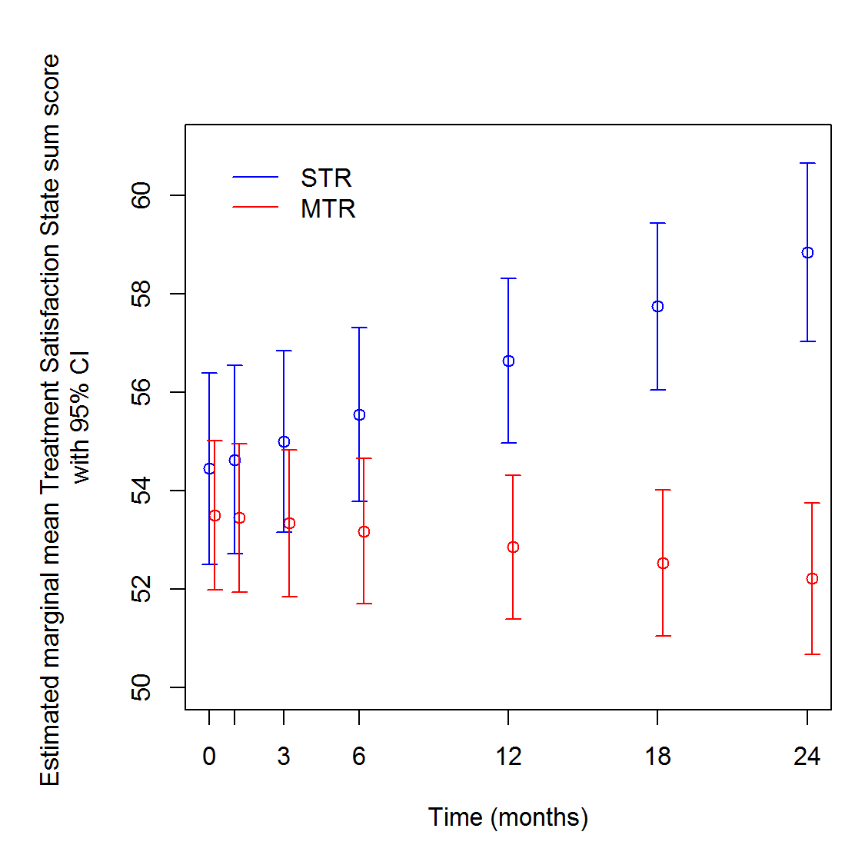


## Presence of neurocognitive complaints (Neurocognitive screening questions, yes/no, yes = worse outcome)

Generalized estimating equations model with exchangeable correlation matrix and time continuous.
QIC: 1624.119
QICC: 1599.554
94.5% of data were available (1244/1316).

| **Neurocognitive complaints: Parameter Estimates** | | | | | | | | | | |
| --- | --- | --- | --- | --- | --- | --- | --- | --- | --- | --- |
| Parameter | B | Std. Error | 95% Wald Confidence Interval | | Hypothesis Test | | | Exp(B) | 95% Wald Confidence Interval for Exp(B) | |
|  |  |  | Lower | Upper | Wald Chi-Square | df | Sig. |  | Lower | Upper |
| Intercept | ,667 | ,2000 | ,275 | 1,059 | 11,114 | 1 | ,001 | 1,948 | 1,316 | 2,882 |
| Time continuous | ,025 | ,0072 | ,010 | ,039 | 11,629 | 1 | ,001 | 1,025 | 1,011 | 1,040 |
| STR group | -1,029 | ,3281 | -1,672 | -,385 | 9,826 | 1 | ,002 | ,358 | ,188 | ,680 |
| MTR group | 0 | . | . | . | . | . | . | 1 | . | . |
| STR * Time continuous | -,029 | ,0153 | -,059 | ,001 | 3,531 | 1 | ,060 | ,972 | ,943 | 1,001 |
| MTR * Time continuous | 0 | . | . | . | . | . | . | 1 | . | . |
| Women | ,127 | ,4016 | -,660 | ,914 | ,100 | 1 | ,752 | 1,136 | ,517 | 2,495 |
| Men | 0 | . | . | . | . | . | . | 1 | . | . |
| Non-Caucasian | -,850 | ,4349 | -1,703 | ,002 | 3,824 | 1 | ,051 | ,427 | ,182 | 1,002 |
| Caucasian | 0 | . | . | . | . | . | . | 1 | . | . |
| Heterosexual | -,148 | ,3295 | -,794 | ,497 | ,202 | 1 | ,653 | ,862 | ,452 | 1,645 |
| Homosexual | 0 | . | . | . | . | . | . | 1 | . | . |
| (Scale) | 1 |  |  |  |  |  |  |  |  |  |

Intercept: Odds on NCC in MTR-group is 1.948 (p=0.001)
Time: ODDS of having NCC increases by 2.5% in stable group (p=0.002)

Stable: At 2y: STR-groups has ODDS of 0.358 of having NCC as compared to MTR-group

Interaction: As time increases 1 month, ODDS of having NCC decreases with 2.9% among switch patients as compared to stable group (p=0.060). Other formulation: as time increases 1 month, ODDS of having NCC increases with 2.9% as compared to STR-group

|  | **Neurocognitive complaints: Estimates** | | | | |  |
| --- | --- | --- | --- | --- | --- | --- |
|  | Group | Mean | Std. Error | 95% Wald Confidence Interval | |  |
| Time |  |  |  | Lower | Upper | Sig |
| Baseline | STR | ,33 | ,062 | ,22 | ,46 | ,243 |
|  | MTR | ,41 | ,053 | ,31 | ,52 | ,243 |
| Month 1 | STR | ,33 | ,060 | ,22 | ,46 | ,193 |
|  | MTR | ,42 | ,053 | ,32 | ,52 | ,193 |
| Month 3 | STR | ,33 | ,057 | ,23 | ,45 | ,114 |
|  | MTR | ,43 | ,052 | ,33 | ,53 | ,114 |
| Month 6 | STR | ,33 | ,053 | ,23 | ,44 | ,045 |
|  | MTR | ,45 | ,053 | ,35 | ,55 | ,045 |
| Month 12 | STR | ,32 | ,049 | ,23 | ,42 | ,006 |
|  | MTR | ,48 | ,054 | ,38 | ,59 | ,006 |
| Month 18 | STR | ,32 | ,052 | ,22 | ,42 | ,002 |
|  | MTR | ,52 | ,057 | ,41 | ,63 | ,002 |
| Month 24 | STR | ,31 | ,059 | ,21 | ,44 | ,001 |
|  | MTR | ,56 | ,061 | ,44 | ,67 | ,001 |
|  |  | | | | |  |
|  |  | | | | |  |

**Neurocognitive complaints: Graph**


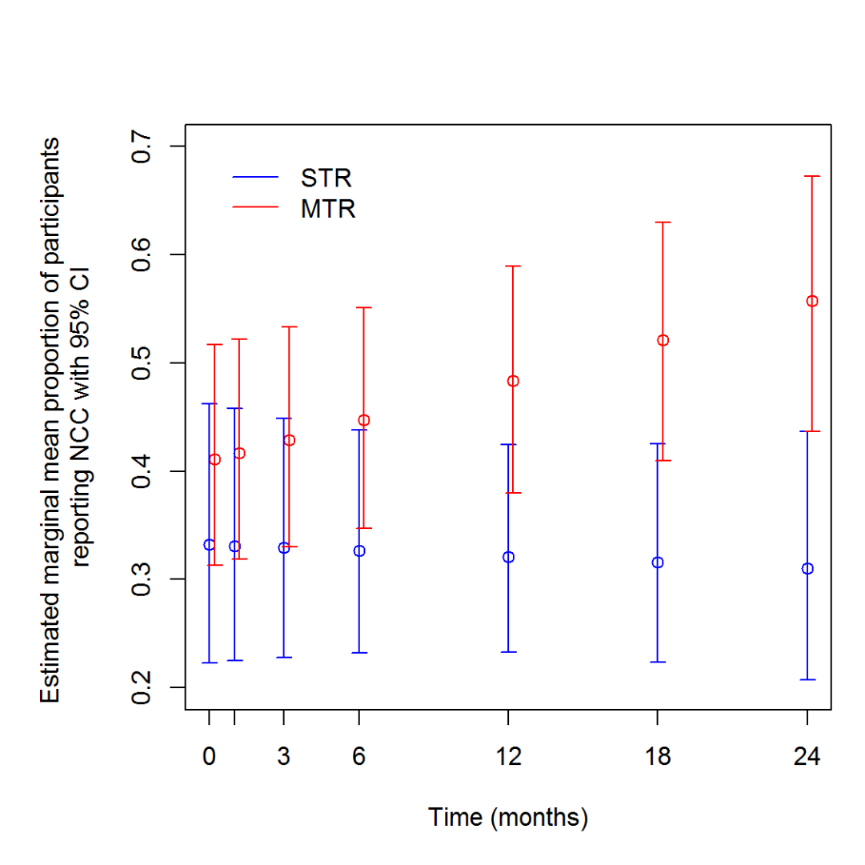


# **Mixed models for additional file: Worstbest scenario**

Missing lost-to-follow-up data were filled with bad outcomes for STR-group and good outcomes for MTR-group.

## Symptoms (HIV Symptoms Index, range 0-20, more symptoms = worse outcome)

Model with random intercept, random slope, unstructured covariance structure and time continuous.
-2Restricted Log Likelihood: 6116.405
Akaike’s Information Criterion (AIC): 6124.405
94.8% of data were available (1247/1316)

| **Symptoms: Estimates of Fixed Effects** | | | | | | | |
| --- | --- | --- | --- | --- | --- | --- | --- |
| Parameter | Estimate | Std. Error | df | t | Sig. | 95% Confidence Interval | |
|  |  |  |  |  |  | Lower Bound | Upper Bound |
| Intercept | 3,280722 | ,386632 | 186,234 | 8,485 | ,000 | 2,517980 | 4,043464 |
| Time continuous | -,036017 | ,013300 | 170,026 | -2,708 | ,007 | -,062271 | -,009763 |
| STR group | 3,470681 | ,662147 | 174,719 | 5,242 | ,000 | 2,163844 | 4,777518 |
| MTR group | 0 | 0 | . | . | . | . | . |
| STR * Time continuous | ,088400 | ,024532 | 172,175 | 3,604 | ,000 | ,039979 | ,136822 |
| MTR * Time continuous | 0 | 0 | . | . | . | . | . |
| Women | ,152835 | ,870918 | 173,581 | ,175 | ,861 | -1,566118 | 1,871789 |
| Men | 0 | 0 | . | . | . | . | . |
| Non-Caucasian | -,042872 | 1,035520 | 174,247 | -,041 | ,967 | -2,086649 | 2,000904 |
| Caucasian | 0 | 0 | . | . | . | . | . |
| Heterosexual | -,102014 | ,699856 | 173,730 | -,146 | ,884 | -1,483329 | 1,279302 |
| Homosexual | 0 | 0 | . | . | . | . | . |

Intercept: At 2y MTR-, Caucasian, homosexual men have 3.28 symptoms (p<0.001).

Time: Per month, the number of symptoms decreases by 0.04 in the MTR-group (p=0.007).
*In STR-group, number of symptoms increases by 0.05 (p=0.012).*

Stable: At 2y, the STR-group has 3.47 symptoms more than the MTR-group (p<0.001)

Interaction: The estimated mean increase in symptoms per month is 0.09 higher among STR-group as compared to MTR-group (the 95% CI goes from 0.04 to 0.14 higher), p<0.001.

No differences according to gender, ethnicity and sexual orientation.

| **Symptoms: Estimates** | | | | | | |  |
| --- | --- | --- | --- | --- | --- | --- | --- |
| Time | Group | Mean | Std. Error | Df | 95% Confidence Interval | |  |
|  |  |  |  |  | Lower Bound | Upper Bound | Sig |
| Baseline | STR | 5,498 | ,598 | 187,671 | 4,319 | 6,677 | ,028 |
|  | MTR | 4,149 | ,516 | 188,660 | 3,132 | 5,167 | ,028 |
| Month 1 | STR | 5,551 | ,591 | 185,498 | 4,384 | 6,717 | ,018 |
|  | MTR | 4,113 | ,513 | 186,045 | 3,102 | 5,124 | ,018 |
| Month 3 | STR | 5,655 | ,580 | 181,471 | 4,511 | 6,799 | ,006 |
|  | MTR | 4,041 | ,507 | 181,537 | 3,040 | 5,042 | ,006 |
| Month 6 | STR | 5,812 | ,568 | 176,796 | 4,691 | 6,934 | ,001 |
|  | MTR | 3,933 | ,502 | 176,812 | 2,942 | 4,924 | ,001 |
| Month 12 | STR | 6,127 | ,565 | 174,820 | 5,011 | 7,242 | <0,001 |
|  | MTR | 3,717 | ,501 | 175,526 | 2,728 | 4,705 | <0,001 |
| Month 18 | STR | 6,441 | ,588 | 182,587 | 5,280 | 7,602 | <0,001 |
|  | MTR | 3,501 | ,512 | 185,019 | 2,490 | 4,511 | <0,001 |
| Month 24 | STR | 6,755 | ,636 | 194,200 | 5,502 | 8,009 | <0,001 |
|  | MTR | 3,285 | ,535 | 202,714 | 2,229 | 4,340 | <0,001 |
|  | | | | | | |  |

**Symptoms: Graph**


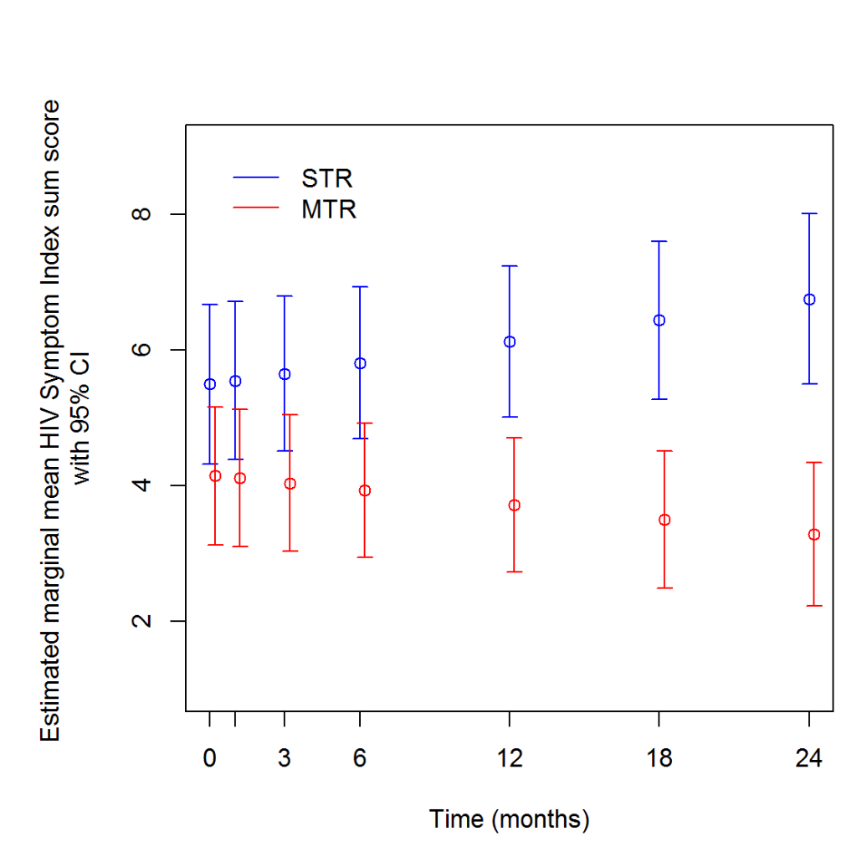


## Depressive symptoms (Beck Depression Inventory II, range 0-63, higher score = worse outcome)

Model with random intercept, random slope, unstructured covariance structure and time categorical.
-2Restricted Log Likelihood: 7669.836
AIC: 7677.836
94.7% of data were available (1246/1316)

| **BDI: Estimates of Fixed Effects** | | | | | | | |
| --- | --- | --- | --- | --- | --- | --- | --- |
| Parameter | Estimate | Std. Error | df | t | Sig. | 95% Confidence Interval | |
|  |  |  |  |  |  | Lower Bound | Upper Bound |
| Intercept | 5,339656 | ,924738 | 224,156 | 5,774 | ,000 | 3,517365 | 7,161947 |
| Baseline | 4,142197 | ,788853 | 293,326 | 5,251 | ,000 | 2,589668 | 5,694726 |
| Month 1 | 2,831178 | ,790849 | 342,328 | 3,580 | ,000 | 1,275642 | 4,386713 |
| Month 3 | 1,720501 | ,752453 | 396,776 | 2,287 | ,023 | ,241209 | 3,199793 |
| Month 6 | ,831123 | ,725220 | 564,860 | 1,146 | ,252 | -,593334 | 2,255581 |
| Month 12 | ,850628 | ,660358 | 936,685 | 1,288 | ,198 | -,445323 | 2,146580 |
| Month 18 | 1,204062 | ,627998 | 932,454 | 1,917 | ,056 | -,028392 | 2,436516 |
| Month 24 | 0 | 0 | . | . | . | . | . |
| STR group | 8,782832 | 1,590292 | 217,272 | 5,523 | ,000 | 5,648459 | 11,917205 |
| MTR group | 0 | 0 | . | . | . | . | . |
| Baseline * STR group | -8,884715 | 1,452173 | 294,666 | -6,118 | ,000 | -11,742659 | -6,026771 |
| Baseline * MTR group | 0 | 0 | . | . | . | . | . |
| Month 1 * STR group | -5,241656 | 1,480693 | 362,449 | -3,540 | ,000 | -8,153484 | -2,329828 |
| Month 1 * MTR group | 0 | 0 | . | . | . | . | . |
| Month 3 * STR group | -3,345765 | 1,383986 | 397,905 | -2,417 | ,016 | -6,066604 | -,624925 |
| Month 3 * MTR group | 0 | 0 | . | . | . | . | . |
| Month 6 * STR group | -2,303640 | 1,322224 | 552,972 | -1,742 | ,082 | -4,900837 | ,293557 |
| Month 6 * MTR group | 0 | 0 | . | . | . | . | . |
| Month 12 * STR group | ,208907 | 1,213698 | 936,814 | ,172 | ,863 | -2,172974 | 2,590788 |
| Month 12 * MTR group | 0 | 0 | . | . | . | . | . |
| Month 18 * STR group | ,235211 | 1,147691 | 931,917 | ,205 | ,838 | -2,017147 | 2,487570 |
| Month 18 * MTR group | 0 | 0 | . | . | . | . | . |
| Month 24 * STR group | 0 | 0 | . | . | . | . | . |
| Month 24 * MTR group | 0 | 0 | . | . | . | . | . |
| Women | ,196586 | 2,028881 | 174,584 | ,097 | ,923 | -3,807705 | 4,200876 |
| Men | 0 | 0 | . | . | . | . | . |
| Non-Caucasian | -1,760577 | 2,411518 | 175,025 | -,730 | ,466 | -6,519974 | 2,998820 |
| Caucasian | 0 | 0 | . | . | . | . | . |
| Heterosexual | ,335110 | 1,630068 | 174,615 | ,206 | ,837 | -2,882061 | 3,552282 |
| Homosexual | 0 | 0 | . | . | . | . | . |

Intercept: At 2y MTR-, Caucasian, homosexual men have 5.34 depressive symptoms (p<0.001).

Time:

Within the MTR-group:
at baseline 4.14 higher than at 2y (p<0.001)
at T1 2.83 higher than at 2y (p<0.001)
at T2 1.72 higher than at 2y (p=0.023)

*A second model (in which 2y+STR-group are the reference groups) shows significant different scores within the STR-group as compared to 2y:*at baseline 4.74 less than at 2y (<0.001)

*A third model (in which baseline+MTR are the reference groups) shows significantly different scores within the MTR-group as compared to baseline:*at T1 1.31 more than at baseline (p=0.035)
at T2 2.42 more than at baseline (p<0.001)
at T3 3.31 more than at baseline (p<0.001)
at T4 3.29 more than at baseline (p<0.001)
at T5 2.94 more than at baseline (p<0.001)
at T6 4.14 more than at baseline (p<0.001)

*A fourth model (in which baseline+STR are the reference groups) shows significant differences within the STR-group as compared to baseline:*at T1 2.33 more than at baseline (0.020)
at T2 3.11 more than at baseline (p=0.001)
at T3 3.27 more than at baseline (p=0.001)
at T4 5.80 more than at baseline (p<0.001)
at T5 6.18 more than at baseline (p<0.001)
at T6 4.74 more than at baseline (p<0.001)

Stable: At 2y, the STR-group has 8.78 depressive symptoms more than the MTR-group (p<0.001)

Interaction: The estimated mean difference over time between the groups is 8.88 (p<0.001)
STR-group score increased by 4.74, MTR-group score decreased by 4.14 over time
STR-group score at baseline was 0.10 lower and at 2y the score was 8.78 higher.

Differences according to gender, ethnicity and sexual orientation are not significant.

| **BDI: Estimates** | | | | | | |  |
| --- | --- | --- | --- | --- | --- | --- | --- |
| Time | Group | Mean | Std. Error | df | 95% Confidence Interval | |  |
|  |  |  |  |  | Lower Bound | Upper Bound | Sig |
| Baseline | STR | 8,766 | 1,483 | 247,564 | 5,845 | 11,686 | ,947 |
|  | MTR | 8,867 | 1,245 | 219,920 | 6,413 | 11,321 | ,947 |
| Month 1 | STR | 11,098 | 1,519 | 276,773 | 8,108 | 14,087 | ,026 |
|  | MTR | 7,556 | 1,253 | 227,213 | 5,088 | 10,025 | ,026 |
| Month 3 | STR | 11,883 | 1,465 | 252,785 | 8,998 | 14,768 | <0,001 |
|  | MTR | 6,446 | 1,238 | 219,720 | 4,006 | 8,886 | <0,001 |
| Month 6 | STR | 12,036 | 1,455 | 257,395 | 9,171 | 14,900 | <0,001 |
|  | MTR | 5,556 | 1,238 | 222,568 | 3,117 | 7,996 | <0,001 |
| Month 12 | STR | 14,568 | 1,454 | 259,464 | 11,704 | 17,431 | <0,001 |
|  | MTR | 5,576 | 1,233 | 219,893 | 3,145 | 8,007 | <0,001 |
| Month 18 | STR | 14,947 | 1,479 | 252,323 | 12,035 | 17,860 | <0,001 |
|  | MTR | 5,929 | 1,248 | 223,750 | 3,470 | 8,388 | <0,001 |
| Month 24 | STR | 13,508 | 1,515 | 226,953 | 10,522 | 16,494 | <0,001 |
|  | MTR | 4,725 | 1,264 | 219,777 | 2,235 | 7,215 | <0,001 |

**BDI: Graph**


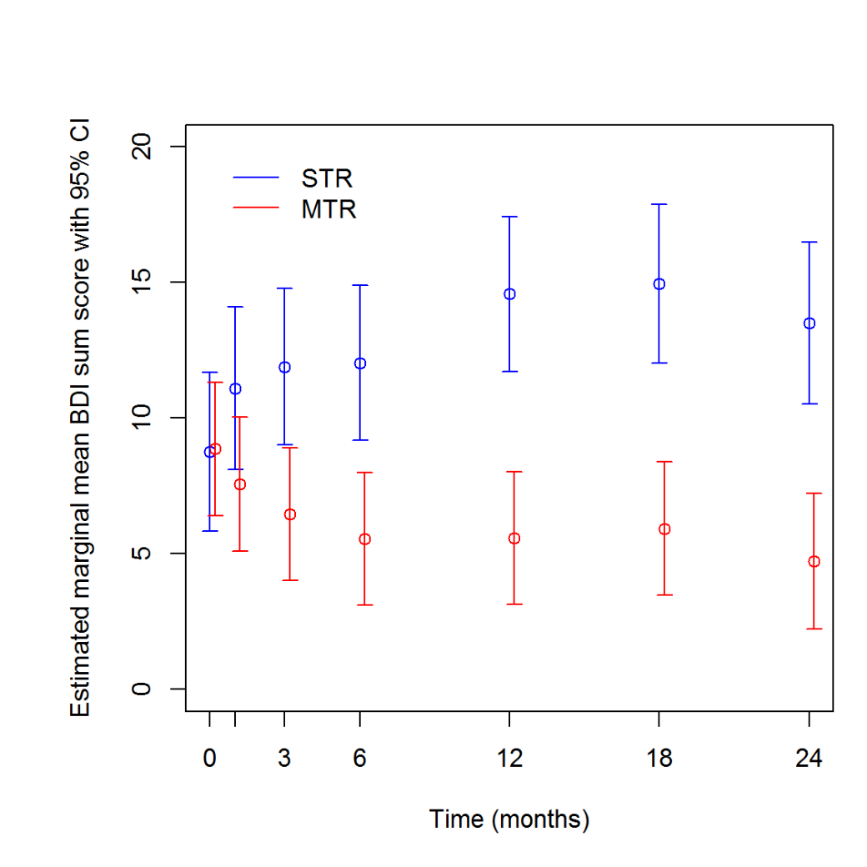


## Quality of life: EuroQol Utility (EuroQol 5D-3L, range 0-1, higher score = better outcome)

Model with random intercept and time categorical.
-2Restricted Log Likelihood: -1112.032
AIC: -1108.032
94.4% of data were available (1243/1316).

| **EuroQol Utility: Estimates of Fixed Effects** | | | | | | | | |
| --- | --- | --- | --- | --- | --- | --- | --- | --- |
| Parameter | Estimate | Std. Error | df | t | Sig. | 95% Confidence Interval | |  |
|  |  |  |  |  |  | Lower Bound | Upper Bound |  |
| Intercept | ,906657 | ,017763 | 365,376 | 51,042 | ,000 | ,871727 | ,941588 |  |
| Baseline | -,071515 | ,015644 | 991,298 | -4,571 | ,000 | -,102214 | -,040816 |  |
| Month 1 | -,052441 | ,015999 | 992,850 | -3,278 | ,001 | -,083837 | -,021045 |  |
| Month 3 | -,057367 | ,015719 | 991,683 | -3,650 | ,000 | -,088214 | -,026521 |  |
| Month 6 | -,042231 | ,015957 | 992,718 | -2,646 | ,008 | -,073546 | -,010917 |  |
| Month 12 | -,063172 | ,015798 | 992,116 | -3,999 | ,000 | -,094173 | -,032172 |  |
| Month 18 | -,016465 | ,015917 | 992,522 | -1,034 | ,301 | -,047699 | ,014769 |  |
| Month 24 | 0 | 0 | . | . | . | . | . |  |
| STR group | -,154647 | ,030667 | 409,520 | -5,043 | ,000 | -,214931 | -,094363 |  |
| MTR group | 0 | 0 | . | . | . | . | . |  |
| Baseline * STR group | ,117625 | ,028607 | 990,868 | 4,112 | ,000 | ,061487 | ,173762 |  |
| Baseline * MTR group | 0 | 0 | . | . | . | . | . |  |
| Month 1 * STR group | ,086437 | ,030270 | 994,822 | 2,856 | ,004 | ,027037 | ,145836 |  |
| Month 1 * MTR group | 0 | 0 | . | . | . | . | . |  |
| Month 3 * STR group | ,074042 | ,028875 | 991,588 | 2,564 | ,010 | ,017379 | ,130706 |  |
| Month 3 * MTR group | 0 | 0 | . | . | . | . | . |  |
| Month 6 * STR group | ,028124 | ,029128 | 992,302 | ,966 | ,335 | -,029037 | ,085284 |  |
| Month 6 * MTR group | 0 | 0 | . | . | . | . | . |  |
| Month 12 * STR group | ,011606 | ,029036 | 991,976 | ,400 | ,689 | -,045373 | ,068586 |  |
| Month 12 * MTR group | 0 | 0 | . | . | . | . | . |  |
| Month 18 * STR group | -,009027 | ,029225 | 992,476 | -,309 | ,757 | -,066378 | ,048324 |  |
| Month 18 * MTR group | 0 | 0 | . | . | . | . | . |  |
| Month 24 * STR group | 0 | 0 | . | . | . | . | . |  |
| Month 24 * MTR group | 0 | 0 | . | . | . | . | . |  |
| Women | -,021184 | ,037888 | 173,995 | -,559 | ,577 | -,095963 | ,053595 |  |
| Men | 0 | 0 | . | . | . | . | . |  |
| Non-Caucasian | ,026782 | ,045044 | 174,611 | ,595 | ,553 | -,062120 | ,115683 |  |
| Caucasian | 0 | 0 | . | . | . | . | . |  |
| Heterosexual | -,011098 | ,030448 | 174,172 | -,365 | ,716 | -,071192 | ,048996 |  |
| Homosexual | 0 | 0 | . | . | . | . | . |  |

Intercept:
At 2y, MTR, Caucasian, homosexual men have a utility of 0.9067 (p<0.001)

Time:

Within the MTR-group:
at baseline, 0.072 less than at 2y (p<0.001)
at T1, 0.052 less (p=0.001)
at T2, 0.057 less (p<0.001)
at T3 0.042 less (p=0.008)
at 1y, 0.063 less than at 2y (p<0.001)

*A second model (in which 2y+STR-group are the reference groups) shows significant different scores within the STR-group as compared to 2y:
at 1y 0.052 less than at 2y (0.035)*

*A third model (in which baseline+MTR are the reference groups) shows significantly different scores within the MTR-group as compared to baseline:
at T5 0.055 more than at baseline (p=0.001)
at T6 0.072 more than at baseline (p<0.001)*

*A fourth model (in which baseline+STR are the reference groups) shows significant differences within the STR-group as compared to baseline:
at T3 0.060 less than at baseline (0.014)
at T4 0.098 less than at baseline (<0.001)
at T5 0.072 less than at baseline (p=0.004)*

Stable:
At 2y, the STR-group’s utility is significantly lower (-0.155) as compared to the utility in the MTR-group (p<0.001).

Interaction:
The estimated mean utility difference between baseline and 2y between the groups is 0.118 (p<0.001)
- Utility in the STR-group decreased with 0.046, utility in the MTR-group increased with 0.072 over time
- The STR-group had a 0.037 lower utility at baseline and 0.155 lower at 2y.

Differences according to gender, ethnicity and sexual orientation are not significant.

| **EuroQoL Utility: Estimates** | | | | | | |  |
| --- | --- | --- | --- | --- | --- | --- | --- |
| Time | Group | Mean | Std. Error | Df | 95% Confidence Interval | |  |
|  |  |  |  |  | Lower Bound | Upper Bound | Sig |
| Baseline | STR | ,795 | ,029 | 334,486 | ,738 | ,852 | ,229 |
|  | MTR | ,832 | ,024 | 260,287 | ,785 | ,880 | ,229 |
| Month 1 | STR | ,783 | ,030 | 395,523 | ,723 | ,843 | ,035 |
|  | MTR | ,851 | ,024 | 270,043 | ,804 | ,899 | ,035 |
| Month 3 | STR | ,766 | ,029 | 342,412 | ,708 | ,823 | ,010 |
|  | MTR | ,847 | ,024 | 260,947 | ,799 | ,894 | ,010 |
| Month 6 | STR | ,735 | ,029 | 351,086 | ,677 | ,793 | <0,001 |
|  | MTR | ,862 | ,024 | 266,722 | ,814 | ,909 | <0,001 |
| Month 12 | STR | ,698 | ,029 | 349,021 | ,640 | ,755 | <0,001 |
|  | MTR | ,841 | ,024 | 264,166 | ,793 | ,888 | <0,001 |
| Month 18 | STR | ,724 | ,030 | 354,896 | ,666 | ,782 | <0,001 |
|  | MTR | ,887 | ,024 | 267,317 | ,840 | ,935 | <0,001 |
| Month 24 | STR | ,749 | ,029 | 334,486 | ,692 | ,806 | <0,001 |
|  | MTR | ,904 | ,024 | 256,504 | ,857 | ,951 | <0,001 |

**EuroQoL Utility: Graph**


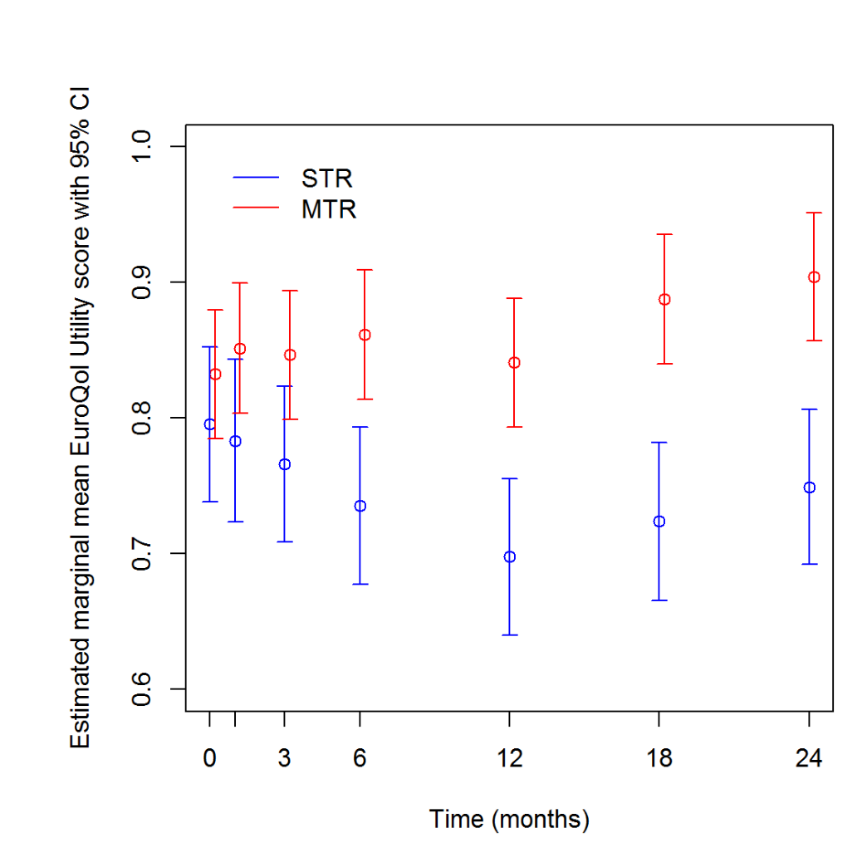


## Quality of Life: Visual Analogue Scale (EuroQol VAS, range 0-100, higher score = better outcome)

Model with random intercept, random slope, unstructured covariance structure and time categorical.
-2Restricted Log Likelihood: 8787.874
AIC: 8795.874
94.4% of data were available (1242/1316).

| **EuroQoL VAS: Estimates of Fixed Effects** | | | | | | | |
| --- | --- | --- | --- | --- | --- | --- | --- |
| Parameter | Estimate | Std. Error | df | t | Sig. | 95% Confidence Interval | |
|  |  |  |  |  |  | Lower Bound | Upper Bound |
| Intercept | 85,565029 | 1,332199 | 241,364 | 64,228 | ,000 | 82,940808 | 88,189249 |
| Baseline | -7,404699 | 1,224820 | 343,973 | -6,046 | ,000 | -9,813780 | -4,995619 |
| Month 1 | -5,619142 | 1,229888 | 399,964 | -4,569 | ,000 | -8,036994 | -3,201290 |
| Month 3 | -4,395906 | 1,185175 | 478,356 | -3,709 | ,000 | -6,724698 | -2,067114 |
| Month 6 | -,998083 | 1,162616 | 677,453 | -,858 | ,391 | -3,280847 | 1,284681 |
| Month 12 | -1,174468 | 1,098654 | 980,609 | -1,069 | ,285 | -3,330452 | ,981516 |
| Month 18 | -2,225789 | 1,063421 | 911,063 | -2,093 | ,037 | -4,312829 | -,138749 |
| Month 24 | 0 | 0 | . | . | . | . | . |
| STR group | -9,552828 | 2,299706 | 235,774 | -4,154 | ,000 | -14,083424 | -5,022231 |
| MTR group | 0 | 0 | . | . | . | . | . |
| Baseline * STR group | 8,809794 | 2,239029 | 338,960 | 3,935 | ,000 | 4,405653 | 13,213935 |
| Baseline * MTR group | 0 | 0 | . | . | . | . | . |
| Month 1 * STR group | 6,536394 | 2,312928 | 426,446 | 2,826 | ,005 | 1,990237 | 11,082552 |
| Month 1 * MTR group | 0 | 0 | . | . | . | . | . |
| Month 3 * STR group | 3,083205 | 2,177296 | 478,045 | 1,416 | ,157 | -1,195049 | 7,361459 |
| Month 3 * MTR group | 0 | 0 | . | . | . | . | . |
| Month 6 * STR group | 1,649700 | 2,117207 | 664,406 | ,779 | ,436 | -2,507521 | 5,806922 |
| Month 6 * MTR group | 0 | 0 | . | . | . | . | . |
| Month 12 * STR group | ,287635 | 2,012773 | 979,769 | ,143 | ,886 | -3,662207 | 4,237476 |
| Month 12 * MTR group | 0 | 0 | . | . | . | . | . |
| Month 18 * STR group | -,484446 | 1,947236 | 910,160 | -,249 | ,804 | -4,306040 | 3,337148 |
| Month 18 * MTR group | 0 | 0 | . | . | . | . | . |
| Month 24 * STR group | 0 | 0 | . | . | . | . | . |
| Month 24 * MTR group | 0 | 0 | . | . | . | . | . |
| Women | ,538554 | 2,843993 | 174,165 | ,189 | ,850 | -5,074573 | 6,151681 |
| Men | 0 | 0 | . | . | . | . | . |
| Non-Caucasian | 4,804843 | 3,378078 | 174,170 | 1,422 | ,157 | -1,862396 | 11,472081 |
| Caucasian | 0 | 0 | . | . | . | . | . |
| Heterosexual | -4,851004 | 2,285023 | 174,143 | -2,123 | ,035 | -9,360908 | -,341100 |
| Homosexual | 0 | 0 | . | . | . | . | . |

Intercept:
At 2y, MTR, Caucasian, homosexual men have a score of 85.57 (p<0.001)

Time:

Within the MTR-group, there are differences as compared to 2y
at baseline, 7.40 less (p<0.001)
at T1 5.62 less (p<0.001)
at T2 4.39 less (p<0.001)
at T5 2.23 less (p=0.037)

*A second model (in which 2y+STR-group are the reference groups) shows no significant different scores within the STR-group as compared to 2y.*

*A third model (in which baseline+MTR are the reference groups) shows a significantly different score within the MTR-group as compared to baseline
at T2 3.01 more (p=0.004)
at T3 6.41 more (p<0.001)
at T4 6.23 more (p<0.001)
at T5 5.18 more (p<0.001)
at T6 7.40 more (p<0.001)*

*A fourth model (in which baseline+STR are the reference groups) shows only a significant difference within the STR-group at T5 (4.12 more, p=0.021) as compared to baseline.*

Stable: At 2y, the score in the STR-group is 9.55 points lower (p<0.001).

Interaction:
The estimated mean difference between baseline and 2y between the groups is 8.81. This difference is significant (p<0.001)
- VAS-score in the STR-group decreased by 1.405 , the score in the MTR-group increased by 7.404 over time
- The STR-group had a 0.744 lower score at baseline and 9.553 lower at 2y.

Differences according to gender and ethnicity are not significant. We do see that at 2y, heterosexual participants’ score is 4.85 lower than homosexual participants (p=0.035).

| **EuroQoL VAS: Estimates** | | | | | | |  |
| --- | --- | --- | --- | --- | --- | --- | --- |
| Time | Group | Mean | Std. Error | Df | 95% Confidence Interval | |  |
|  |  |  |  |  | Lower Bound | Upper Bound | Sig |
| Baseline | STR | 77,663 | 2,152 | 276,573 | 73,427 | 81,900 | ,744 |
|  | MTR | 78,407 | 1,792 | 242,328 | 74,877 | 81,936 | ,744 |
| Month 1 | STR | 77,176 | 2,237 | 325,373 | 72,776 | 81,576 | ,202 |
|  | MTR | 80,192 | 1,802 | 249,500 | 76,644 | 83,740 | ,202 |
| Month 3 | STR | 74,946 | 2,142 | 292,421 | 70,731 | 79,161 | ,004 |
|  | MTR | 81,415 | 1,780 | 241,500 | 77,909 | 84,922 | ,004 |
| Month 6 | STR | 76,910 | 2,129 | 300,436 | 72,720 | 81,100 | <0.001 |
|  | MTR | 84,813 | 1,781 | 244,986 | 81,306 | 88,321 | <0.001 |
| Month 12 | STR | 75,372 | 2,127 | 302,729 | 71,187 | 79,557 | <0.001 |
|  | MTR | 84,637 | 1,776 | 242,962 | 81,139 | 88,134 | <0.001 |
| Month 18 | STR | 73,548 | 2,150 | 286,972 | 69,316 | 77,780 | <0.001 |
|  | MTR | 83,585 | 1,787 | 241,961 | 80,065 | 87,106 | <0.001 |
| Month 24 | STR | 76,258 | 2,176 | 242,857 | 71,972 | 80,545 | <0.001 |
|  | MTR | 85,811 | 1,797 | 229,951 | 82,271 | 89,352 | <0.001 |

**EuroQoL VAS: Graph**


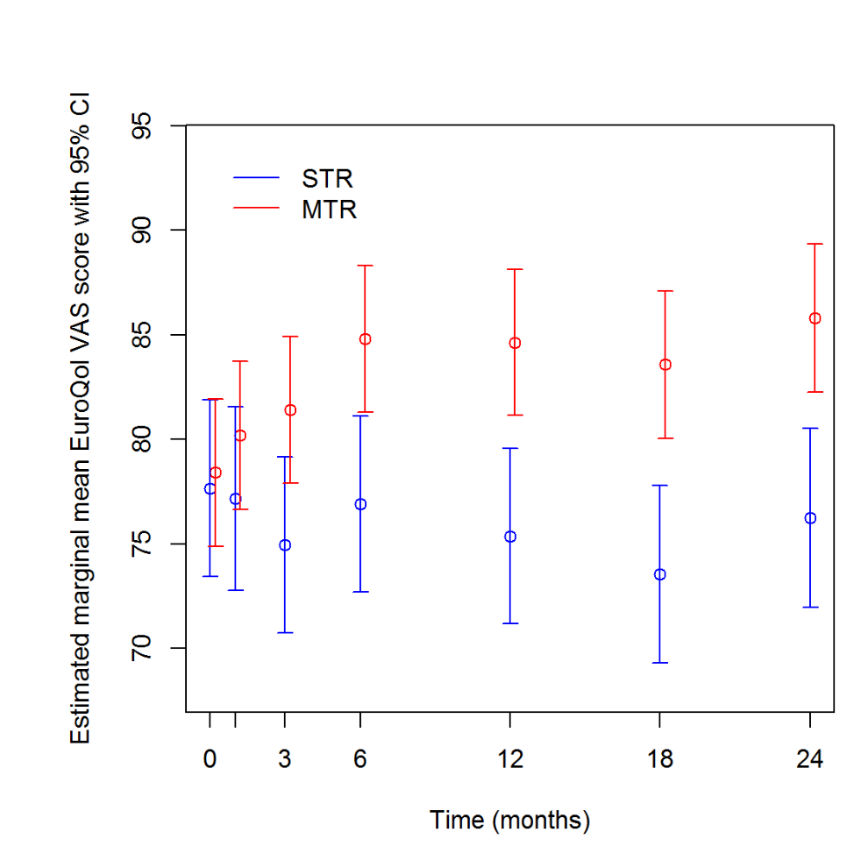


## Quality of Life: Physical health (Physical health score of MOS-HIV, standardized scale (T-score) with a mean of 50 and a standard deviation of 10 in the sample in which the summary scores were developed, higher score = better outcomes)

Model with random intercept, random slope, unstructured covariance structure and time continuous.
-2Restricted Log Likelihood: 7633.626
AIC: 7641.626
94.4% of data were available (1243/1316).

| **MOS-HIV PHS: Estimates of Fixed Effects** | | | | | | | |
| --- | --- | --- | --- | --- | --- | --- | --- |
| Parameter | Estimate | Std. Error | df | t | Sig. | 95% Confidence Interval | |
|  |  |  |  |  |  | Lower Bound | Upper Bound |
| Intercept | 54,094017 | ,880407 | 186,522 | 61,442 | ,000 | 52,357181 | 55,830853 |
| Time continuous | ,101585 | ,027059 | 173,344 | 3,754 | ,000 | ,048178 | ,154991 |
| STR group | -9,411948 | 1,504976 | 176,040 | -6,254 | ,000 | -12,382065 | -6,441830 |
| MTR group | 0^b^ | 0 | . | . | . | . | . |
| STR * Time continuous | -,329118 | ,049894 | 175,483 | -6,596 | ,000 | -,427588 | -,230648 |
| MTR * Time continuous | 0^b^ | 0 | . | . | . | . | . |
| Women | -,328789 | 2,010679 | 173,367 | -,164 | ,870 | -4,297351 | 3,639773 |
| Men | 0^b^ | 0 | . | . | . | . | . |
| Non-Caucasian | 3,727923 | 2,389446 | 173,689 | 1,560 | ,121 | -,988165 | 8,444011 |
| Caucasian | 0^b^ | 0 | . | . | . | . | . |
| Heterosexual | -2,696605 | 1,615967 | 173,612 | -1,669 | ,097 | -5,886074 | ,492865 |
| Homosexual | 0^b^ | 0 | . | . | . | . | . |

Intercept: At 2y MTR-, Caucasian, homosexual men have a score of 54.09 (p<0.001).

Time: Per month, PHS increases by 0.10 in the MTR-group (p<0.001).
*In the STR-group, PHS decreases by 0.23 (p<0.001).*

Stable: At 2y, the STR-group has a 9.41 lower PHS than the MTR-group (p<0.001)

Interaction: The estimated mean increase in PHS per month is 0.32 lower among STR-group as compared to MTR-group (the 95% CI goes from 0.43 to 0.23 lower), p<0.001.

No differences according to gender, ethnicity and sexual orientation.

| **Estimates** | | | | | | |  |
| --- | --- | --- | --- | --- | --- | --- | --- |
| Time | Group | Mean | Std. Error | df | 95% Confidence Interval | |  |
|  |  |  |  |  | Lower Bound | Upper Bound | Sig |
| Baseline | STR | 50,494 | 1,349 | 181,755 | 47,832 | 53,156 | ,269 |
|  | MTR | 52,007 | 1,176 | 182,591 | 49,687 | 54,328 | ,269 |
| Month 1 | STR | 50,267 | 1,339 | 180,194 | 47,625 | 52,908 | ,174 |
|  | MTR | 52,109 | 1,171 | 180,756 | 49,798 | 54,420 | ,174 |
| Month 3 | STR | 49,812 | 1,321 | 177,456 | 47,205 | 52,418 | ,061 |
|  | MTR | 52,312 | 1,163 | 177,706 | 50,017 | 54,607 | ,061 |
| Month 6 | STR | 49,129 | 1,304 | 174,639 | 46,555 | 51,703 | ,008 |
|  | MTR | 52,617 | 1,155 | 174,799 | 50,337 | 54,897 | ,008 |
| Month 12 | STR | 47,764 | 1,307 | 174,898 | 45,184 | 50,343 | <0,001 |
|  | MTR | 53,226 | 1,157 | 175,399 | 50,943 | 55,509 | <0,001 |
| Month 18 | STR | 46,399 | 1,357 | 182,541 | 43,721 | 49,076 | <0,001 |
|  | MTR | 53,836 | 1,181 | 184,367 | 51,506 | 56,166 | <0,001 |
| Month 24 | STR | 45,033 | 1,450 | 193,596 | 42,174 | 47,893 | <0,001 |
|  | MTR | 54,445 | 1,226 | 200,024 | 52,027 | 56,864 | <0,001 |

**MOS-HIV PHS: Graph**


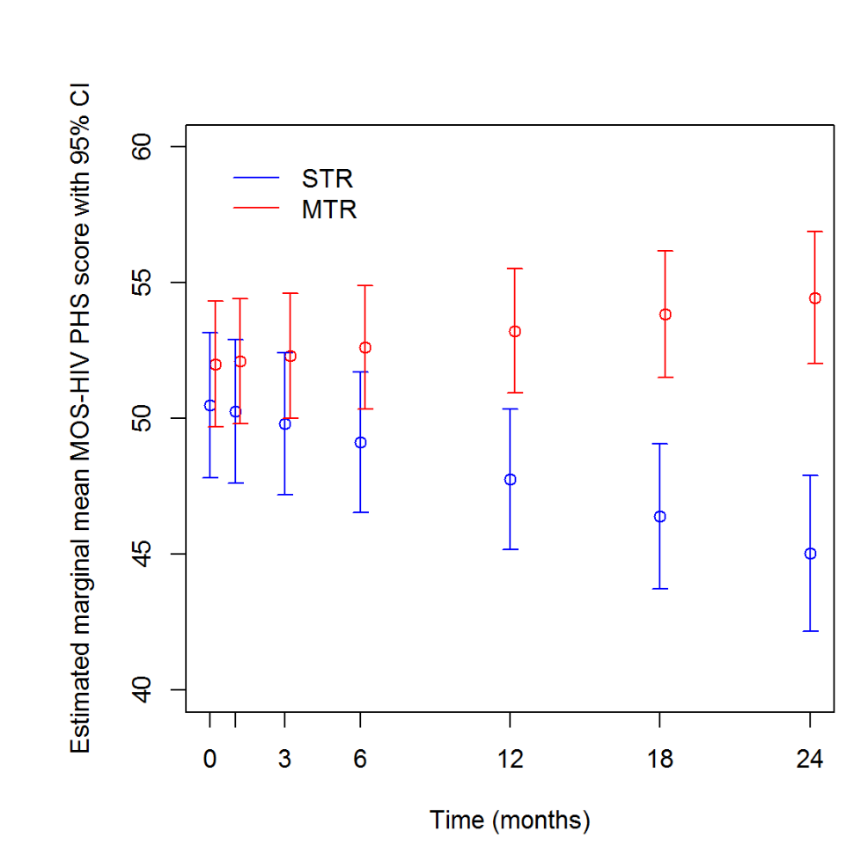


## Quality of Life: Mental health (Mental health score of MOS-HIV, standardized scale (T-score) with a mean of 50 and a standard deviation of 10 in the sample in which the summary scores were developed, higher score = better outcomes)

Model with random intercept, random slope, unstructured covariance structure and time categorical.
-2Restricted Log Likelihood: 7886.062
AIC: 7894.062
94.4% of data were available (1243/1316).

| **MOS-HIV MHS: Estimates of Fixed Effects** | | | | | | | |
| --- | --- | --- | --- | --- | --- | --- | --- |
| Parameter | Estimate | Std. Error | df | t | Sig. | 95% Confidence Interval | |
|  |  |  |  |  |  | Lower Bound | Upper Bound |
| Intercept | 54,447539 | ,989374 | 232,549 | 55,032 | ,000 | 52,498258 | 56,396820 |
| Baseline | -4,701326 | ,878186 | 296,321 | -5,353 | ,000 | -6,429598 | -2,973054 |
| Month 1 | -2,490399 | ,879068 | 344,004 | -2,833 | ,005 | -4,219424 | -,761374 |
| Month 3 | -1,587207 | ,838316 | 401,352 | -1,893 | ,059 | -3,235246 | ,060833 |
| Month 6 | -,800850 | ,808669 | 571,107 | -,990 | ,322 | -2,389179 | ,787478 |
| Month 12 | -1,421032 | ,737573 | 939,055 | -1,927 | ,054 | -2,868514 | ,026451 |
| Month 18 | -1,370361 | ,702392 | 929,963 | -1,951 | ,051 | -2,748819 | ,008097 |
| Month 24 | 0 | 0 | . | . | . | . | . |
| STR group | -7,171224 | 1,715754 | 221,843 | -4,180 | ,000 | -10,552487 | -3,789961 |
| MTR group | 0 | 0 | . | . | . | . | . |
| Baseline * STR group | 9,160507 | 1,616279 | 297,723 | 5,668 | ,000 | 5,979728 | 12,341286 |
| Baseline * MTR group | 0 | 0 | . | . | . | . | . |
| Month 1 * STR group | 3,450603 | 1,647995 | 365,656 | 2,094 | ,037 | ,209866 | 6,691341 |
| Month 1 * MTR group | 0 | 0 | . | . | . | . | . |
| Month 3 * STR group | 1,473933 | 1,546732 | 406,027 | ,953 | ,341 | -1,566669 | 4,514534 |
| Month 3 * MTR group | 0 | 0 | . | . | . | . | . |
| Month 6 * STR group | ,942701 | 1,478953 | 563,581 | ,637 | ,524 | -1,962232 | 3,847633 |
| Month 6 * MTR group | 0 | 0 | . | . | . | . | . |
| Month 12 * STR group | ,281624 | 1,355615 | 939,186 | ,208 | ,835 | -2,378760 | 2,942008 |
| Month 12 * MTR group | 0 | 0 | . | . | . | . | . |
| Month 18 * STR group | ,767693 | 1,283632 | 929,449 | ,598 | ,550 | -1,751459 | 3,286846 |
| Month 18 * MTR group | 0 | 0 | . | . | . | . | . |
| Month 24 * STR group | 0 | 0 | . | . | . | . | . |
| Month 24 * MTR group | 0 | 0 | . | . | . | . | . |
| Women | ,744716 | 2,037735 | 174,158 | ,365 | ,715 | -3,277119 | 4,766551 |
| Men | 0 | 0 | . | . | . | . | . |
| Non-Caucasian | 1,603383 | 2,422122 | 174,638 | ,662 | ,509 | -3,177015 | 6,383781 |
| Caucasian | 0 | 0 | . | . | . | . | . |
| Heterosexual | -2,509657 | 1,638254 | 174,616 | -1,532 | ,127 | -5,742985 | ,723670 |
| Homosexual | 0 | 0 | . | . | . | . | . |

Intercept:
At 2y, MTR, Caucasian, homosexual men have a score of 54.45 (p<0.001)

Time:

Within the MTR-group, there are differences as compared to 2y
at baseline, 4.70 lower (p<0.001)
at T1 2.49 lower (p=0.005)

*A second model (in which 2y+STR-group are the reference groups) shows a significant different score within the STR-group as compared to 2y at baseline (4.46 higher, p=0.001).*

*A third model (in which baseline+MTR are the reference groups) shows a significantly different score within the MTR-group as compared to baseline
at T1 2.21 more (p<0.001)
at T2 3.11 more (p<0.001)
at T3 3.90 more (p<0.001)
at T4 3.28 more (p<0.001)
at T5 3.33 more (p<0.001)
at T6 4.70 more (p<0.001)*

*A fourth model (in which baseline+STR are the reference groups) shows only a significant difference within the STR-group as compared to baseline
at T1 3.50 lower (p<0.001)
at T2 4.57 lower (p<0.001)
at T3 4.32 lower (p<0.001)
at T4 5.60 lower (p<0.001)
at T5 5.06 lower (p<0.001)
at T6 4.46 lower (p<0.001)*

Stable: At 2y, the score in the STR-group is 7.17 points lower (p<0.001).

Interaction:
The estimated mean difference between baseline and 2y between the groups is 9.16. This difference is significant (p<0.001)
- MHS-score in the STR-group decreased by 4.459 , the score in the MTR-group increased by 4.702 over time
- The STR-group had a 1.99 higher score at baseline and 7.171 lower at 2y.

Differences according to gender, ethnicity and sexual orientation are not significant.

| **MOS-HIV MHS: Estimates** | | | | | | |  |
| --- | --- | --- | --- | --- | --- | --- | --- |
| Time | Group | Mean | Std. Error | df | 95% Confidence Interval | |  |
|  |  |  |  |  | Lower Bound | Upper Bound | Sig |
| Baseline | STR | 51,655 | 1,500 | 261,633 | 48,701 | 54,608 | ,204 |
|  | MTR | 49,665 | 1,256 | 225,423 | 47,190 | 52,141 | ,204 |
| Month 1 | STR | 48,156 | 1,547 | 298,144 | 45,112 | 51,200 | ,022 |
|  | MTR | 51,876 | 1,266 | 234,350 | 49,382 | 54,371 | ,022 |
| Month 3 | STR | 47,082 | 1,492 | 272,090 | 44,145 | 50,020 | <0,001 |
|  | MTR | 52,780 | 1,252 | 226,902 | 50,312 | 55,247 | <0,001 |
| Month 6 | STR | 47,337 | 1,490 | 279,674 | 44,405 | 50,269 | <0,001 |
|  | MTR | 53,566 | 1,257 | 232,414 | 51,089 | 56,042 | <0,001 |
| Month 12 | STR | 46,056 | 1,504 | 281,778 | 43,096 | 49,016 | <0,001 |
|  | MTR | 52,946 | 1,261 | 232,975 | 50,462 | 55,429 | <0,001 |
| Month 18 | STR | 46,593 | 1,551 | 272,334 | 43,540 | 49,645 | <0,001 |
|  | MTR | 52,996 | 1,287 | 240,689 | 50,461 | 55,532 | <0,001 |
| Month 24 | STR | 47,196 | 1,610 | 238,431 | 44,023 | 50,368 | <0,001 |
|  | MTR | 54,367 | 1,314 | 236,101 | 51,777 | 56,956 | <0,001 |

**MOS-HIV MHS: Graph**


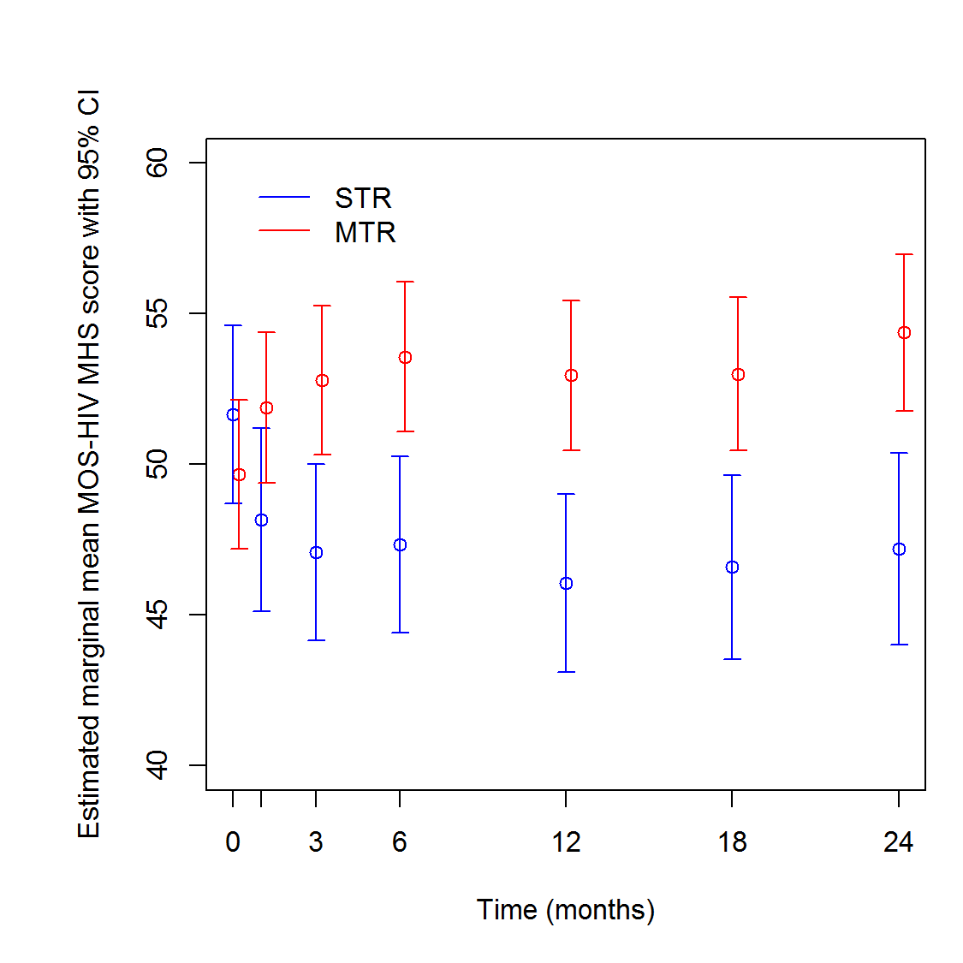


## Adherence: score (Case Adherence Index, range 0-16, higher score = better outcome)

Model with random intercept, random slope, unstructured covariance structure and time continuous.
-2Restricted Log Likelihood: 4314.865
AIC: 4322.865
94.1% of data were available (1239/1316).

| **CASE Adherence Index: Estimates of Fixed Effects** | | | | | | | | |
| --- | --- | --- | --- | --- | --- | --- | --- | --- |
| Parameter | Estimate | Std. Error | df | | T | Sig. | 95% Confidence Interval | |
|  |  |  |  |  |  |  | Lower Bound | Upper Bound |
| Intercept | 15,113045 | ,184845 | 190,061 | | 81,761 | ,000 | 14,748434 | 15,477656 |
| Time continuous | ,016479 | ,007088 | 177,610 | | 2,325 | ,021 | ,002492 | ,030466 |
| STR group | -1,532901 | ,317844 | 177,596 | | -4,823 | ,000 | -2,160139 | -,905663 |
| MTR group | 0 | 0 | . | | . | . | . | . |
| STR * Time continuous | -,034804 | ,013062 | 179,327 | | -2,665 | ,008 | -,060579 | -,009030 |
| MTR * Time continuous | 0 | 0 | . | . | | . | . | . |
| Women | ,338955 | ,405771 | 173,880 | | ,835 | ,405 | -,461915 | 1,139826 |
| Men | 0 | 0 | . | | . | . | . | . |
| Non-Caucasian | -,759750 | ,482636 | 174,776 | | -1,574 | ,117 | -1,712296 | ,192796 |
| Caucasian | 0 | 0 | . | | . | . | . | . |
| Heterosexual | -,223818 | ,326047 | 173,978 | | -,686 | ,493 | -,867335 | ,419700 |
| Homosexual | 0 | 0 | . | | . | . | . | . |

Intercept: At 2y MTR-, Caucasian, homosexual men have a score of 15.11 (p<0.001).

Time: Per month, CASE SUM score increases by 0.016 in the MTR-group (p=0.021).
*In the STR-group, evolution is not significant (decrease of 0.018, p=0.097).*

Stable: At 2y, the STR-group has a 1.53 lower score than the MTR-group (p<0.001)

Interaction: The estimated mean increase in PHS per month is 0.035 lower among STR-group as compared to MTR-group (the 95% CI goes from 0.061 to 0.009 lower), p=0.008.

No differences according to gender, ethnicity and sexual orientation.

| **CASE Adherence Index: Estimates** | | | | | | |  |
| --- | --- | --- | --- | --- | --- | --- | --- |
| Time | Group | Mean | Std. Error | df | 95% Confidence Interval | |  |
|  |  |  |  |  | Lower Bound | Upper Bound | Sig |
| Baseline | STR | 13,698 | ,285 | 193,764 | 13,135 | 14,261 | ,018 |
|  | MTR | 14,395 | ,244 | 195,527 | 13,915 | 14,876 | ,018 |
| Month 1 | STR | 13,679 | ,281 | 191,061 | 13,124 | 14,234 | ,012 |
|  | MTR | 14,412 | ,242 | 191,985 | 13,935 | 14,888 | ,012 |
| Month 3 | STR | 13,643 | ,274 | 185,827 | 13,102 | 14,184 | ,004 |
|  | MTR | 14,445 | ,238 | 185,742 | 13,975 | 14,915 | ,004 |
| Month 6 | STR | 13,588 | ,267 | 179,305 | 13,062 | 14,114 | ,001 |
|  | MTR | 14,494 | ,235 | 178,896 | 14,031 | 14,957 | ,001 |
| Month 12 | STR | 13,478 | ,263 | 175,485 | 12,958 | 13,997 | <0,001 |
|  | MTR | 14,593 | ,233 | 175,880 | 14,132 | 15,054 | <0,001 |
| Month 18 | STR | 13,368 | ,276 | 184,895 | 12,823 | 13,913 | <0,001 |
|  | MTR | 14,692 | ,240 | 187,289 | 14,219 | 15,165 | <0,001 |
| Month 24 | STR | 13,258 | ,303 | 199,067 | 12,660 | 13,855 | <0,001 |
|  | MTR | 14,791 | ,253 | 209,335 | 14,292 | 15,289 | <0,001 |

**CASE Adherence Index: Graph**


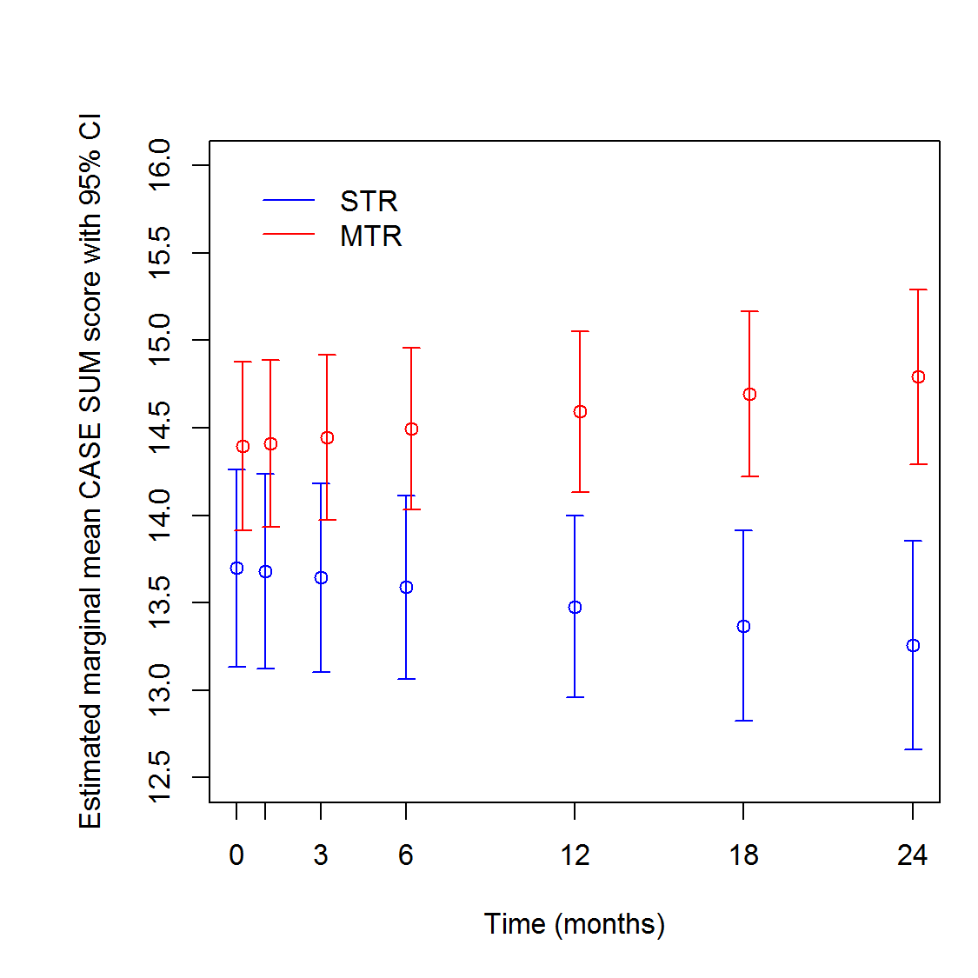


## Adherence: VAS (Visual Analogue Scale, range 0-100, higher score = better outcome)

Model with random intercept, random slope, unstructured covariance structure and time categorical.
-2Restricted Log Likelihood: 7391.508
AIC: 7399.508
94.3% of data were available (1241/1316).

| **Adherence VAS: Estimates of Fixed Effects** | | | | | | | |
| --- | --- | --- | --- | --- | --- | --- | --- |
| Parameter | Estimate | Std. Error | df | t | Sig. | 95% Confidence Interval | |
|  |  |  |  |  |  | Lower Bound | Upper Bound |
| Intercept | 98,423280 | ,483074 | 184,032 | 203,744 | ,000 | 97,470206 | 99,376355 |
| Time continuous | ,035502 | ,024835 | 176,012 | 1,430 | ,155 | -,013511 | ,084515 |
| STR group | -3,157514 | ,832289 | 173,132 | -3,794 | ,000 | -4,800253 | -1,514775 |
| MTR group | 0 | 0 | . | . | . | . | . |
| STR * Time continuous | -,053656 | ,045782 | 177,865 | -1,172 | ,243 | -,144002 | ,036690 |
| MTR * Time continuous | 0 | 0 | . | . | . | . | . |
| Women | ,213915 | 1,061401 | 171,182 | ,202 | ,841 | -1,881205 | 2,309035 |
| Men | 0 | 0 | . | . | . | . | . |
| Non-Caucasian | -4,590011 | 1,261024 | 171,240 | -3,640 | ,000 | -7,079164 | -2,100859 |
| Caucasian | 0 | 0 | . | . | . | . | . |
| Heterosexual | -,920557 | ,850866 | 169,748 | -1,082 | ,281 | -2,600198 | ,759085 |
| Homosexual | 0 | 0 | . | . | . | . | . |

Intercept: At 2y MTR-, Caucasian, homosexual men have a score of 98.42 (p<0.001).

Time: Per month, CASEVAS score increases by 0.035 in the MTR-group, however, this is not significant, p=0.155).
*In the STR-group, evolution is not significant (decrease of 0.018, p=0.637).*

Stable: At 2y, the STR-group has a 3.16 lower score than the MTR-group (p<0.001)

Interaction: The estimated mean increase in PHS per month is not different from the STR-group (0.054, p=0.243).

No differences according to gender and sexual orientation. Non-Caucasian participants have a 4.59 lower score than Caucasian participants.

| **Adherence VAS: Estimates** | | | | | | |  |
| --- | --- | --- | --- | --- | --- | --- | --- |
| Time | Group | Mean | Std. Error | df | 95% Confidence Interval | |  |
|  |  |  |  |  | Lower Bound | Upper Bound | Sig |
| Baseline | STR | 93,053 | ,861 | 203,561 | 91,356 | 94,750 | ,044 |
|  | MTR | 94,923 | ,695 | 224,492 | 93,553 | 96,293 | ,044 |
| Month 1 | STR | 93,035 | ,838 | 201,840 | 91,382 | 94,688 | ,033 |
|  | MTR | 94,958 | ,684 | 219,081 | 93,611 | 96,306 | ,033 |
| Month 3 | STR | 92,999 | ,796 | 197,216 | 91,428 | 94,569 | ,016 |
|  | MTR | 95,029 | ,663 | 207,911 | 93,723 | 96,336 | ,016 |
| Month 6 | STR | 92,944 | ,744 | 187,962 | 91,476 | 94,412 | ,005 |
|  | MTR | 95,136 | ,637 | 192,003 | 93,880 | 96,392 | ,005 |
| Month 12 | STR | 92,835 | ,687 | 171,670 | 91,478 | 94,192 | <0,001 |
|  | MTR | 95,349 | ,610 | 172,650 | 94,145 | 96,553 | <0,001 |
| Month 18 | STR | 92,726 | ,706 | 176,013 | 91,333 | 94,119 | <0,001 |
|  | MTR | 95,562 | ,619 | 178,052 | 94,341 | 96,783 | <0,001 |
| Month 24 | STR | 92,617 | ,794 | 194,917 | 91,052 | 94,183 | <0,001 |
|  | MTR | 95,775 | ,662 | 205,056 | 94,470 | 97,080 | <0,001 |

**Adherence VAS: Graph**


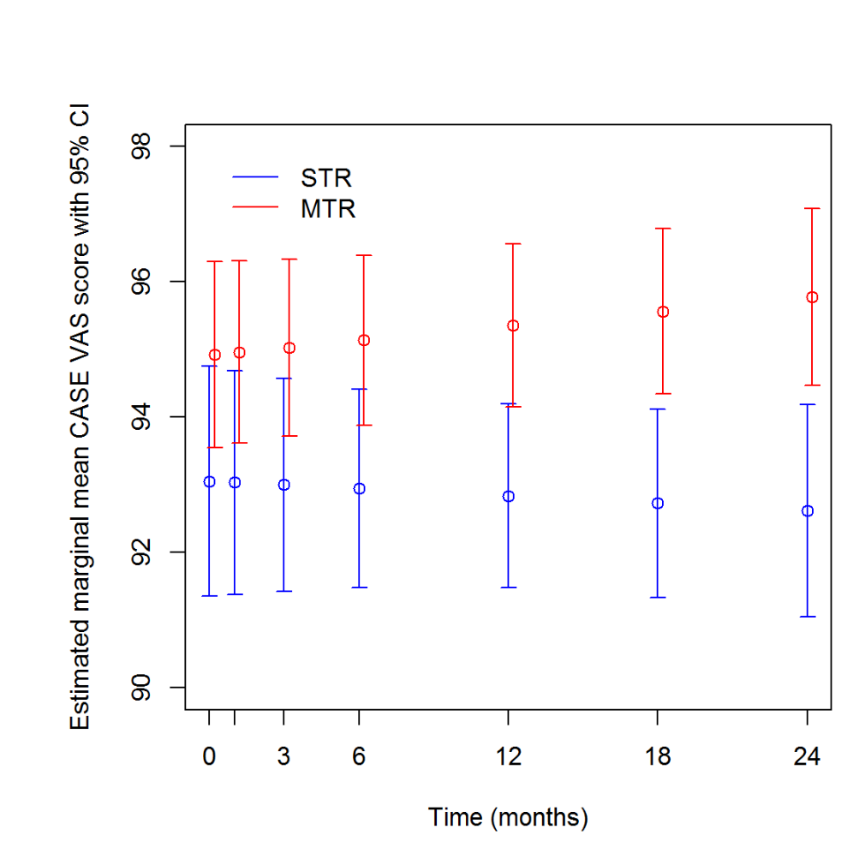


## Treatment satisfaction (HIV Treatment Satisfaction Questionnaire, 0-60, higher score = better outcome)

Model with random intercept, random slope, unstructured covariance structure and time categorical.
-2Restricted Log Likelihood: 4086.699
AIC: 4094.699

94.9% of data were available 714/752) (188*4 = 752)

| **Treatment Satisfaction: Estimates of Fixed Effects** | | | | | | | |
| --- | --- | --- | --- | --- | --- | --- | --- |
| Parameter | Estimate | Std. Error | df | T | Sig. | 95% Confidence Interval | |
|  |  |  |  |  |  | Lower Bound | Upper Bound |
| Intercept | 54,293673 | ,713986 | 208,171 | 76,043 | ,000 | 52,886103 | 55,701242 |
| Baseline | ,151972 | ,553642 | 326,739 | ,274 | ,784 | -,937182 | 1,241125 |
| Month 12 | ,108488 | ,564384 | 327,739 | ,192 | ,848 | -1,001784 | 1,218760 |
| Month 18 | -,148668 | ,581062 | 329,203 | -,256 | ,798 | -1,291732 | ,994396 |
| Month 24 | 0 | 0 | . | . | . | . | . |
| STR group | 3,137894 | 1,350935 | 241,464 | 2,323 | ,021 | ,476772 | 5,799017 |
| MTR group | 0 | 0 | . | . | . | . | . |
| Baseline * STR group | -3,496733 | 1,169928 | 327,671 | -2,989 | ,003 | -5,798250 | -1,195216 |
| Baseline * MTR group | 0 | 0 | . | . | . | . | . |
| Month 12 * STR group | -2,553884 | 1,193695 | 332,121 | -2,139 | ,033 | -4,902039 | -,205729 |
| Month 12 * MTR group | 0 | 0 | . | . | . | . | . |
| Month 18 * STR group | -2,516232 | 1,194444 | 328,912 | -2,107 | ,036 | -4,865946 | -,166518 |
| Month 18 * MTR group | 0 | 0 | . | . | . | . | . |
| Month 24 * STR group | 0 | 0 | . | . | . | . | . |
| Month 24 * MTR group | 0 | 0 | . | . | . | . | . |
| Women | ,128171 | 1,618611 | 123,057 | ,079 | ,937 | -3,075755 | 3,332097 |
| Men | 0 | 0 | . | . | . | . | . |
| Non-Caucasian | ,024943 | 2,209635 | 129,316 | ,011 | ,991 | -4,346773 | 4,396659 |
| Caucasian | 0 | 0 | . | . | . | . | . |
| Heterosexual | -1,036714 | 1,300589 | 122,110 | -,797 | ,427 | -3,611337 | 1,537909 |
| Homosexual | 0 | 0 | . | . | . | . | . |

Intercept:
At 2y, MTR, Caucasian, homosexual men have a score of 56.09 (p<0.001)

Time:

Within the MTR-group, there is a differences as compared to 2y at baseline, 2.11 lower (p=0.001).

*A second model (in which 2y+STR-group are the reference groups) shows no significant different scores within the STR-group as compared to 2y.*

*A third model (in which baseline+MTR are the reference groups) shows a significantly different score within the MTR-group as compared to baseline
at T4 1.70 more (p=0.001)
at T5 1.51 more (p=0.008)
at T6 2.11 more (p=0.001)*

*A fourth model (in which baseline+STR are the reference groups) shows only a significant difference within the STR-group as compared to baseline
at T4 2.03 lower (p=0.013)
at T5 2.67 lower (p=0.003)*

Stable: At 2y, the score in the STR-group is 3.93 points lower (p<0.001).

Interaction:
The estimated mean difference between baseline and 2y between the groups is 3.88. This difference is significant (p=0.001)
- TS score in the STR-group decreased by 1.77, the score in the MTR-group increased by 2.11 over time
- The STR-group had a 0.06 lower score at baseline and 3.93 lower at 2y.

Differences according to gender, ethnicity and sexual orientation are not significant.

| **Treatment Satisfaction: Estimates** | | | | | | |  |
| --- | --- | --- | --- | --- | --- | --- | --- |
| Time | Group | Mean | Std. Error | df | 95% Confidence Interval | |  |
|  |  |  |  |  | Lower Bound | Upper Bound | Sig |
| Baseline | STR | 53,650 | ,992 | 220,976 | 51,695 | 55,605 | ,957 |
|  | MTR | 53,706 | ,808 | 220,730 | 52,115 | 55,298 | ,957 |
| Month 12 | STR | 51,616 | ,947 | 279,045 | 49,752 | 53,481 | <0,001 |
|  | MTR | 55,405 | ,792 | 228,697 | 53,844 | 56,966 | <0,001 |
| Month 18 | STR | 50,979 | ,967 | 272,452 | 49,075 | 52,882 | <0,001 |
|  | MTR | 55,212 | ,811 | 240,972 | 53,613 | 56,810 | <0,001 |
| Month 24 | STR | 51,882 | 1,007 | 236,331 | 49,899 | 53,866 | <0,001 |
|  | MTR | 55,816 | ,825 | 232,363 | 54,191 | 57,441 | <0,001 |

**Treatment Satisfaction: Graph**


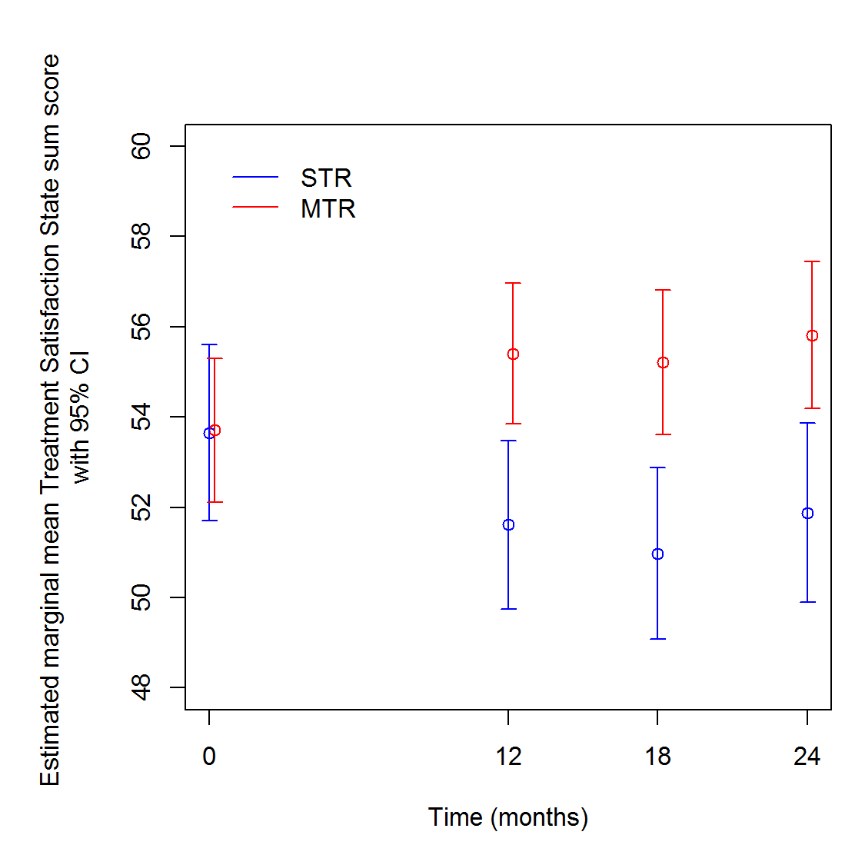


## Presence of neurocognitive complaints (Neurocognitive screening questions, yes/no, yes = worse outcome)

Generalized estimating equations model with exchangeable correlation matrix and time continuous.
QIC: 1537.167
QICC: 1508.960
94.5% of data were available (1244/1316).

| **Neurocognitive complaints: Parameter Estimates** | | | | | | | | | | |
| --- | --- | --- | --- | --- | --- | --- | --- | --- | --- | --- |
| Parameter | B | Std. Error | 95% Wald Confidence Interval | | Hypothesis Test | | | Exp(B) | 95% Wald Confidence Interval for Exp(B) | |
|  |  |  | Lower | Upper | Wald Chi-Square | df | Sig. |  | Lower | Upper |
| Intercept | -,840 | ,2056 | -1,243 | -,437 | 16,688 | 1 | ,000 | ,432 | ,289 | ,646 |
| Time continuous | -,021 | ,0069 | -,035 | -,008 | 9,384 | 1 | ,002 | ,979 | ,966 | ,992 |
| STR group | 2,902 | ,3576 | 2,201 | 3,603 | 65,871 | 1 | ,000 | 18,214 | 9,037 | 36,710 |
| MTR group | 0 | . | . | . | . | . | . | 1 | . | . |
| STR * Time continuous | ,099 | ,0181 | ,063 | ,134 | 29,666 | 1 | ,000 | 1,104 | 1,065 | 1,144 |
| MTR * Time continuous | 0 | . | . | . | . | . | . | 1 | . | . |
| Women | ,051 | ,4545 | -,840 | ,942 | ,013 | 1 | ,911 | 1,052 | ,432 | 2,565 |
| Men | 0 | . | . | . | . | . | . | 1 | . | . |
| Non-Caucasian | -,257 | ,5223 | -1,280 | ,767 | ,241 | 1 | ,623 | ,774 | ,278 | 2,153 |
| Caucasian | 0 | . | . | . | . | . | . | 1 | . | . |
| Heterosexual | ,008 | ,3561 | -,690 | ,706 | ,000 | 1 | ,982 | 1,008 | ,502 | 2,026 |
| Homosexual | 0 | . | . | . | . | . | . | 1 | . | . |
| (Scale) | 1 |  |  |  |  |  |  |  |  |  |

Intercept: Odds on NCC in MTR-group is 0.432 (p<0.001)
Time: ODDS of having NCC decreases by 2.1% in stable group per month (p=0.002)

Stable: At 2y: STR-groups has ODDS of 18.214 of having NCC as compared to MTR-group (p<0.001)

Interaction: As time increases 1 month, ODDS of having NCC increases with 10.4% among STR patients as compared to MTR group (p<0001). Other formulation: as time increases 1 month, ODDS of having NCC decreases with 10.4% as compared to STR-group

|  | **Neurocognitive complaints: Estimates** | | | | |  |
| --- | --- | --- | --- | --- | --- | --- |
|  | StableART | Mean | Std. Error | 95% Wald Confidence Interval | |  |
| Time |  |  |  | Lower | Upper | Sig |
| Baseline | STR | ,53 | ,080 | ,37 | ,67 | ,085 |
|  | MTR | ,39 | ,058 | ,29 | ,51 | ,085 |
| Month 1 | STR | ,55 | ,077 | ,39 | ,69 | ,033 |
|  | MTR | ,39 | ,058 | ,28 | ,51 | ,033 |
| Month 3 | STR | ,58 | ,072 | ,44 | ,71 | ,003 |
|  | MTR | ,38 | ,057 | ,28 | ,50 | ,003 |
| Month 6 | STR | ,64 | ,064 | ,51 | ,75 | <0,001 |
|  | MTR | ,36 | ,055 | ,26 | ,48 | <0,001 |
| Month 12 | STR | ,74 | ,053 | ,62 | ,83 | <0,001 |
|  | MTR | ,34 | ,054 | ,24 | ,45 | <0,001 |
| Month 18 | STR | ,82 | ,045 | ,71 | ,89 | <0,001 |
|  | MTR | ,31 | ,054 | ,21 | ,42 | <0,001 |
| Month 24 | STR | ,88 | ,038 | ,78 | ,93 | <0,001 |
|  | MTR | ,28 | ,054 | ,19 | ,40 | <0,001 |
|  |  | | | | |  |
|  |  | | | | |  |

**Neurocognitive complaints: Graph**


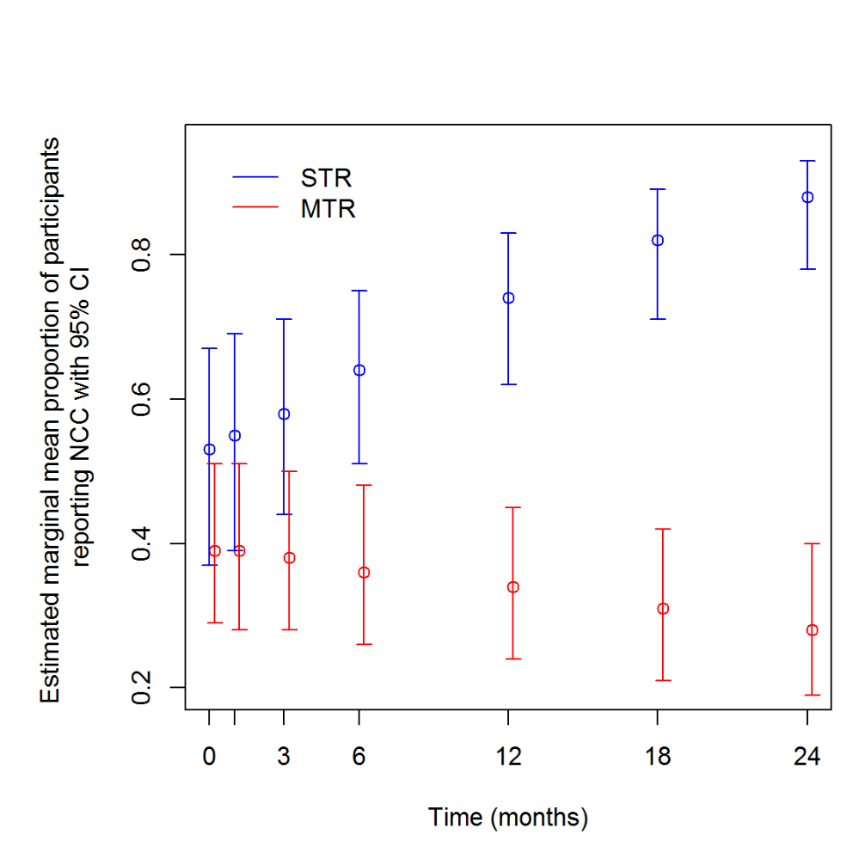

Supplement: S3 File — (DOCX) [file pone.0262533.s003.docx]
